# Novel Insights on Human Carbonic Anhydrase Inhibitors Based on Coumalic Acid: Design, Synthesis, Molecular Modeling Investigation, and Biological Studies

**DOI:** 10.3390/ijms23147950

**Published:** 2022-07-19

**Authors:** Virginia Pontecorvi, Mattia Mori, Francesca Picarazzi, Susi Zara, Simone Carradori, Amelia Cataldi, Andrea Angeli, Emanuela Berrino, Paola Chimenti, Alessia Ciogli, Daniela Secci, Paolo Guglielmi, Claudiu T. Supuran

**Affiliations:** 1 Department of Drug Chemistry and Technologies, Sapienza University of Rome, P.le A. Moro 5, 00185 Rome, Italy; virginia.pontecorvi@uniroma1.it (V.P.); emanuela.berrino@uniroma1.it (E.B.); paola.chimenti@uniroma1.it (P.C.); alessia.ciogli@uniroma1.it (A.C.); daniela.secci@uniroma1.it (D.S.); 2 Department of Biotechnology, Chemistry and Pharmacy, University of Siena, Via Aldo Moro 2, 53100 Siena, Italy; mattia.mori@unisi.it (M.M.); francesc.picarazzi@student.unisi.it (F.P.); 3 Department of Pharmacy, “G. d’Annunzio” University of Chieti-Pescara, Via dei Vestini 31, 66100 Chieti, Italy; susi.zara@unich.it (S.Z.); simone.carradori@unich.it (S.C.); amelia.cataldi@unich.it (A.C.); 4 NEUROFARBA Department, Pharmaceutical and Nutraceutical Section, University of Florence, Via Ugo Schiff 6, 50019 Florence, Italy; andrea.angeli@unifi.it; 5 Department of Food and Drug, University of Parma, Parco Area delle Scienze, 27/A, 43124 Parma, Italy

**Keywords:** coumalic acid, pyran-2-ones, docking, molecular dynamics, carbonic anhydrase inhibitors, breast adenocarcinoma cells (MCF7)

## Abstract

Human carbonic anhydrase (hCA, EC 4.2.1.1) isoforms IX and XII are overexpressed in solid hypoxic tumors, and they are considered as prognostic tools and therapeutic targets for cancer. Based on a molecular simplification of the well-known coumarin scaffold, we developed a new series of derivatives of the pyran-2-one core. The new compounds are endowed with potent and selective inhibitory activity against the tumor-related hCA isoforms IX and XII, in the low nanomolar range, whereas they are inactive against the two cytosolic off-targets hCA I and II. The compounds exhibiting the best hCA inhibition were further investigated against the breast adenocarcinoma cell line (MCF7) in hypoxic conditions, evaluating their ability to eventually synergize with doxorubicin. The compounds’ biocompatibility on healthy cells was also tested and confirmed on Human Gingival Fibroblasts (HGFs). Furthermore, the possible binding mode of all compounds to the active site of the tumor-associated human CA IX was investigated by computational techniques which predicted the binding conformations and the persistency of binding poses within the active site of the enzyme, furnishing relevant data for the design of tight binding inhibitors.

## 1. Introduction

**Figure 1:**
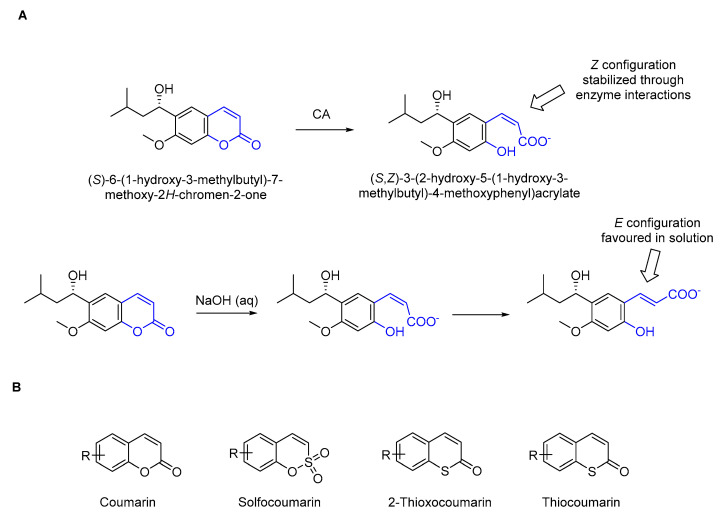
(**A**) Mechanism of action of 6-(1*S*-hydroxy-3-methylbutyl)-7-methoxy-2*H*-chromen-2-one. (**B**) Coumarin analogues.

The reversible hydration of carbon dioxide is an important reaction involved in a plethora of fundamental processes taking place in organisms from across the phylogenetic tree [1,2,3]. However, this quite simple reaction slowly occurs in physiological conditions, requiring the aid of an enzyme that strongly accelerates it. Carbonic anhydrases (CAs, EC 4.2.1.1) are ubiquitous metalloenzymes involved in the catalysis of this reaction, and currently eight diverse genetic CA families (*α*-, *β*-, *γ*-, *δ*-, *ζ*-, *η*-, *θ*-, and *i*-CAs) have been reported [4,5,6,7,8]. The fifteen human carbonic anhydrase isoforms (hCAs) belong to the *α*-family and cover a pivotal role in a multitude of physiological functions such as respiration, transport of carbon dioxide between metabolizing tissues and lungs, pH homeostasis, electrolyte secretion in various tissues/organs, as well as biosynthetic reactions [9]. However, these enzymes may also contribute to pathological processes when their dysregulated expression and/or abnormal activity occurs (e.g., in glaucoma, edema, epilepsy, altitude sickness, and cancer) [10,11,12,13]. Modulation of CA activity by making use of CA inhibitors and activators represents, therefore, a validated strategy for the treatment of several pathological states, with many derivatives developed so far and some of them in clinical use for decades [14,15,16]. CA inhibitors (CAIs), largely more explored than activators, have been used in clinics since the 1950s, but in recent years new therapeutic applications are emerging for these compounds, with particular relevance for tumor treatment [16,17,18,19]. hCA IX is a membrane-bound isoform with limited expression in healthy tissues (stomach and gallbladder epithelia), while it has been found overexpressed in many solid tumors often after hypoxia induction. hCA XII is a transmembrane isoform as well, displaying a wider tissue distribution when compared to the isoform IX; its overexpression, observed in some tumor types, is associated with less aggressive phenotypes if compared with tumors overexpressing the isoform IX. For their role in cancer growth, migration, and invasion, these two enzymes are called tumor-related isoforms (of hCA), and their selective inhibition can be exploited in order to obtain anticancer drugs able to exclusively affect the tumor cells [20,21,22,23,24]. Among the CAIs developed so far, the coumarin-based ones occupy a prominent place [21,25,26,27]. The first report describing the ability of this scaffold to inhibit hCAs dates back to 2008: a bioaffinity screening based on the electrospray ionization Fourier transform ion cyclotron resonance mass spectrometry (ESI-FTICR-MS) of some natural extracts demonstrated the ability of the 6-(1*S*-hydroxy-3-methylbutyl)-7-methoxy-2*H*-chromen-2-one to inhibit hCAs (Figure 1A) [28].

**Figure 2:**
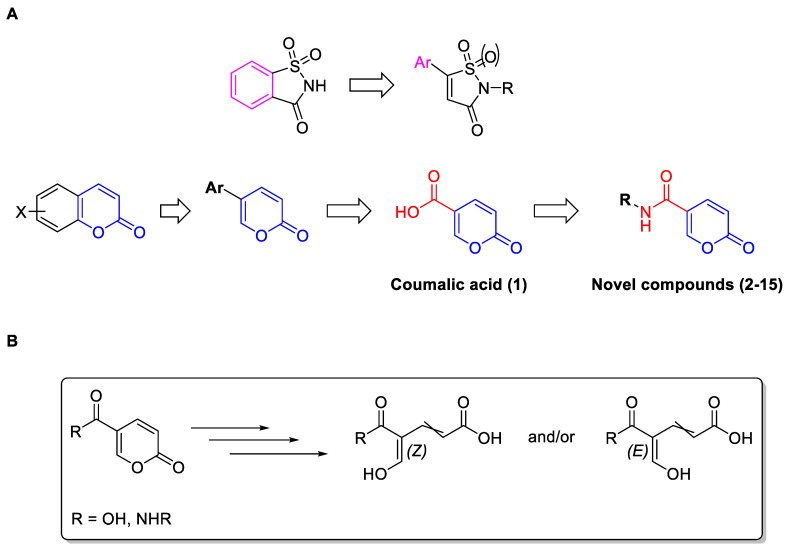
(**A**) Design of the novel compounds through saccharin and coumarin molecular simplification. (**B**) Possible hydrolysis pathway of compounds **1**–**15** up to the formation of the *Z-* or *E*-enol.

The following year, a detailed investigation of the inhibition mechanism of this compound, through X-ray crystallography and mass spectrometry approaches, led to the discovery of an unusual binding mode based neither on the coordination of Zn(II) ion nor on the anchoring of the water molecule bound to it (the two mechanisms known at that time) [29]. Surprisingly, the binding occurred at the entrance of the active site, and the structure found inside the hCA II did not correspond to the starting compound. The lactone portion of the coumarin core underwent hydrolysis by the Zn(II) bound hydroxide ion, leading to the cinnamic acid product (the opened form) that was the real inhibitor of the enzyme (Figure 1A). Albeit the *Z* isomer was observed from the X-ray study, the *E* one also obtained through prior aqueous hydrolysis exhibited comparable inhibitory activity. This discovery paved the way for intense efforts focused on the investigation of this scaffold able to bind further away from the Zn(II) ion, essentially at the entrance of the active site cavity, where the differences among the isoforms are higher [30,31,32]. Further studies evaluated the possibility to convert the coumarin ring to thiocoumarin, thioxocoumarin, or sulfocoumarin ones (Figure 1B) in order to explore the effects elicited by the substitution of the oxygen atom with the more lipophilic sulfur one (thiocoumarin and thioxocoumarin) or the outcomes coming from lactone moiety exchange with sulfonic one (sulfocoumarin). These analogues led to the discovery of alternative binding modes inside the hCA active site. Sulfocoumarins underwent hydrolysis similar to the parent coumarins. However, X-ray crystal analysis evidenced that the obtained vinyl sulfonic acid isomerized from the *Z* to the *E* configuration; moreover, the inhibitor bound the hCA II by anchoring the zinc-coordinated water through the sulfonic acid moiety, differing from the classical binding mode of the firstly reported coumarins [33]. A further binding mode was also observed for thioxocoumarins, these inhibitors being found in the active site in the non-hydrolyzed form, anchoring the zinc-coordinated water through the sulfur atom of the thiolactone moiety [34]. Recently, Sapi and co-workers proposed an interesting molecular simplification of saccharin [35], a well-known scaffold employed by us and other researchers to develop hCA inhibitors [36,37,38,39]. Particularly, the removal of the bicyclic system of the lead compound saccharin, maintaining the 5-arylisothiazol-3(2*H*)-one core as mono or dioxides at the sulfur atom, led to interesting new classes of CAIs (Figure 2A) [35]. The disconnected benzene ring was bound at the carbon 5 of the five-member heterocycle arylisothiazol-3(2*H*)-one. Furthermore, heterocycles were also evaluated in that position [35]. The obtained compounds were evaluated against hCAs to assess whether these structural modifications could affect their inhibitory properties. The derivatives displayed effective inhibitory activity against the two tumor-related isoforms hCA IX and XII, demonstrating absent or scarce inhibition of the two off-targets hCA I and II. Taking into account this evidence, we evaluated here the opportunity to apply a similar approach on the coumarin scaffold, performing a molecular simplification aimed to retain only the cyclic scaffold containing the lactone moiety, which is the molecular fragment mainly involved in the CA inhibition mechanism (Figure 2A).

The novel compounds, obtained from the commercially available coumalic acid (**1**), preserved the 2*H*-pyran-2-one core of the coumarin scaffold (blue in Figure 2) that should represent the molecular attribute responsible for the inhibition of hCAs, through (i) its putative hydrolysis provoked by the enzyme and successive binding in the entrance of the active site (Figure 2B), or (ii) anchoring the zinc-bound water in its hydrolyzed/not-hydrolyzed form. The position 5 of this core has been gifted with a carboxamide linker (red in the Figure 2A), bearing different (un)substituted aromatic or (hetero)cycloaliphatic residues aimed at expanding the chemical space exploitable for the development of new and effective hCA inhibitors.

## 2. Results and Discussions

### 2.1. Chemistry

#### 2.1.1. Synthesis of Compounds **2**–**15**

**Scheme 1:**
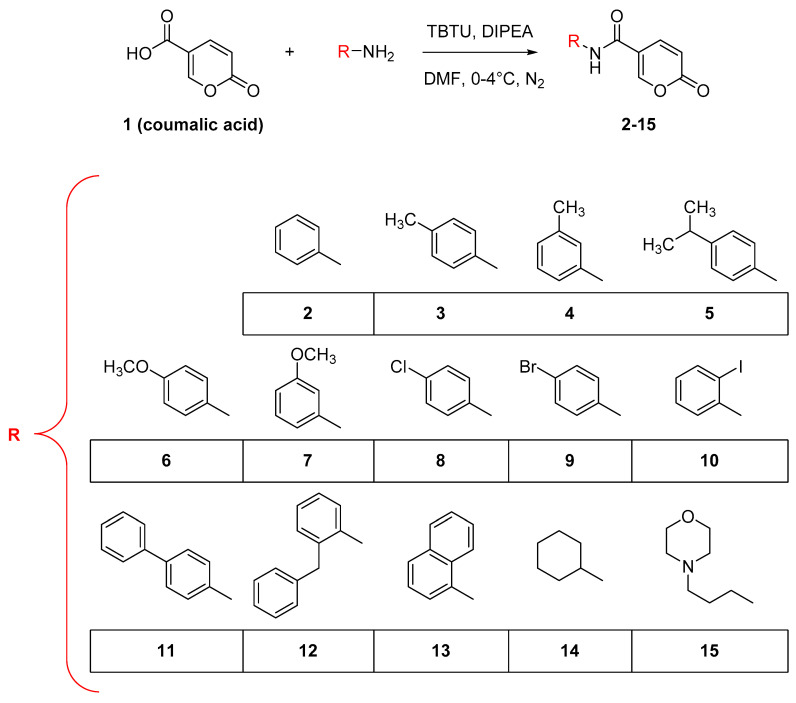
Synthetic approach employed for the synthesis of the compounds **2**–**15**.

The syntheses of compounds **2**–**15** were performed exploiting the coupling reaction between coumalic acid with proper (un)substituted aromatic or (hetero)cycloaliphatic amines (Scheme 1). The reactions were performed in *N*,*N*′-dimethylformamide (DMF) in the presence of TBTU (2-(1*H*-benzotriazole-1-yl)-1,1,3,3-tetramethylaminium tetrafluoroborate) as the coupling agent and an excess of diisopropylethylamine (DIPEA) at ice-bath temperature (0–4 °C); reaction times spanned from 24 to 48 h depending on the amine reactivity. After completion of reactions, the mixture underwent a general work-up (extraction or filtration) followed by purification performed through column chromatography and crystallization. The purity of compounds **2**–**15** was assessed by means of chromatographic analysis (HPLC). A gradient elution approach, with a binary mobile phase composed of water and methanol, was employed. All the analyzed compounds were >95% HPLC pure (Supplementary Material, Figure S2).

#### 2.1.2. NMR Studies

**Figure 3:**
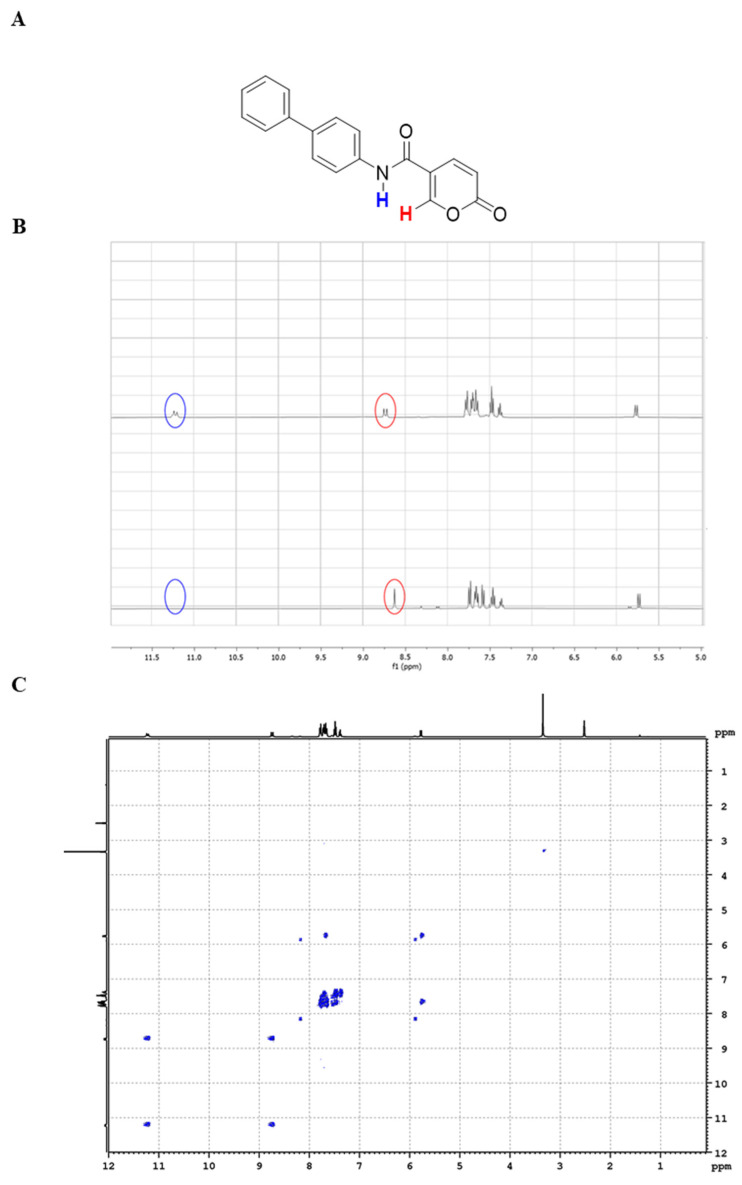
(**A**) Structure of compound **11**, involved in the NMR studies. (**B**) ^1^H-NMR spectrum of compound without (high) and after (downward) D_2_O addition. (**C**) ^1^H-^1^H NMR-COSY spectrum of compound **11** (the peaks at 2.50 and at 3.33 ppm are related to solvents).

The purified compounds were fully characterized by spectroscopic data (^1^H and ^13^C NMR), displaying at a first ^1^H NMR analysis four recurrent doublets at ~ 5.00–6.00 ppm (*J* ~ 8.0–9.0 Hz); ~7.00–8.00 ppm (*J* ~ 8.0–9.0 Hz); ~8.00–9.00 ppm (*J* ~ 13.0–14.0 Hz), identified as the protons of the pyran-2-one core; and at ~10.00–12.00 ppm (*J* ~ 13.0–14.0 Hz), identified as the amidic proton (blue in the Figure 3A). In fact, almost all the derivatives exhibited the latter as a doublet with coupling constant values ranking from 13.0 to 14.0 Hz, correlating with another doublet attributed to the proton bound at position 6 of the pyran-2-one core (red in Figure 3A). In order to deeply investigate the structure of the inhibitors and untangle the real nature of the doublets, some compounds (**5**, **11** and **14**) were further explored through ^1^H NMR in the presence of D_2_O to investigate the proton exchange: ^1^H-^1^H NMR-COSY (Correlation Spectroscopy), ^1^H-^1^H NMR-NOESY (Nuclear Overhauser Effect Spectroscopy), and ^1^H–^13^C HSQC (Heteronuclear Single Quantum Correlation), to confirm proton attribution. For the sake of brevity, here we report only some of the results of compound **11** (for the other compounds please see Supplementary Materials). The ^1^H NMR spectrum shown in Figure 3B (high) was carried out without the addition of D_2_O, exhibiting the doublet at ~11.00 ppm. In presence of D_2_O (Figure 3B, downward), this signal was absent, confirming the exchangeable nature of this proton, identified as the amidic one. At the same time, the doublet attributed to the proton bound in position 6 of the pyran-2-one core becomes a singlet, confirming the hypothesized correlation between the two protons (Figure 3B, downward). This correlation was also investigated through an ^1^H-^1^H NMR-COSY experiment (Figure 3C), as well as the assignment of the proton peaks belonging to the pyran-2-one core: the resulting cross peaks between the signals at 8.74 and 11.22 ppm confirmed the coupling between the amidic proton and the one in position 6 of the pyran-2-one core (Figure 3C). The interaction between the different protons was also assessed through ^1^H-^1^H NMR-NOESY and ^1^H-^13^C HSQC (Supplementary Materials), confirming the ^1^H-^1^H coupling and the peak assignments. All the ^1^H and ^13^C NMR of the synthesized compounds are available in the Supplementary Material file.

### 2.2. Biological Results

#### 2.2.1. CA Inhibitory Activity of the Target Compounds

Compounds **1**–**15** were evaluated for their ability to inhibit four isoforms of carbonic anhydrase: the two off-targets hCA I and II and the two tumor-related isoforms hCA IX and XII (targets), by applying a stopped flow CO_2_ hydrase assay (Enzyme inhibition curves of compounds **1–15** are available in the Supplementary Material file) [9]. The data reported in Table 1 display the ability of all the derivatives to selectively inhibit hCA IX and XII, being devoid of activity against hCA I and II (*K*_I_ hCA I, II > 100 µM).

Differences in hCA IX and XII inhibition have been observed among the proposed derivatives, depending on carboxylic group substitution of the coumalic acid. The simplest derivative **1** (coumalic acid) exhibited the best inhibitor profile, being endowed with low nanomolar activity against both hCA IX (*K*_I_ = 0.073 µM) and XII (*K*_I_ = 0.083 µM); moreover, this was the most potent inhibitor of hCA IX of the entire series. Taking advantage of the coupling reaction with (un)substituted aromatic or (hetero)cycloaliphatic amines, we turned the carboxylic group to a carboxamide one endowed with moieties that elicited different results. The unsubstituted phenyl ring (compound **2**) negatively affected the affinity towards both the isoforms; in particular, hCA IX suffered most with this change with respect to hCA XII (*K*_I_ hCA IX = 3.000 µM; *K*_I_ hCA XII = 0.700 µM). The introduction of a methyl substituent at the para position of the phenyl ring (compound **3**) did not elicit improvements with *K*_I_ values, resembling those reported for derivative **1**. On the other hand, the displacement of the methyl group from the para to meta position (compound **4**) dramatically increased the potency, the isoforms hCA IX and XII being inhibited in the low nanomolar and high nanomolar ranges, respectively (*K*_I_ hCA XII = 0.090 µM; *K*_I_ hCA IX = 0.174 µM). A similar trend was also observed for the derivatives **6** and **7**, endowed with a para and meta-methoxy substituted phenyl ring, respectively. Indeed, the shift of the substituent from the para to meta position led to a ~10-fold increase in hCA IX inhibition and ~3-fold increase for hCA XII. In general, the methyl substituent was better tolerated than the methoxy one, regardless the site on the phenyl ring. The para-chloro-substituted derivative 8 displayed reduced affinity against both the tumor-related isoforms, with *K*_I_ values that are comparable with those observed for compounds **2** and **6**. However, the increasing of the lipophilicity and dimensions of the halogen, through the introduction of a bromine instead of chlorine atom (as for compound **9**), restored the nanomolar inhibitory activity with similar potency against both the target isoforms (*K*_I_ hCA IX = 0.082 µM; *K*_I_ hCA XII = 0.089 µM). Compound **5**, endowed with the bulky and lipophilic isopropyl group located at the para position of the phenyl ring, exhibited sub-micromolar inhibitory activity against isoform IX (*K*_I_ hCA IX = 0.273 µM), while the inhibition of hCA XII dropped to a nanomolar level, leading to the best hCA XII inhibitor of the series (*K*_I_ hCA XII = 0.068 µM). In the light of the observations relative to compounds **5** and **9,** it seemed that the para position can tolerate bulky and lipophilic groups. As a matter of fact, one of the most potent inhibitors of the series, **11**, was obtained by means of the coupling of coumalic acid with para-phenylaniline (*K*_I_ hCA IX = 0.083 µM; *K*_I_ hCA XII = 0.076 µM). On the other hand, the presence of the bulky naphthyl group in compound **13** led to opposed response by the enzymes with the hCA IX that slightly “recognized” the molecule (*K*_I_ hCA IX = 3.689 µM), while the isoform XII was effectively inhibited at the nanomolar level (*K*_I_ hCA XII = 0.094 µM). Therefore, the spatial distribution of the bulky substituent on the phenyl ring also seemed to play a role for hCA IX enzyme recognition. The substitution of the ortho position of the benzene ring was challenged with an iodine (**10**) or benzyl group (**12**). Both the substituents provoked the insurgence of a slight preference for isoform XII. The last two compounds, **14** and **15**, were endowed with cyclohexyl and 2-morpholinetanyl groups, respectively. These (hetero)cycloaliphatic moieties remarkably influenced the inhibitory activity of these molecules, which exhibited equal affinity towards hCA XII (*K*_I_ hCA XII = 0.087 µM). On the contrary, the introduction of the ethyl chain as well as the presence of the N and O heteroatoms of the morpholine ring was detrimental for inhibition of hCA IX, compound **15** being the sub-micromolar inhibitor of this isoform. The presence of the simple cyclohexyl ring, instead, produced a potent hCA IX inhibitor with *K*_I_ hCA IX = 0.098 nM. Based on the *K*_I_ values assessed through in vitro enzymatic assays, we selected compounds **1**, **11** and **14** for further studies.

#### 2.2.2. Biological Evaluation of Breast Adenocarcinoma Cells (MCF7)

**Figure 4:**
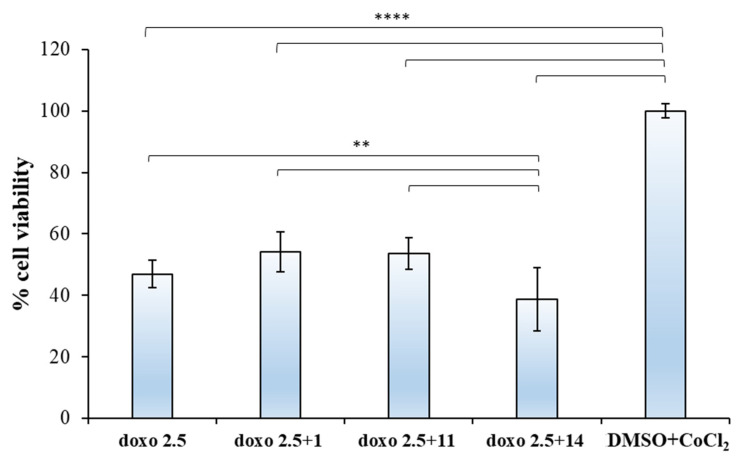
MTT assay on MCF7 cell line. Histogram represents the viability percentage of MCF7 cells exposed to doxorubicin 2.5 μM and to combinations of doxorubicin 2.5 μM with compounds **1**, **11**, and **14**, all administered at 100 μM for 72 h and under hypoxic conditions. Cell viability was measured by MTT assay and normalized to control cells treated with DMSO (0.1% as final concentration) + CoCl_2_ 100 μM. The most representative of three separate experiments is shown. Data are presented as the mean ± standard deviation. **** *p* < 0.0001; ** *p* < 0.01.

The compounds exhibiting the best results in the hCA inhibition assay (**1**, **11** and **14**) were further investigated against the MCF7 breast adenocarcinoma cell line, previously exposed to CoCl_2_ for 48 h in order to mimic a hypoxia condition. These compounds were evaluated for their ability to negatively or positively affect the effect of doxorubicin on MCF7 viability, by administering doxorubicin at 2.5 µM (doxo 2.5) and 1.25 µM (doxo 1.25) concentrations and the newly synthesized molecules at 100 µM. The metabolic activity was evaluated through the MTT test after 72 h of treatment. MCF7 viability appears significantly reduced in all tested samples (doxo 2.5, doxo 2.5 + **1**, doxo 2.5 + **11**, and doxo 2.5 + **14**) with respect to control cells (cells exposed to DMSO + CoCl_2_); in parallel, when doxorubicin is administered in combination with compound **14**, a statistically significant reduction in cell viability percentage is recorded with respect to doxorubicin 2.5 µM alone (Figure 4).

**Figure 5:**
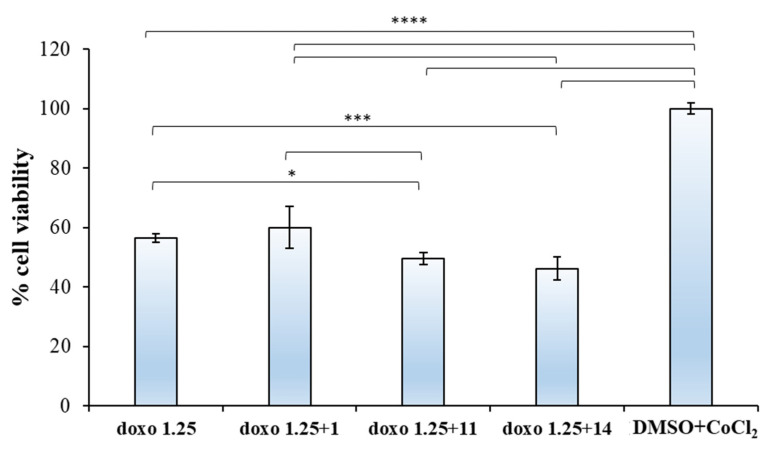
MTT assay on MCF7 cell line. Histogram represents the viability percentage of MCF7 cells exposed to doxorubicin 1.25 μM and to combinations of doxorubicin 1.25 μM with compounds **1**, **11**, and **14**, all administered at 100 μM for 72 h and under hypoxic conditions. Cell viability was measured by MTT assay and normalized to control cells treated with DMSO (0.1% as final concentration) + CoCl_2_ 100 μM. The most representative of three separate experiments is shown. Data are presented as the mean ± standard deviation. **** *p* < 0.0001; *** *p* < 0.001; * *p* < 0.1.

Next, doxorubicin alone and in combination with the newly synthesized compounds was administered at 1.25 µM, finding that all tested conditions (doxo 1.25, doxo 1.25 + **1**, doxo 1.25 + **11**, and doxo 1.25 + **14**) provoke a significant reduction in cell viability with respect to the control sample. Furthermore, an appreciable reduction in cell viability is detected when the combinations of doxorubicin 1.25 µM + **11** and doxorubicin 1.25 µM + **14** are administered to MCF7 cells with respect to doxorubicin 1.25 µM alone (Figure 5).

**Figure 6:**
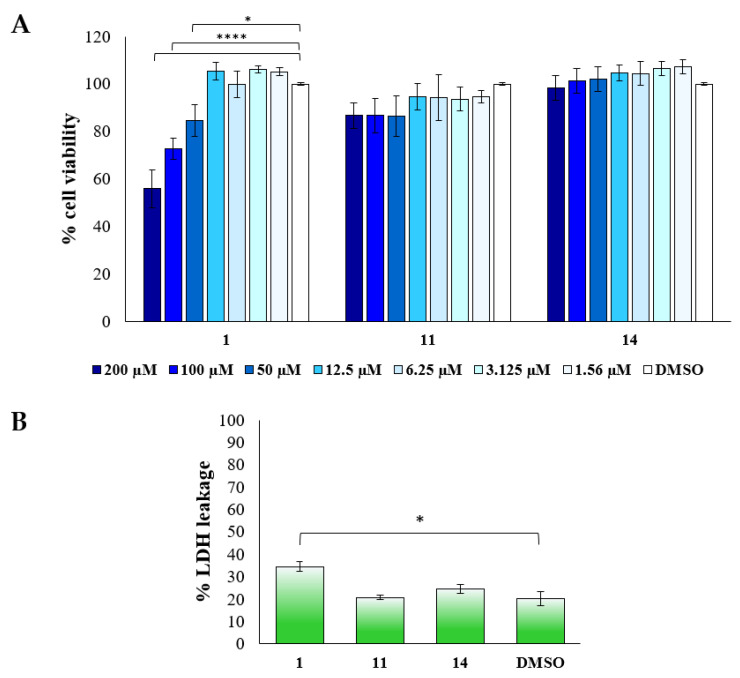
(**A**) MTT assay on HGFs exposed to compounds **1**, **11**, and **14**, all administered at doses ranging from 1.56 to 200 μM for 48 h. Cell viability was measured by MTT assay and normalized to control cells treated with DMSO (0.15% as final concentration). The most representative of three separate experiments is shown. Data are the mean ± standard deviation. **** vs. DMSO *p* < 0.0001; * vs. DMSO *p* < 0.1. (**B**) Cytotoxicity assay performed on HGFs treated with **1**, **11**, **14**, and control vehicle (DMSO), all administered at 200 μM for 48 h. LDH leakage is reported as percentage. Data are presented as the mean ± standard deviation of three separate experiments. * vs. DMSO *p* < 0.1.

Then, in order to exclude the toxicity of compounds **1**, **11**, and **14** on healthy cells, the metabolic activity, along with the membrane integrity, were evaluated on primary Human Gingival Fibroblasts (HGFs) (Figure 6).

In particular, HGFs were exposed to increasing concentrations of **1**, **11**, and **14**, ranging from 1.56 μM to 200 μM, for 48 h. The metabolic activity was evaluated by means of the MTT test. In the presence of **1**, cell viability percentage appears significantly reduced with respect to DMSO when the molecule is administered at 200, 100, and 50 μM; however, the percentage of viable cells never goes below 50%. When HGFs are exposed to **11** and to **14**, there is not a statistically significant reduction in cell viability with respect to DMSO at any administered concentration (Figure 6A), thus leading to assume that an appreciable rate of viable healthy cells is also definitely maintained when newly synthetized molecules are administered at high concentrations.

To confirm the biocompatibility of newly synthetized compounds on non-tumoral cells, a cytotoxicity test, measuring the Lactate Dehydrogenase (LDH) release within the culture medium and thus providing information regarding the cell membrane integrity, was carried out. LDH assay was performed on HGFs treated with **1**, **11**, and **14** at the higher dose (200 μM), finding a significant increase in LDH release percentage in the presence of compound **1** with respect to DMSO, even if it still appears lower than 40%. LDH release in the presence of **11** and **14**, instead, appears comparable to the cytotoxicity level measured in the presence of the vehicle DMSO (Figure 6B), leading to exclude toxic effects on healthy cells.

These data show the moderate ability of compounds **11** and **14** to enhance the antitumoral activity of doxorubicin, while coumalic acid (**1**) did not improve the reduction in cellular viability after co-treatment with doxorubicin. Interestingly, in both the experiments the best results were elicited by compound **14**, which exhibited a significant reduction in cell viability. Doxorubicin is extremely efficient in vitro when given as monotherapy; however, its cardiotoxicity in vivo is well documented at therapeutic levels. In this regard, employing hCA IX and XII inhibitors as putative chemosensitizers or co-adjuvants could lead to reduction in antitumoral drug concentration and thus limit the possibility to develop harmful side effects. The promising capability of compounds **11** and **14** to strengthen the effect of doxorubicin appears further valuable if the results obtained on healthy cells are also taken into consideration. In this study, we shed light on the fact that the viability rate of non-tumoral cells is not significantly affected by **11** and **14** administration, as the percentage of viable cells is always maintained above 60%. On the other hand, we also demonstrated that a cytotoxic effect, especially for compounds **11** and **14,** can be definitely excluded, even when treatments are performed at very high doses.

### 2.3. Molecular Modeling

The possible binding mode of compounds **1**–**15** to the active site of the tumor-associated human CA IX was investigated by molecular modeling simulations. Specifically, molecular docking was used to predict the binding conformation of the molecules, while molecular dynamics (MD) simulations were employed to challenge the persistency of docking-based poses and to sample the conformational space of protein–ligand complexes.

#### 2.3.1. Molecular Docking

Compounds **1**–**15** were docked to the crystallographic structure of hCA IX by the GOLD program using previous settings [40]. However, considering the potential hydrolysis of the lactone moiety due to the esterase activity of hCAs, the open enolic form of **1**–**15** with both the *E* and *Z* configuration of the double bond was docked as well (Figure 2B).

**Figure 7:**
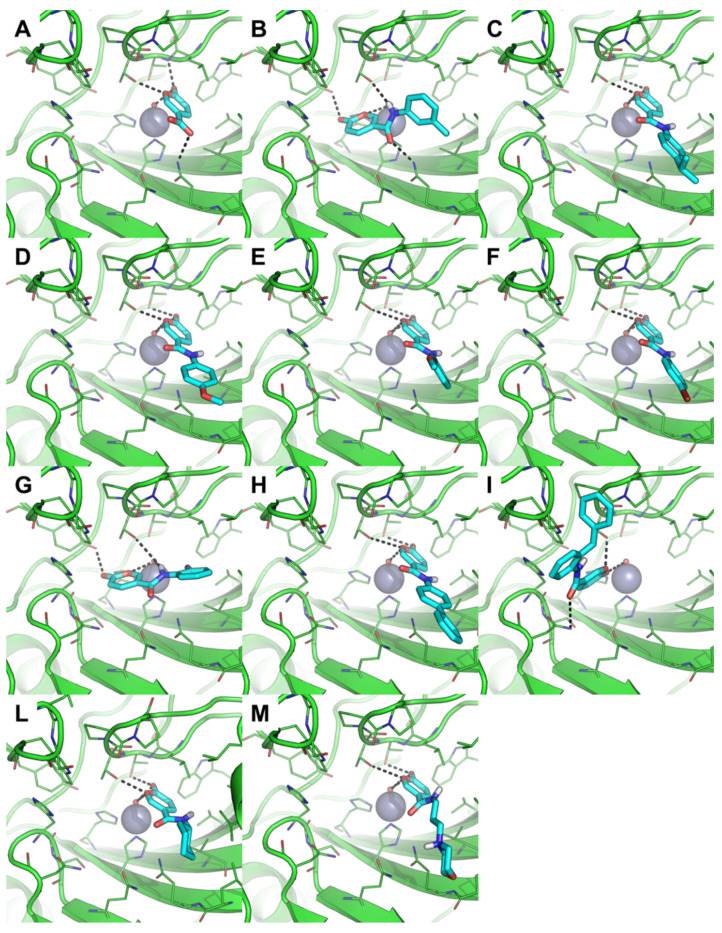
Docking-based binding mode of compounds **1** (**A**), **4** (**B**), **5** (**C**), **6** (**D**), **7** (**E**), **9** (**F**), **10** (**G**), **11** (**H**), **12** (**I**), **14** (**L**), and **15** (**M**) within the catalytic site of hCA IX crystallographic structure (PDB-ID: 3IAI) [48]. The protein is shown as green cartoon and lines, residues at a distance higher than 5 Å from the inhibitors are omitted for the sake of clarity. Small molecules are shown as cyan sticks, polar interactions are highlighted by black dashed lines. The Zn(II) ion is shown as a grey sphere, while the coordinated water molecule is represented as a small red sphere.

In analogy with crystallographic results obtained for similar pharmacophores, the Zn(II)-bound water molecule was kept as a part of the receptor during molecular docking simulations [27,33,34,41,42]. Docking results showed that all compounds but **2**–**3**, **8**, and **13** are able to fit the hCA IX catalytic site and to anchor the Zn(II)-bound water molecule through their lactone moiety. Notably, these compounds are characterized by the strongest inhibition constant among the test set, as underlined in Table 1, which suggests that the recognition of the closed form of the compounds is crucial to achieve strong hCA IX inhibition (Figure 7). Indeed, compounds having a weaker inhibitory activity towards hCA IX such as **2**, **3**, **8,** and **13** were unable to fit the catalytic site of the enzyme and to anchor the Zn(II)-bound water molecule in docking simulations (data not shown). The sole exception to this rule is represented by compound **6**, which proved able to fit the hCA IX catalytic site similarly to stronger inhibitors, despite having a modest inhibitory activity towards the enzyme (Figure 7D). Although compound **1** is endowed with two chemical moieties that are potentially able to H-bond the Zn(II)-coordinated water molecule, i.e., the carboxylic group and the lactone ring, the most relevant pose shows the preferential orientation of the lactone ring in proximity of the metal coordination site (Figure 7A). All the inhibitors, devoid of a phenyl ring (compound **1**) or having the para-substituted phenyl ring (compounds **5**, **6**, **9**, **11**) as well as (hetero)cycloaliphatic moieties (compounds **14** and **15**), bind in a very similar manner, being able to H-bond the Zn(II)-bound water molecule as well as Thr199 and Thr200. Notably, these residues are contacted also by the reference co-crystallized inhibitor AAZ and by several potent hCA inhibitors [43,44,45,46,47].

The lipophilic part of the compounds binds near the entrance of the catalytic site, in proximity of a lipophilic Leu/Val-rich cluster composed of Leu91, Val121, Leu123, Val131, Leu135, Leu141, Val143, and Leu198. In contrast, compounds having the substituent in the ortho or meta position of the phenyl ring (compounds **4**, **10**, and **12**) bind with a different orientation on the opposite site of the catalytic site, where they establish H-bonds with Tyr7, Asn62, Thr200, and Gln92. Compound **7** represents an exception to this rule, as it bears a methoxyl group in the meta position but binds similarly to para-substituted derivatives (Figure 7).

**Figure 8:**
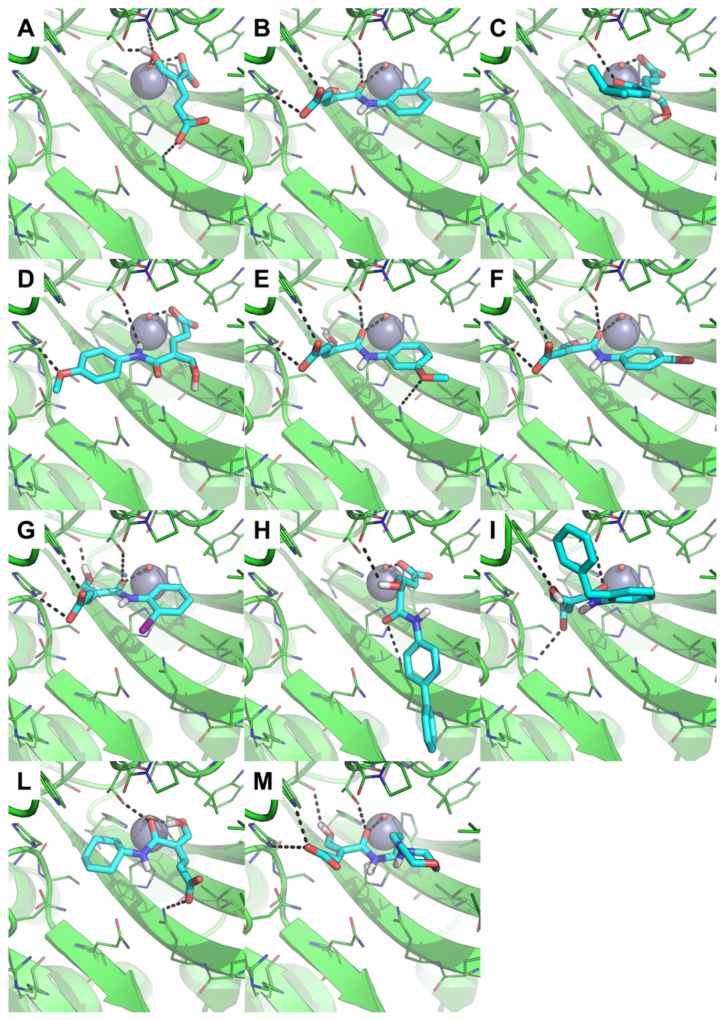
Docking-based binding mode of the *Z*-enolic open form of compounds **1** (**A**), **4** (**B**), **5** (**C**), **6** (**D**), **7** (**E**), **9** (**F**), **10** (**G**), **11** (**H**), **12** (**I**), **14** (**L**), and **15** (**M**) within the catalytic site of hCA IX crystallographic structure (PDB-ID: 3IAI) [48]. The protein is shown as green cartoon and lines, residues at a distance higher than 5 Å from the inhibitors are omitted for the sake of clarity. Small molecules are shown as cyan sticks, polar interactions are highlighted by black dashed lines. The Zn(II) ion is shown as a grey sphere, while the coordinated water molecule is represented as a small red sphere.

Docking of **1**–**15** in the open forms clearly suggested that the *Z* configuration of the enol moiety is largely preferred to fit the hCA IX catalytic site and to properly anchor the Zn(II)-bound water molecule (Figure 8) compared to the *E* configuration. Results obtained by docking the *E* configuration of **1**–**15**, and those obtained by docking the Z configuration of the weak inhibitors **2**, **3**, **8**, and **13** that proved unable to bind in the closed form, are not discussed herein.

Compounds in the open form bind the hCA IX catalytic site in two different conformations: (i) the carboxylic acid group binds to the Zn(II)-coordinated water molecule (compounds **1, 5, 6**, and **11**); (ii) the oxygen atom of the amide group, which is also engaged in an intramolecular H-bond with the enolic hydroxyl group (compounds **7**, **9**, **10**, **14**, and **15**), is H-bonded to the Zn(II)-coordinated water molecule. Compound **12** binds with a similar orientation as this latter group of inhibitors, but it is unable to contact the Zn(II)-bound water molecule most likely because of the steric hindrance of the bulky aromatic tail. In addition to interacting with the water molecule, all the compounds but **11** are H-bonded to Thr200. Additional interactions with Trp5, Tyr7, Asn62, His64, Gln92, Thr199, and Pro201 were observed (Figure 8), with a remarkable overlapping with the interaction network established by the AAZ and glycerol molecules within the hCA IX catalytic site in the reference crystallographic structure used in this work [48].

Overall, docking simulations suggested that effective hCA IX inhibitors tested in this work might be recognized in their closed lactone form by the enzyme, which then could hydrolyze the molecules through its esterase activity. Notably, the inhibitors can also retain the main contact within the hCA IX active site after the potential hydrolysis, which correlates with the enzyme inhibition data described in Table 1 with the only exception of compound **6**.

#### 2.3.2. MD Simulation

**Figure 9:**
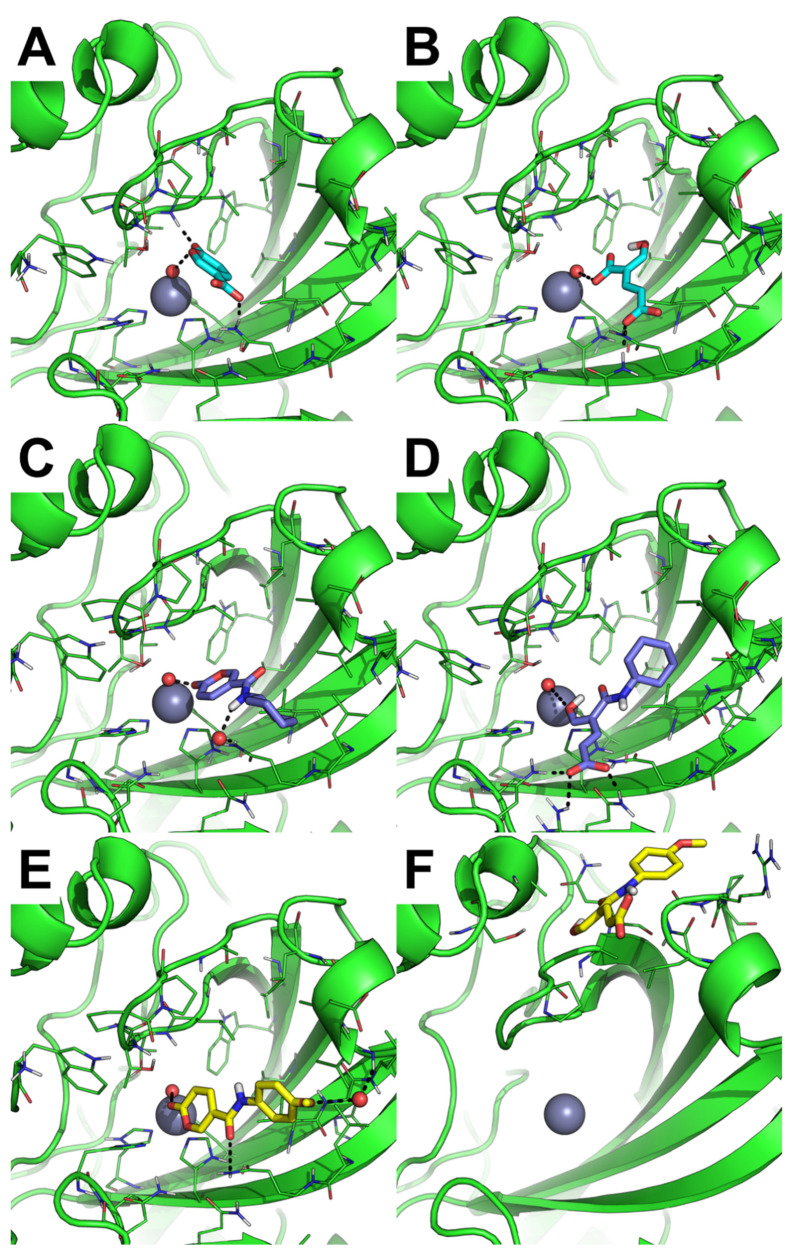
Predicted binding mode of compounds **1** (**A**,**B**), **14** (**C**,**D**), and **6** (**E**,**F**) in their open and closed forms as investigated through 500 ns of MD simulations. The representative frame extracted from MD trajectory by cluster analysis is shown in each panel. The protein is shown as green cartoon and lines, residues at a distance higher than 5 Å from the ligand are omitted for the sake of clarity. Compound **1** is shown as cyan sticks, compound **14** as blue sticks, compound **6** as yellow sticks. The catalytic Zn(II) ion is shown as a grey sphere, water molecules bridging the protein and the ligand are shown as small red spheres, all other water molecules and counter-ions were omitted. Polar interactions are highlighted by black dashed lines.

To further investigate the conformational persistency of the binding modes identified by docking, MD simulations were run on complexes formed by hCA IX and the compounds in both their closed and open *Z*-enolic forms. Specifically, compounds **1**, **6**, and **14** were investigated because (i) **1** is the most potent inhibitor of hCA IX investigated in this work, and it is a bifuctionalized ligand potentially able to anchor the metal coordination center by the carboxylic acid or by the lactone moiety; (ii) **6** represents an exception in docking results, being able to fit the catalytic site of hCA IX in its closed and open form, although showing a weak inhibition of the enzyme in experimental studies (Table 1); (iii) **14** is among the strongest inhibitors of this series and it is the only one bearing a lipophilic aliphatic carbon ring. For each complex, MD trajectories were generated for 500 ns in explicit water solvent. Representative binding poses extrapolated from MD trajectories by cluster analysis show a binding mode that is highly comparable to that predicted by molecular docking (Figure 9). Compound **1** binds preferentially to the Zn(II)-coordinated water molecule by the lactone moiety, while the carboxylic acid group is projected towards the entrance of the catalytic site where it establishes an H-bond with Gln92 (Figure 9A). This binding mode is highly superimposable to the crystallographic pose of the reference inhibitor AAZ (Supplementary Materials, Figure S1), as well as with the docking-based binding mode (Figure 7A). Compound **14** interacts with the metal coordination center by its lactone ring, while the amide moiety establishes a water-bridged H-bond with Gln92 (Figure 9C). Notably, this feature was not accessible in molecular docking simulations because the solvent was considered in an implicit manner. Finally, interaction with Gln92 is also preserved by the closed form of **6**, while the methoxyl tail of the molecule establishes a water-bridged interaction with the backbone of Val131 near the entrance of the catalytic site (Figure 9E).

MD simulations on the open *Z*-enolic forms of **1** and **14** confirmed the ability of the compounds to bind persistently within the hCA IX catalytic site in a pose that is highly comparable to the binding pose of their closed form, as well as to docking results (Figure 9B–D). In contrast, compound **6** detached from its docking site and moved into the bulk solvent before to interact stably within a solvent-exposed sub-pocket bounded by residues 202–204 and 135–137 on the top of the catalytic site (Figure 9F). However, ligand binding to this site has never been described before, nor associated with hCA inhibition, which correlates with the weak inhibitory activity discussed above (Table 1). Overall, MD simulations overcame the possible limitations of molecular docking by providing a dynamic picture of the conformational evolution of hCA IX/inhibitor complexes over time. MD results nicely confirmed the preference of these molecules for binding to the Zn(II)-coordinated water molecule and not directly to the metal ion, which corroborates the main binding hypothesis used in molecular docking simulations. Moreover, MD results clearly highlighted that potent hCA IX inhibitors **1** and **14** are able to fit the active site of the enzyme and to anchor the Zn(II)-bound water molecule in both their closed and open conformations, suggesting that they can inhibit the catalytic function of hCA IX also after the potential hydrolysis of the lactone moiety. Of note, the open form of the weak inhibitor **6** proved unable to fit the hCA IX catalytic site in MD simulations, indicating that the esterase activity of hCA IX might potentially inactivate this inhibitor. Taken together, these theoretical results correlate with experimental data and suggest a rational strategy to design hCA IX inhibitors endowed with functional groups that might potentially be hydrolyzed by hCAs esterase activity, still retaining hCA inhibition.

## 3. Materials and Methods

### 3.1. General Remarks

All the reagents, coumalic acid, and starting material employed in the synthetic procedures as well as HPLC analysis were obtained from commercial suppliers and were used without further purification. All melting points were reported uncorrected and measured on a Stuart^®^ melting point apparatus SMP1. ^1^H, ^13^C, COSY, NOESY, and HQSC NMR spectra were recorded at 400 MHz or 600 MHz on a Bruker spectrometer using the proper deuterated solvent (DMSO-*d*_6_) at room temperature. The processing and analyses of the NMR data were carried out with MestreNova. Chemical shifts are expressed as *δ* units (parts per millions) relative to the solvent signal and the coupling constants *J* are reported in Hertz (Hz). Thin layer chromatography (TLC) performed on 0.2 mm thick silica gel–aluminum backed plates (60 F_254_, Merck) was employed to monitor all the reactions. Preparative flash column chromatography was carried out on silica gel (230–400 mesh, G60 Merck). The yields shown are not optimized. Drying of organic solution was accomplished with anhydrous sodium sulfate. Evaporation of the solvent after reaction was carried out on a rotary evaporator.

The purity of compounds **2**–**15** was assessed through HPLC analyses, performed with the Shimadzu *Prominence*-*i* LC-2030C 3D system endowed with an autosampler, quaternary pump, column oven, and photodiode array detector (PDA) [9,23]. The resolution was achieved by using a C18 column (Kinetex EVO, C18, 5 µm, 100 Å) as stationary phase. The mobile phase gradient was obtained by mixing water (solvent A) and methanol (solvent B), at a constant flow of 1.00 mL min^−1^. The eluent composition was 35% of solvent B, at first kept constant for 2 min then raised up to 80% at 5 min and maintained at this value for another 3 min. At the end of this time, the concentration returned to the initial value for the column reconditioning (5 min), for a total run time of 15 min.

Due to the prompt elution of compound **15**, this was analyzed with a slightly modified method, based on the initial 10% of solvent B, maintained constant for 3 min, then raised up to 40% and maintained at this value for another 7 min. After this time, the percentage returned to the starting value of 10% of solvent B for the column reconditioning (5 min), for a total run of 15 min. All the analytes were dissolved in acetonitrile at a concentration of about 0.5–1 mg mL^−1^, depending on solubility. Then, 2 µL was directly injected for the HPLC analysis after sample preparation and the acquisitions accomplished at the wavelength of 254 nm. For the sake of uniformity, all the acquired chromatograms were normalized. All the compounds exhibited HPLC purity > 95%. The identity of some compounds (**7**, **9**, **11**, **12**, **13**, and **14**) was corroborated through exact mass determination (HR-MS spectra are available in the Supplementary Material file). The exact mass determination (HR-MS) was performed on a Thermo Scientific Exactive spectrometer equipped with an ESI source and an Orbitrap analyzer. Compound samples were prepared by 1/10,000 dilution in methanol and were injected into the HESI source at a flow rate of 10 μL/min. Data were collected using 20/25 scans employing Xcalibur software (Thermo Scientific). The instrument was operated in positive or negative ion mode (for further information see Table S1, Supplementary Material).

#### General Synthetic Procedure and NMR Data for the Compounds **2**–**15**

To a solution of coumalic acid (1.1 eq.) in DMF (4 mL) under nitrogen atmosphere (N_2_) and in an ice bath (4 °C), 2-(1*H*-benzotriazole-1-yl)-1,1,3,3-tetramethyluronium tetrafluoroborate (TBTU, 1.15 eq.) and *N*,*N*-diisopropylethylamine (DIPEA, 5 eq.) were added. The mixture was stirred for 30 min and then the proper amine (1.0 eq.) was added. The reaction was stirred at 4 °C for 24 to 48 h, depending on amine reactivity. Once the reaction was completed (monitored by TLC), the mixture was quenched with 10 mL of a saturated solution of NaHCO_3_ and stirred for 10 min. Afterwards, an additional quantity of NaHCO_3_ saturated solution was added, for a final volume of 200 mL.

The solution obtained was stored at 4 °C for 1 h and successive work-up consisted of filtration or extraction depending on compound behavior. The derivatives producing precipitate were filtered and then the solid was purified by column chromatography on silica gel, using a proper mixture of cyclohexane/ethyl acetate. The compounds that did not precipitate were extracted with dichloromethane (3 × 30 mL), and the organics were reunited and dried over sodium sulfate and filtered to remove the drying agent. The organic phase was evaporated in vacuo and then crude purified with chromatographic procedures on silica gel and using a proper mixture of cyclohexane/ethyl acetate. When it was deemed necessary, the solids obtained were further crystallized from a mixture of dichloromethane/cyclohexane (for further information see Table S2, Supplementary Material).

**2-Oxo-*N*-phenyl-2*H*-pyran-5-carboxamide (2).** Bright yellow solid (yield 68%), mp 225–227 °C. ^1^H NMR (400 MHz, DMSO-*d*_6_): *δ* 5.74 (d, *J* = 9.3 Hz, 1H, CH_Pyr_), 7.23–7.26 (m, 1H, Ar), 7.43–7.47 (m, 2H, Ar), 7.55 (d, *J* = 7.9 Hz, 2H, Ar), 7.65 (d, *J* = 9.4 Hz, 1H, CH_Pyr_), 8.68 (d, *J* = 14.1 Hz, 1H, CH_Pyr_), 11.15 (d, *J* = 14.4 Hz, 1H, NHCO). ^13^C NMR (101 MHz, DMSO-*d*_6_): *δ* 95.8 (C_Pyr_), 103.9 (CH_Pyr_), 119.1 (2 × Ar), 126.4 (Ar), 130.1 (2 × Ar), 139.1 (Ar), 149.1 (CH_Pyr_), 153.2 (CH_Pyr_), 162.0 (CO), 163.9 (NHCO).

**2-Oxo-*N*-(p-tolyl)-2*H*-pyran-5-carboxamide (3).** Yellow solid (yield 30%), mp 226–228 °C. ^1^H NMR (400 MHz, DMSO-*d*_6_): *δ* 2.30 (s, 3H, CH_3_), 5.72 (d, *J* = 9.3 Hz, 1H, CH_Pyr_), 7.25 (d, *J* = 8.1 Hz, 2H, Ar), 7.43 (d, *J* = 8.3 Hz, 2H, Ar), 7.64 (d, *J* = 9.4 Hz, 1H, CH_Pyr_), 8.64 (d, *J* = 14.1 Hz, 1H, CH_Pyr_), 11.11 (d, *J* = 14.4 Hz, 1H, NHCO). ^13^C NMR (101 MHz, DMSO-*d*_6_): *δ* 20.9 (CH_3_), 95.5 (C_Pyr_), 103.5 (CH_Pyr_), 119.1 (2 × Ar), 130.5 (2 × Ar), 135.9 (Ar), 136.6 (Ar), 149.1 (CH_Pyr_), 153.3 (CH_Pyr_), 162.0 (CO), 163.9 (NHCO).

**2-Oxo-*N*-(*m*-tolyl)-2*H*-pyran-5-carboxamide (4).** Yellow solid (yield 7%), mp 180–182 °C. ^1^H NMR (400 MHz, DMSO-*d*_6_): *δ* 2.34 (s, 3H, CH_3_), 5.75 (d, *J* = 9.2 Hz, 1H, CH_Pyr_), 7.07 (t, *J* = 4.4 Hz, 1H, Ar), 7.33 (d, *J* = 4.9 Hz, 2H, Ar), 7.37 (s, 1H, Ar), 7.65 (d, *J* = 9.4 Hz, 1H, CH_Pyr_), 8.68 (d, *J* = 14.2 Hz, 1H, CH_Pyr_), 11.11 (d, *J* = 13.9 Hz, 1H, NHCO). ^13^C NMR (101 MHz, DMSO-*d*_6_): *δ* 21.4 (CH_3_), 95.8 (C_Pyr_), 103.8 (CH_Pyr_), 116.2 (Ar), 119.3 (Ar), 127.1 (Ar), 129.9 (Ar), 138.9 (Ar), 139.7 (Ar), 149.0 (CH_Pyr_), 153.1 (CH_Pyr_), 161.9 (CO), 164.0 (NHCO).

***N*-(4-Isopropylphenyl)-2-oxo-2*H*-pyran-5-carboxamide (5).** Yellow solid (yield 6%), mp 202–204 °C. ^1^H NMR (400 MHz, DMSO-*d*_6_): *δ* 1.21 (d, *J* = 6.9 Hz, 6H, 2 × CH_3_), 2.87–2.94 (m, 1H, CH), 5.73 (d, *J* = 9.3 Hz, 1H, CH_Pyr_), 7.32 (d, *J* = 8.3 Hz, 2H, Ar), 7.46 (d, *J* = 8.3 Hz, 2H, Ar), 7.65 (d, *J* = 9.3 Hz, 1H, CH_Pyr_), 8.64 (d, *J* = 14.0 Hz, 1H, CH_Pyr_), 11.16 (d, *J* = 13.3 Hz, 1H, NHCO). ^13^C NMR (101 MHz, DMSO-*d*_6_): *δ* 24.2 (2 × CH_3_), 33.4 (CH), 95.5 (C_Pyr_), 103.5 (CH_Pyr_), 119.1 (2 × Ar), 127.9 (2 × Ar), 136.9 (Ar), 146.9 (Ar), 149.1 (CH_Pyr_), 153.2 (CH_Pyr_), 162.0 (CO), 163.9 (NHCO).

***N*-(4-Methoxyphenyl)-2-oxo-2*H*-pyran-5-carboxamide (6).** Bright yellow solid (yield 21%), mp 241–243 °C. ^1^H NMR (400 MHz, DMSO-*d*_6_): *δ* 3.77 (s, 3H, CH_3_), 5.70 (d, *J* = 9.3 Hz, 1H, CH_Pyr_), 7.02 (d, *J* = 8.9 Hz, 2H, Ar), 7.50 (d, *J* = 8.9 Hz, 2H, Ar), 7.63 (d, *J* = 9.3 Hz, 1H, CH_Pyr_), 8.58 (d, *J* = 14.3 Hz, 1H, CH_Pyr_), 11.17 (d, *J* = 14.0 Hz, 1H, NHCO). ^13^C NMR (101 MHz, DMSO-*d*_6_): *δ* 55.9 (CH_3_), 95.2 (C_Pyr_), 103.1 (CH_Pyr_), 115.2 (2 × Ar), 120.7 (2 × Ar), 132.3 (Ar), 149.1 (CH_Pyr_), 153.3 (CH_Pyr_), 158.0 (Ar), 162.1 (CO), 163.9 (NHCO).

***N*-(3-Methoxyphenyl)-2-oxo-2*H*-pyran-5-carboxamide (7).** Yellow solid (yield 21%), mp 241–243 °C. ^1^H NMR (600 MHz, DMSO-*d*_6_): *δ* 3.80 (s, 3H, CH_3_), 5.75 (d, *J* = 9.3 Hz, 1H, CH_Pyr_), 6.81-6.83 (m, 1H, Ar), 7.11 (d, *J* = 7.9 Hz, 1H, CH_Pyr_), 7.19 (s, 1H, Ar), 7.35 (t, *J* = 8.1 Hz, 1H, Ar), 7.65 (d, *J* = 9.4 Hz, 1H, CH_Pyr_), 8.68 (d, *J* = 14.1 Hz, 1H, CH_Pyr_), 11.09 (d, *J* = 14.1 Hz, 1H, NHCO). ^13^C NMR (151 MHz, DMSO-*d*_6_): *δ* 55.4 (CH_3_), 95.4 (C_Pyr_), 103.6 (CH_Pyr_), 104.3 (Ar), 110.6 (Ar), 111.7 (Ar), 120.4 (Ar), 130.5 (Ar), 139.8 (Ar), 148.5 (CH_Pyr_), 152.6 (CH_Pyr_), 160.3 (CO), 163.4 (NHCO). HRMS (+) *m/z* 268.0582 [M + Na]^+^ (calcd for C_13_H_11_NO_4_Na, 268.0580; Δm = 0.7).

***N*-(4-Chlorophenyl)-2-oxo-2*H*-pyran-5-carboxamide (8).** Bright yellow solid (yield 25%), mp >250 °C. ^1^H NMR (400 MHz, DMSO-*d*_6_): *δ* 5.76 (d, *J* = 9.3 Hz, 1H, CH_Pyr_), 7.53 (d, *J* = 8.8 Hz, 2H, Ar), 7.63–7.65 (m, 3H, Ar and CH_Pyr_), 8.64 (d, *J* = 14.1 Hz, 1H, CH_Pyr_), 11.13 (d, *J* = 14.2 Hz, 1H, NHCO). ^13^C NMR (101 MHz, DMSO-*d*_6_): *δ* 96.2 (C_Pyr_), 104.3 (CH_Pyr_), 121.0 (2 × Ar), 129.9 (2 × Ar), 130.4 (Ar), 142.7 (Ar), 149.0 (CH_Pyr_), 153.2 (CH_Pyr_), 162.0 (CO), 163.7 (NHCO).

***N*-(4-Bromophenyl)-2-oxo-2*H*-pyran-5-carboxamide (9).** Yellow solid (yield 14%), mp > 250 °C. ^1^H NMR (600 MHz, DMSO-*d*_6_): *δ* 5.77 (d, *J* = 9.4 Hz, 1H, CH_Pyr_), 7.54 (d, *J* = 8.8 Hz, 2H, Ar), 7.65 (dd, *J* = 9.1, 3.2 Hz, 3H, Ar and CH_Pyr_), 8.65 (d, *J* = 14.1 Hz, 1H, CH_Pyr_), 11.13 (d, *J* = 14.1 Hz, 1H, NHCO). ^13^C NMR (101 MHz, DMSO-*d*_6_): *δ* 96.2 (C_Pyr_), 104.3 (CH_Pyr_), 118.5 (Ar), 121.2 (2 × Ar), 132.8 (2 × Ar), 138.6 (Ar), 148.9 (CH_Pyr_), 153.1 (CH_Pyr_), 161.9 (CO), 162.5 (NHCO). HRMS (−) *m/z* 291.9613 [M − H]^−^ (calcd for C_12_H_7_BrNO_3_, 291.9615; Δm = 0.7).

***N-*(2-Iodophenyl)-2-oxo-2*H*-pyran-5-carboxamide (10).** Yellow solid (yield 11%), mp 225–227 °C. ^1^H NMR (400 MHz, DMSO-*d*_6_): *δ* 5.84 (d, *J* = 9.4 Hz, 1H, CH_Pyr_), 7.05 (t, *J* = 7.6 Hz, 1H, Ar), 7.54 (t, *J* = 7.7 Hz, 1H, Ar), 7.66 (dd, *J* = 8.8, 4.4 Hz, 2H, Ar), 7.96 (d, *J* = 7.9 Hz, 1H, CH_Pyr_), 8.79 (d, *J* = 13.5 Hz, 1H, CH_Pyr_), 11.30 (d, *J* = 13.5 Hz, 1H, NHCO). ^13^C NMR (101 MHz, DMSO-*d*_6_): *δ* 90.6 (Ar), 96.7 (C_Pyr_), 105.0 (CH_Pyr_), 117.9 (Ar), 128.0 (Ar), 130.3 (Ar), 139.5 (Ar), 140.2 (Ar), 148.4 (CH_Pyr_), 152.7 (CH_Pyr_), 161.7 (CO), 164.6 (NHCO).

***N*-([1,1′-Biphenyl]-4-yl)-2-oxo-2*H*-pyran-5-carboxamide (11).** Yellow solid (yield 18%), mp 242–244 °C. ^1^H NMR (600 MHz, DMSO-*d*_6_): *δ* 5.76 (d, *J* = 9.3 Hz, 1H, CH_Pyr_), 7.37 (t, *J* = 7.3 Hz, 1H, Ar), 7.47 (t, *J* = 7.7 Hz, 2H, Ar), 7.64–7.71 (m, 5H, Ar, and CH_Pyr_), 7.76 (d, *J* = 8.4 Hz, 2H, Ar), 8.73 (d, *J* = 13.9 Hz, 1H, CH_Pyr_), 11.21 (d, *J* = 14.0 Hz, 1H, NHCO). ^13^C NMR (151 MHz, DMSO-*d*_6_): *δ* 95.6 (C_Pyr_), 103.5 (CH_Pyr_), 119.1 (Ar), 126.5 (2 × Ar), 127.5 (Ar), 127.7 (2 × Ar), 129.0 (2 × Ar), 137.6 (Ar), 138.0 (Ar), 139.0 (Ar), 148.5 (CH_Pyr_), 152.4 (CH_Pyr_), 161.5 (CO), 163.4 (NHCO). HRMS (−) *m/z* 290.0819 [M − H]^−^ (calcd for C_18_H_12_NO_3_, 298.0823; Δm = 1.4).

***N*-(2-Benzylphenyl)-2-oxo-2*H*-pyran-5-carboxamide (12).** Yellow solid (yield 15%), mp 175–177 °C. ^1^H NMR (600 MHz, DMSO-*d*_6_): *δ* 4.09 (s, 2H, CH_2_), 5.74 (d, *J* = 9.3 Hz, 1H, CH_Pyr_), 7.20 (d, *J* = 7.2 Hz, 3H, Ar), 7.26–7.30 (m, 3H, Ar), 7.35 (d, *J* = 7.4 Hz, 1H, Ar), 7.40 (t, *J* = 7.6 Hz, 1H, Ar), 7.57 (d, *J* = 8.0 Hz, 1H, Ar), 7.61 (d, *J* = 9.3 Hz, 1H, Ar), 8.64 (d, *J* = 13.7 Hz, 1H, CH_Pyr_), 11.26 (d, *J* = 13.6 Hz, 1H, NHCO). ^13^C NMR (151 MHz, DMSO-*d*_6_): *δ* 36.3 (CH_2_), 95.6 (C_Pyr_), 103.5 (CH_Pyr_), 118.3 (Ar), 126.4 (Ar), 128.0 (Ar), 128.5 (5 x Ar), 131.2 (Ar), 131.5 (Ar), 136.7 (Ar), 138.5 (Ar) 148.2 (CH_Pyr_), 153.7 (CH_Pyr_), 161.2 (CO), 164.2 (NHCO). HRMS (+) *m/z* 328.0949 [M + Na]^+^ (calcd for C_19_H_15_NO_3_Na, 328.0944; Δm = 1.5).

***N*-(Naphthalen-1-yl)-2-oxo-2*H*-pyran-5-carboxamide (13).** Yellow solid (yield 12%), mp 220–222 °C. ^1^H NMR (600 MHz, DMSO-*d*_6_): *δ* 5.80 (d, *J* = 9.3 Hz, 1H, CH_Pyr_), 7.61–7.74 (m, 5H, Ar), 7.91 (d, *J* = 8.2 Hz, 1H, Ar), 7.97 (d, *J* = 8.4 Hz, 1H, CH_Pyr_), 8.05 (d, *J* = 8.2 Hz, 1H, Ar), 8.84 (d, *J* = 13.4 Hz, 1H, CH_Pyr_), 11.89 (d, *J* = 13.4 Hz, 1H, NHCO). ^13^C NMR (151 MHz, DMSO-*d*_6_): *δ* 96.1 (C_Pyr_), 103.7 (CH_Pyr_), 115.5 (Ar), 120.2 (Ar), 125.0 (Ar), 125.9 (Ar), 126.7 (Ar), 127.0 (Ar), 127.4 (Ar), 128.7 (Ar), 133.7 (Ar), 134.3 (Ar), 148.2 (CH_Pyr_), 154.5 (CH_Pyr_), 161.3 (CO), 164.6 (NHCO). HRMS (+) *m/z* [M + Na]^+^ 288.0637 (calcd for C_16_H_11_NO_3_Na, 288.0631; Δm = 2.1).

***N*-Cyclohexyl-2-oxo-2*H*-pyran-5-carboxamide (14).** Yellow solid (yield 29%), mp 145–148 °C. ^1^H NMR (400 MHz, DMSO-*d*_6_): *δ* 1.08–1.19 (m, 2H, CH_2Chx_), 1.27–1.36 (m, 2H, CH_2Chx_), 1.43-1.64 (m, 2H, CH_2Chx_), 1.69–1.76 (m, *2*H, CH_2Chx_), 1.83-1.94 (m, 2H, CH_2Chx_), 3.44 (dt, *J* = 7.5, 3.8 Hz, 1H, CH_Chx_), 5.51 (d, *J* = 9.2 Hz, 1H, CH_Pyr_), 7.49 (d, *J* = 9.2 Hz, 1H, CH_Pyr_), 8.11 (d, *J* = 14.8 Hz, 1H, CH_Pyr_), 9.71 (dd, *J* = 15.0, 7.5 Hz, 1H, NHCO). ^13^C NMR (151 MHz, DMSO-*d*_6_): *δ* 24.2 (2 × CH_2Chx_), 24.5 (CH_2Chx_), 32.2 (2 × CH_2Chx_), 58.0 (CH_2Chx_), 92.4 (C_Pyr_), 100.4 (CH_Pyr_), 149.1 (CH_Pyr_), 158.2 (CH_Pyr_), 162.0 (CO), 163.7 (NHCO). HRMS (+) *m/z* [M + Na]^+^ 244.0945 (calcd for C_12_H_15_NO_3_Na, 244.0944; Δm = 0.4).

***N*-(3-Morpholinopropyl)-2-oxo-2*H*-pyran-5-carboxamide (15).** Yellow solid (yield 12%), mp 131–133 °C. ^1^H NMR (400 MHz, DMSO-*d*_6_): *δ* 1.73–1.79 (m, 2H, CH_2Morph_), 2.33 (t, *J* = 6.2 Hz, 6H, 3 × CH_2_), 3.50 (q, *J* = 6.4 Hz, 2H, CH_2Morph_), 3.58–3.60 (m, 4H, 2 × CH_2Morph_), 5.49 (d, *J* = 9.2 Hz, 1H, CH_Pyr_), 7.47 (d, *J* = 9.2 Hz, 1H, CH_Pyr_), 8.01 (d, *J* = 14.9 Hz, 1H, CH_Pyr_), 9.88 (d, *J* = 14.5 Hz, 1H, NHCO). ^13^C NMR (101 MHz, DMSO-*d*_6_): *δ* 26.2 (CH_2_), 49.4 (CH_2_), 53.7 (2 × CH_2Morph_), 56.1 (CH_2_), 66.3 (2 × CH_2Morph_), 92.8 (C_Pyr_), 100.6 (CH_Pyr_), 149.7 (CH_Pyr_), 160.6 (CH_Pyr_), 162.7 (CO), 163.9 (NHCO).

### 3.2. Biological Evaluation

#### 3.2.1. Carbonic Anhydrase Inhibition Screening Assay

An Applied Photophysics stopped-flow instrument was used to assay the CA-catalyzed CO_2_ hydration activity [49]. Phenol red (at a concentration of 0.2 mM) was used as an indicator, working at the absorbance maximum of 557 nm, with 20 mM Hepes (pH 7.4) as a buffer and 20 mM Na_2_SO_4_ (to maintain constant ionic strength), following the initial rates of the CA-catalyzed CO_2_ hydration reaction for a period of 10–100 s. The CO_2_ concentrations ranged from 1.7 to 17 mM for the determination of the kinetic parameters and inhibition constant [16]. Enzyme concentrations ranged between 5–12 nM. For each inhibitor, at least six traces of the initial 5–10% of the reaction were used to determine the initial velocity. The uncatalyzed rates were determined in the same manner and subtracted from the total observed rates. Stock solutions of the inhibitor (0.1 mM) were prepared in distilled–deionized water and dilutions up to 0.01 nM were conducted thereafter with the assay buffer. Inhibitor and enzyme solutions were preincubated together for 15 min at room temperature prior to the assay, to allow for the formation of the E–I complex. The inhibition constants were obtained by non-linear least-squares methods using PRISM 3 and the Cheng–Prusoff equation as reported earlier and represent the mean from at least three different determinations. All CA isoforms were recombinant proteins obtained in house, as reported earlier [50,51,52].

#### 3.2.2. Antiviability Effect against Breast Adenocarcinoma Cell Line (MCF7)

Human breast adenocarcinoma cell line, namely MCF7 (ATCC^®^ HTB-22TM), at passages 10–20, was cultured in DMEM high glucose with 10% of fetal bovine serum (FBS), 1% of penicillin/streptomycin, and 1% of l-glutamine (all purchased by EuroClone, Milan, Italy). Cell culture was kept at 37 °C in a humidified atmosphere with 5% CO_2_.

#### 3.2.3. Hypoxia Induction and MCF7 Treatment

MCF7 cells were seeded at density of 10,000/well into a 96-well culture plate and cultured for 24 h. After 24 h, the medium was replaced with a fresh one containing CoCl_2_ 100 µM in order to induce hypoxia, as already reported elsewhere [23]. CoCl_2_ treatment was kept for 48 h, then the same cells were treated with doxorubicin alone and in combination with compounds **1**, **11**, and **14** previously solubilized in DMSO (DMSO final concentration established at 0.1% in all tested samples) for 72 h. Doxorubicin was administered at 2.5 and 1.25 µM concentrations, whereas the newly synthetized molecules were all administered at 100 µM. The metabolic activity was evaluated through MTT test after 72 h of treatment.

#### 3.2.4. HGF Culture and Treatment

Ten healthy donors, before undergoing third molar surgical extraction, signed the informed consent according to the Italian Law and the Ethical Principles for Medical Research code including Human Subjects of the World Medical Association (Declaration of Helsinki). The project received the approval of the Local Ethical Committee of the University of Chieti (Chieti, Italy; approval number 1173, approved on 31 March 2016). Gingiva fragments were rinsed in phosphate-buffered saline (PBS), placed in DMEM medium, cut into small pieces, and cultured by adding the medium with 10% fetal bovine serum (FBS), 1% penicillin/streptomycin, and 1% fungizone (all purchased from Merck Life Science, Milan, Italy). After 10 days, fungizone was removed and cells cultured until 5–8 passages, as already reported elsewhere [53]. Cell culture was kept at 37 °C in humidified atmosphere with 5% CO_2_.

HGFs were seeded at a density of 6700/well into a 96-well culture plate and cultured for 24 h. After 24 h, the medium was replaced with a fresh one containing compounds **1**, **11**, and **14,** previously solubilized in DMSO (DMSO final concentration established at 0.15% in all tested samples) for 48 h. Compounds **1**, **11**, and **14** were all administered at doses ranging from 200 to 1.56 µM. After 48 h of treatment, cells were processed for MTT and LDH analyses.

#### 3.2.5. MTT Assay

MCF7 and HGF metabolic activity was measured through the MTT (3-(4,5-dimethylthiazol-2-yl)-2,5-diphenyltetrazolium bromide) (Sigma Aldrich, Milan, Italy) test. For MCF7, the MTT test was performed after 72 h of treatment with doxorubicin (2.5 µM and 1.25 µM) alone and in combination with compounds **1**, **11**, and **14**. For HGFs MTT test was carried out after 48 h of treatment with compounds **1**, **11**, and **14** at doses ranging from 200 µM to 1.56 µM. Briefly, the medium was replaced with a fresh one supplemented with 0.5 mg/mL MTT and probed for 4 h at 37 °C. After that, the medium containing MTT was discarded and replaced by an equal volume of DMSO to dissolve formazan crystals. The absorbance of the solution was read at 570 nm by means of a microplate reader (Multiskan GO, Thermo Scientific, Waltham, MA, USA). Values obtained without cells were considered as background. Viability was normalized to control cells treated with DMSO 0.1% and CoCl_2_ 100 µM.

#### 3.2.6. Cytotoxicity Assay (LDH Test)

To evaluate HGF cytotoxicity after 48 h of treatment with compounds **1**, **11**, and **14** 200 µM, LDH leakage into the culture medium was measured through CytoTox 96 non-radioactive cytotoxicity assay (Promega, Madison, WI, USA), following the manufacturer’s instructions. The assay was performed in triplicate and in each well the measured LDH leakage in the supernatant was normalized with the LDH leakage optical density values obtained from lysis. Final values were expressed as percentages.

#### 3.2.7. Statistical Analysis

Statistical analysis was performed with GraphPad 8 software by means of Ordinary One-Way ANOVA, followed by Tukey’s post hoc multiple comparisons test.

### 3.3. Molecular Modeling Protocols

#### 3.3.1. Molecular Docking

The crystallographic structure of hCA IX in complex with the reference inhibitor AAZ at 2.2 Å resolution (PDB-ID: 3IAI, chain A) was used as rigid receptor [48]. Sequence analysis revealed the C41S single point mutation in the crystallographic structure compared to the hCA IX sequence deposited in the UniProt database (Q16790) [54]. Since this mutation is at a distance higher than 20 Å from the catalytic site, it was not accounted for in molecular docking simulations. Small molecules were sketched in PICTO (OpenEye Scientific Software, Santa Fe, NM, USA) [55] and converted into a 3D format by OMEGA (OpenEye Scientific Software) [56,57]. Ligand protonation state was assigned by QUACPAC (OpenEye Scientific Software) [58], while energy minimization was carried out by SZYBKI (OpenEye Scientific Software) using the MMFF94S force field [59]. Molecular docking was carried out with GOLD (The Cambridge Crystallographic Data Centre, Cambridge, UK) version 2020.1 [60,61], using the ChemPLP fitness function for docking and scoring purposes. Based on structural and literature evidence, the binding site for docking simulations was centered on the catalytic Zn(II) ion (coordinates 67.419, 55.704, 14.134 in the crystallographic structure), and it was assigned with a radius of 14 Å. The Zn(II)-coordinated water molecule was modeled as described previously [40], and it was kept as fixed in molecular docking (toggle “on” option). The receptor was prepared with the GOLD docking wizard implemented in Hermes visualizer, which is an integral part of the GOLD interface. Ten runs for each ligand were stored and submitted to visual inspection.

#### 3.3.2. Molecular Dynamics Simulations

MD simulations were run with the AMBER18 program [62]. The ff14SB force field was used to parametrize the protein and the GAFF force field was used to parametrize the ligands, while TIP3P-type water molecules and counter-ions were added to a rectangular box for solvating each docking complex with a buffer of 6 Å from the molecular system, and neutralizing the total charge, respectively. An arbitrary charge of +1 was assigned to the catalytic Zn(II) ion [46], and it was bound to His94, His96, and His119 adopting force field parameters validated previously [63,64,65]. MD simulations were run according to the protocol described earlier, using a time-step of 2 fs [66,67,68]. Briefly, for each system, the solvent was first energy minimized for 500 steps using the steepest descent algorithm (SD), followed by 2500 steps with the conjugate gradient algorithm (CG) while keeping the solute frozen. The solvated solute was then energy minimized for 1000 steps with the SD and 5000 subsequent steps with the CG before being heated to 300 K for 1 ns using the Langevin thermostat. The system’s density was equilibrated for 1 ns by the Berendsen barostat, then a preliminary equilibration of 50 ns was run before the final production of MD trajectories lasting 500 ns. No positional restraints were applied. MD trajectories were analyzed and clustered by the CPPTRAJ software [69]

## 4. Conclusions

The scope of this research was to expand the knowledge regarding hCA inhibition through the design of a novel scaffold based on the well-known coumarin core, modified following an approach similar to that reported by Sapi et al. for saccharin [39]. The majority of these compounds were potent and selective inhibitors of the two tumor-related isoforms (hCA IX and XII). In particular, three of the most potent hCA IX and XII inhibitors (**1**, **11**, and **14**) were assessed against MCF7 breast adenocarcinoma cells, previously treated with CoCl_2_ for 48 h to induce hypoxia. Interestingly, the combination of compound **14** with doxorubicin (2.5 µM) elicited statistically significant viability reduction with respect to administration of doxorubicin alone. This result is further supported by the capability of compound **14** to preserve a very high rate of healthy cell (HGF) viability, also excluding cytotoxic effects. This biological effect further corroborates that these agents are likely to be used in combination with chemotherapy and immunotherapy to treat hypoxic tumors.

Moreover, molecular docking as well as MD simulations shed light on the binding mode of these compounds inside the hCA IX active site, thus unravelling the molecular attributes responsible for the activity. Overall, these in vitro activity data are encouraging for further future development of this scaffold. Recently, selective CAIs were shown to sensitize tumor cells to cytotoxic agents in different preclinical models [70]. This enhanced efficacy can be the result of targeting residual cancer cells that are pharmacologically resistant due to their presence in the hypoxic niche or of suppressing tumor cell migration and invasion.

## Figures and Tables

**Table 1:** Inhibition data against selected human CA isoforms (hCA I, II, IX, and XII) with the novel compounds **1**–**15** and the standard sulfonamide inhibitor acetazolamide (AAZ) by a stopped flow CO_2_ hydrase assay.

Entry	Structure	*K*_I_ (µM) ^a^			
		hCA I	hCA II	hCA IX	hCA XII
**1**	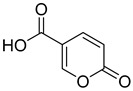	>100	>100	0.073	0.083
**2**	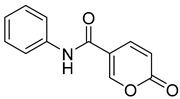	>100	>100	3.000	0.700
**3**	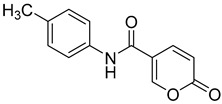	>100	>100	2.900	0.800
**4**	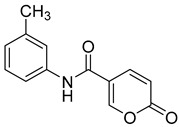	>100	>100	0.174	0.090
**5**	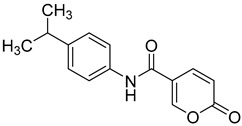	>100	>100	0.273	0.068
**6**	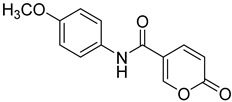	>100	>100	3.100	0.700
**7**	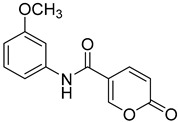	>100	>100	0.315	0.265
**8**	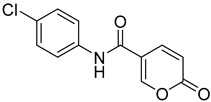	>100	>100	3.200	0.700
**9**	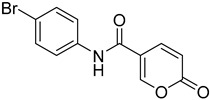	>100	>100	0.082	0.089
**10**	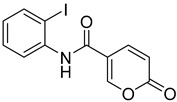	>100	>100	0.281	0.081
**11**	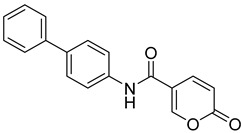	>100	>100	0.083	0.076
**12**	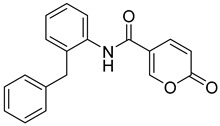	>100	>100	0.146	0.080
**13**	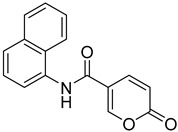	>100	>100	3.689	0.094
**14**	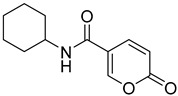	>100	>100	0.098	0.086
**15**	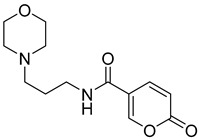	>100	>100	0.326	0.087
**AAZ**		0.20	0.012	0.025	0.006

^a^ Errors are in the range of ±5% of the reported values, from three different assays.

## Data Availability

Data are contained within the article.

## Supplementary Materials

The following Supplementary Materials can be downloaded at: https://www.mdpi.com/article/10.3390/ijms23147950/s1.
