# Supplementary material for: Novel Insights on Human Carbonic Anhydrase Inhibitors Based on Coumalic Acid: Design, Synthesis, Molecular Modeling Investigation, and Biological Studies
Source: Int J Mol Sci. 2022 Jul 19;23(14):7950. doi: 10.3390/ijms23147950 (PMC9324074; doi:10.3390/ijms23147950)

Novel Insights on Human Carbonic Anhydrase Inhibitors Based on Coumalic Acid: Design, Synthesis, Molecular Modeling Investigation, and Biological Studies

Virginia Pontecorvi [1], Mattia Mori [2], Francesca Picarazzi [2], Susi Zara [3], Simone Carradori [3], Amelia Cataldi [3], Andrea Angeli [4,5], Emanuela Berrino [1], Paola Chimenti [1], Alessia Ciogli [1], Daniela Secci [1], Paolo Guglielmi [1,*], Claudiu T. Supuran [4,*]

*Corresponding authors: Paolo Guglielmi, Department of Drug Chemistry and Technologies, Sapienza University of Rome, P.le A. Moro 5, 00185 Rome, Italy. E-mail: paolo.guglielmi@uniroma1.it.
Claudiu T. Supuran, NEUROFARBA Department, Pharmaceutical and Nutraceutical Section, University of Florence, via Ugo Schiff 6, 50019 Sesto Fiorentino, FI, Italy. E-mail: claudiu.supuran@unifi.it.

[1] Department of Drug Chemistry and Technologies, Sapienza University of Rome, P.le A. Moro 5, 00185 Rome, Italy; virginia.pontecorvi@uniroma1.it (V.P.); emanuela.berrino@uniroma1.it (E.B.); paola.chimenti@uniroma1.it (P.C.); alessia.ciogli@uniroma1.it (A.C.); daniela.secci@uniroma1.it (D.S.); paolo.guglielmi@uniroma1.it (P.G.);
[2] Department of Biotechnology, Chemistry and Pharmacy, University of Siena, Via Aldo Moro 2, 53100 Siena, Italy; mattia.mori@unisi.it (M.M.); francesc.picarazzi@student.unisi.it (F.P.)
[3] Department of Pharmacy, "G. d'Annunzio" University of Chieti-Pescara, Via dei Vestini 31, 66100 Chieti, Italy; susi.zara@unich.it (S.Z.); simone.carradori@unich.it (S.C.); amelia.cataldi@unich.it (A.C.);
[4] NEUROFARBA Department, Pharmaceutical and Nutraceutical Section, University of Florence, via Ugo Schiff 6, 50019 Sesto Fiorentino, FI, Italy; andrea.angeli@unifi.it (A.A.); claudiu.supuran@unifi.it (C.T.S.)
[5] Department of Food and Drug, University of Parma, Parco Area delle Scienze, 27/A, 43124 Parma, Italy; andrea.angeli@unifi.it (A.A.);

* Correspondence: paolo.guglielmi@uniroma1.it (P.G.); claudiu.supuran@unifi.it (C.T.S.);

Table of Contents

Figure S1. Overimposition between hCA IX and acetazolamide complexes. (S3)

NMR spectra of compounds **2-15** (S4–S39)

Figure S2. HPLC chromatograms for purity determination (S40)

Enzyme inhibition curves of compounds **2-15** (S41–S43)

Table S1. Instrument parameters for exact mass determination (S44)

Table S2. Purification conditions for compounds **2-15** (S45–S46)

High resolution mass analysis of compounds **7, 9, 11, 12, 13, 14** (S47–S52)

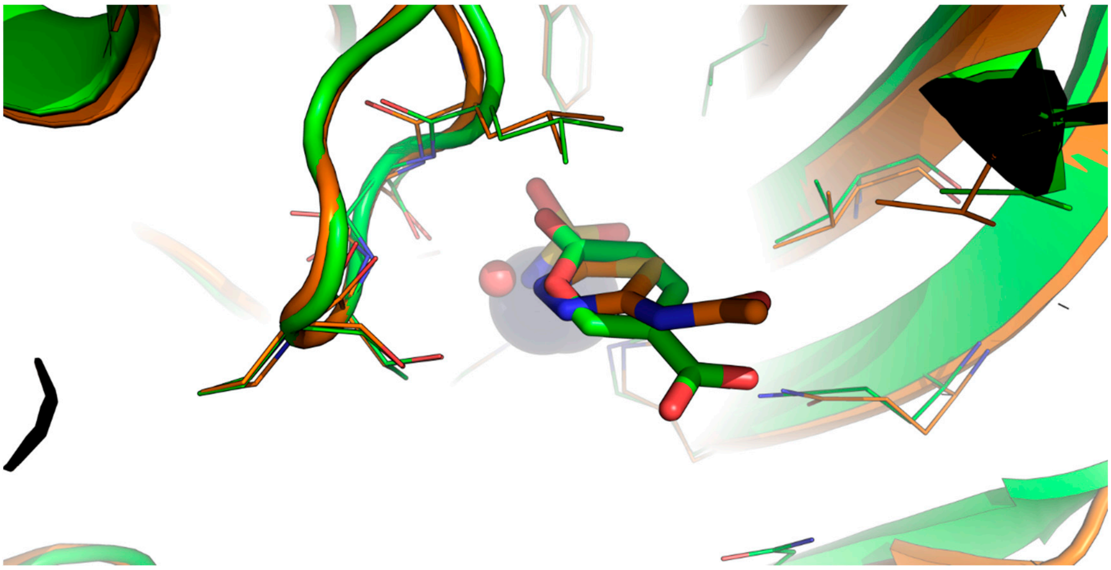

Figure S1. Overimposition between the crystallographic complex between hCA IX and acetazolamide (coloured orange) and the MD-representative structure of the complex between **1** (in its closed form) and hCA IX, which is coloured green. Residues within 4 Å from the ligands are shown as lines. The Zn(II)-bound water molecule in the MD structure is shown as a small red sphere, the catalytic Zn(II) ion is shown as a grey sphere in both structures.

^{1}H NMR spectrum of compound **2**

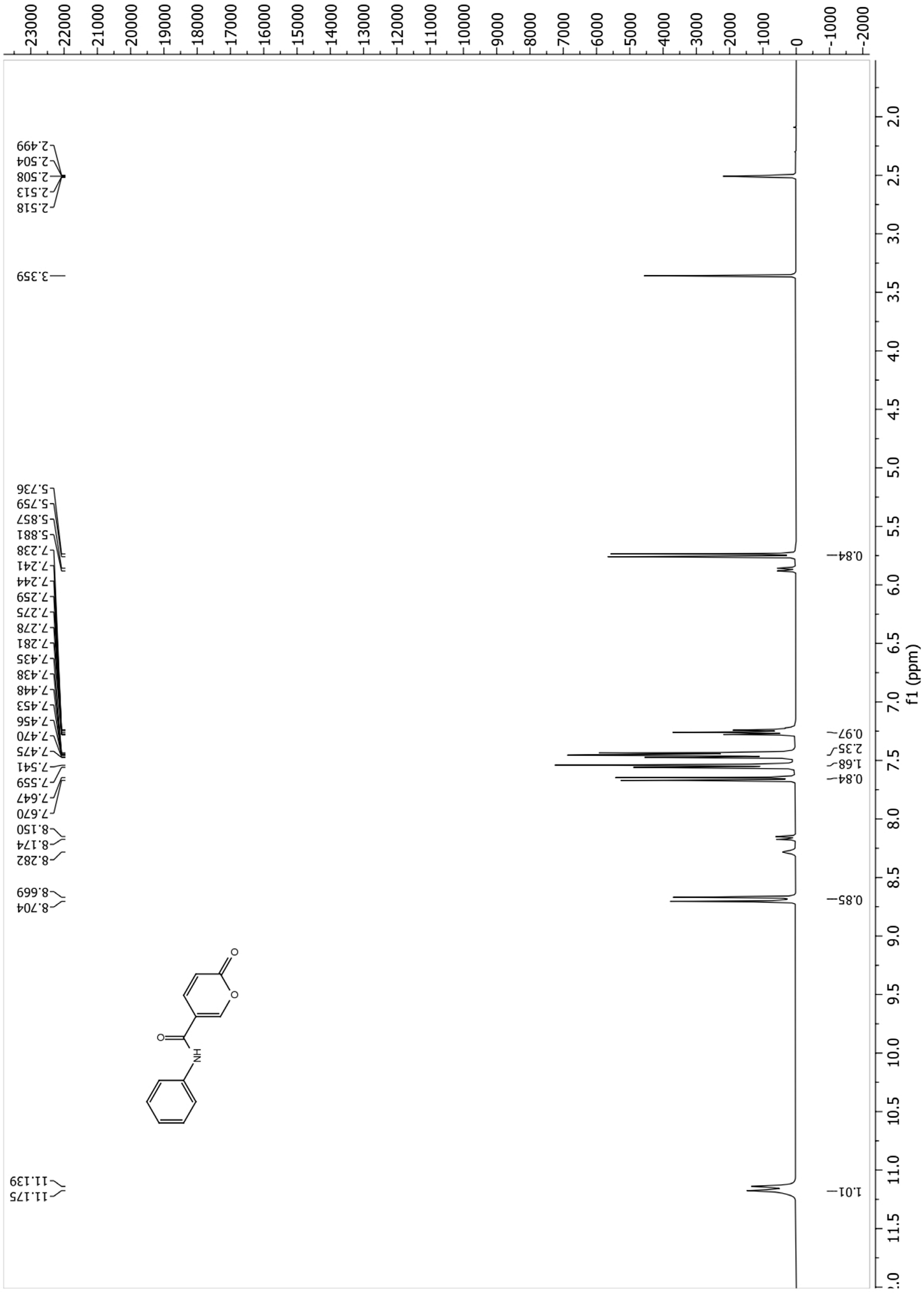

^{13}C NMR spectrum of compound **2**

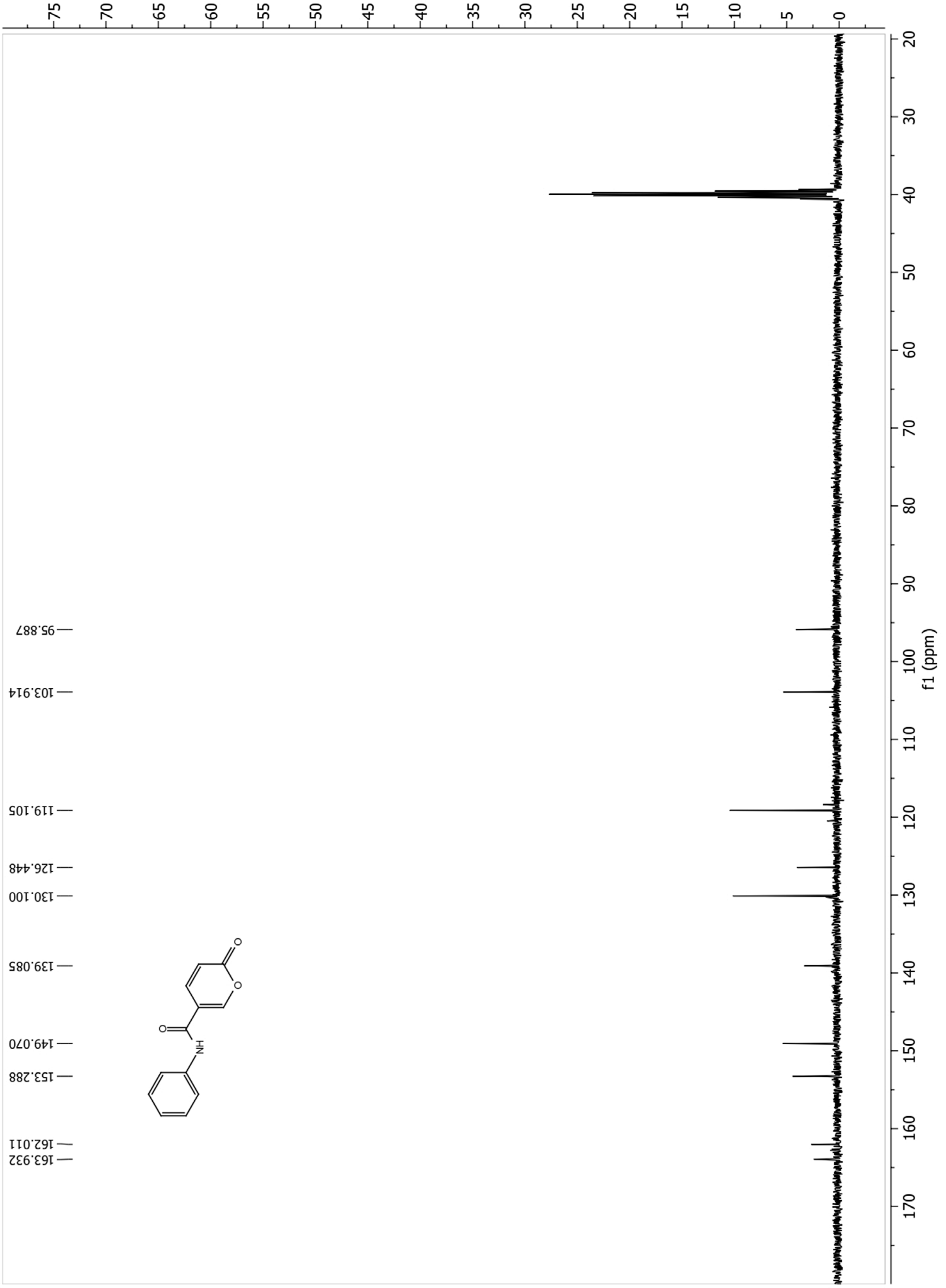

^{1}H NMR spectrum of compound **3**

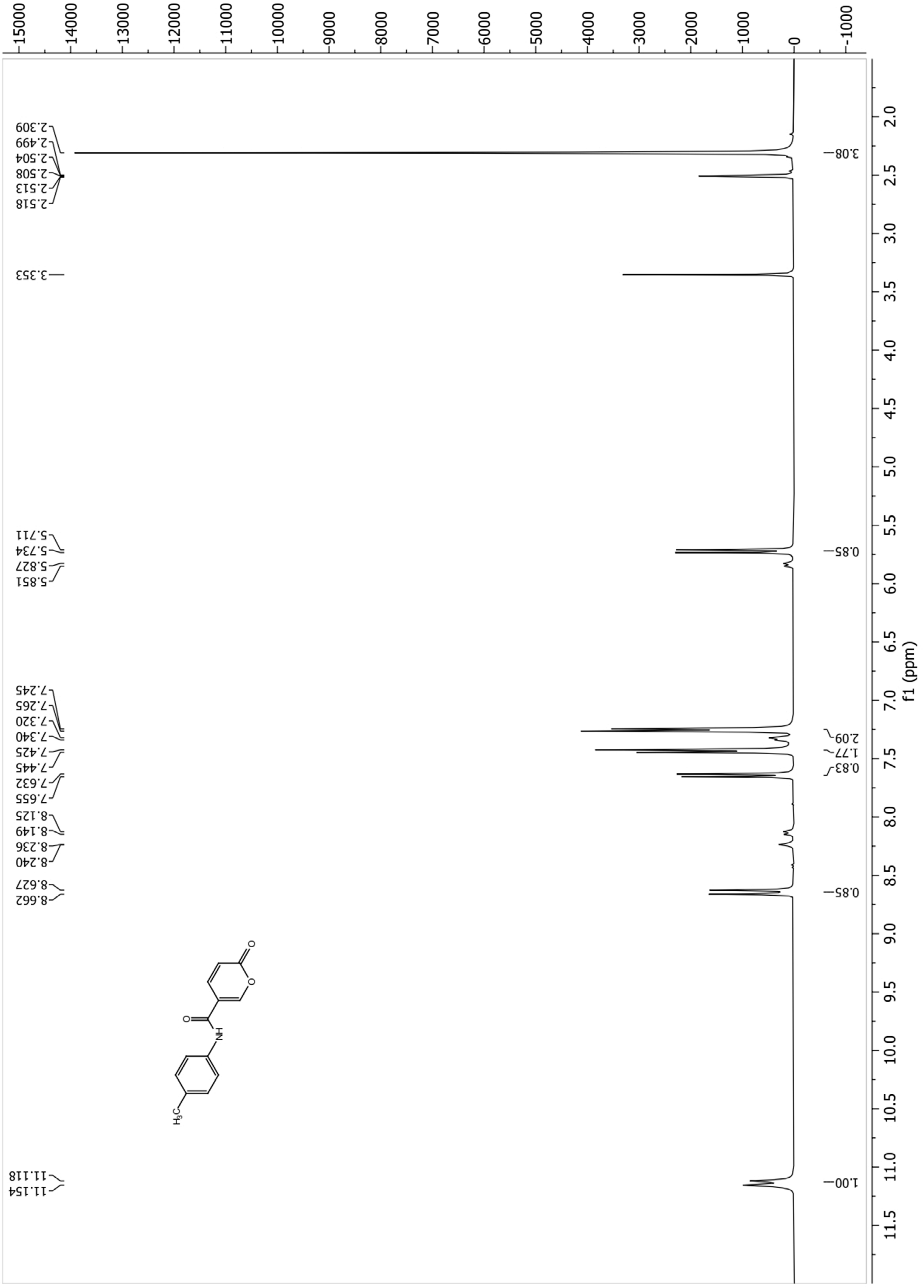

^{13}C NMR spectrum of compound **3**

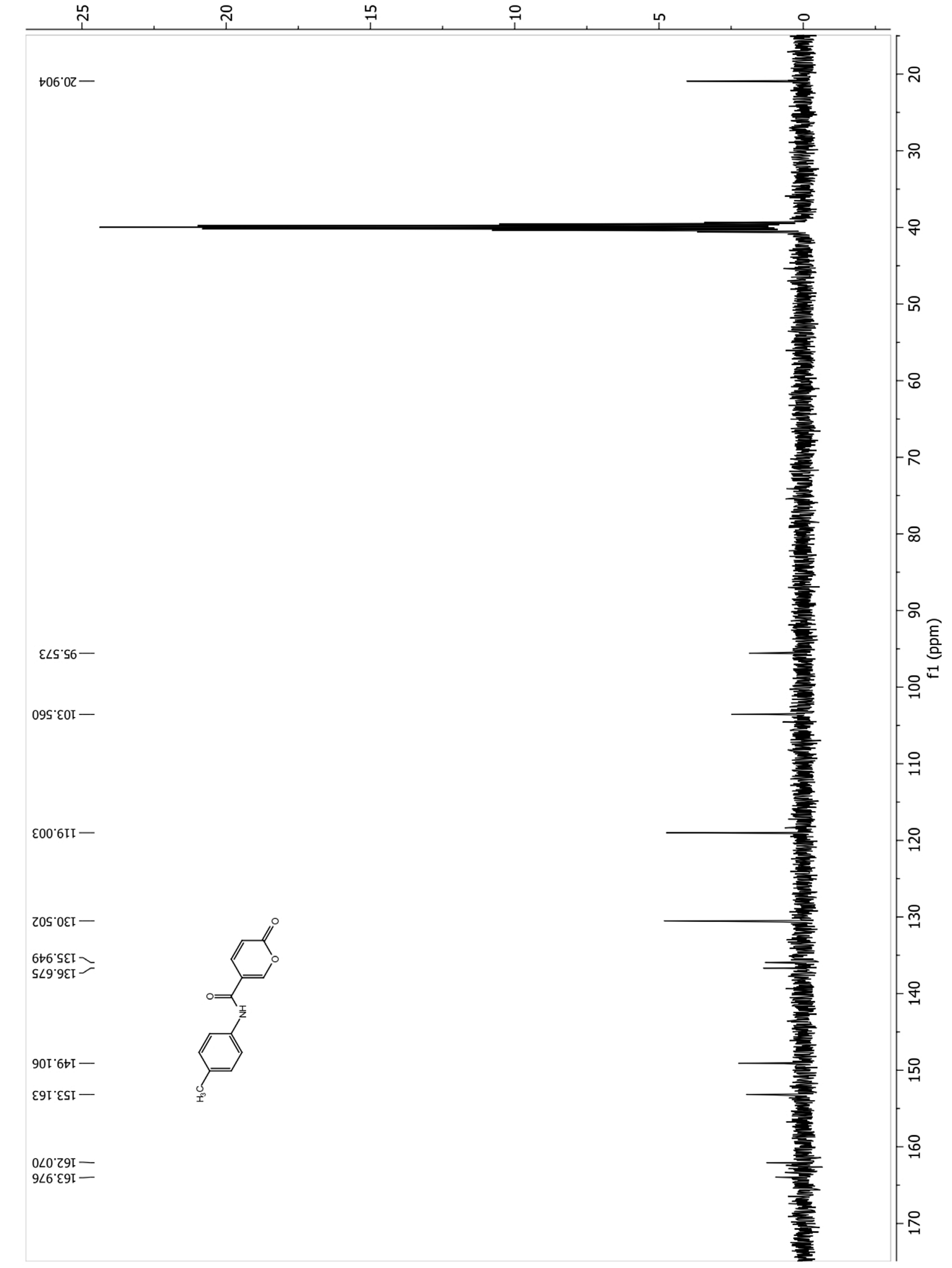

^{1}H NMR spectrum of compound **4**

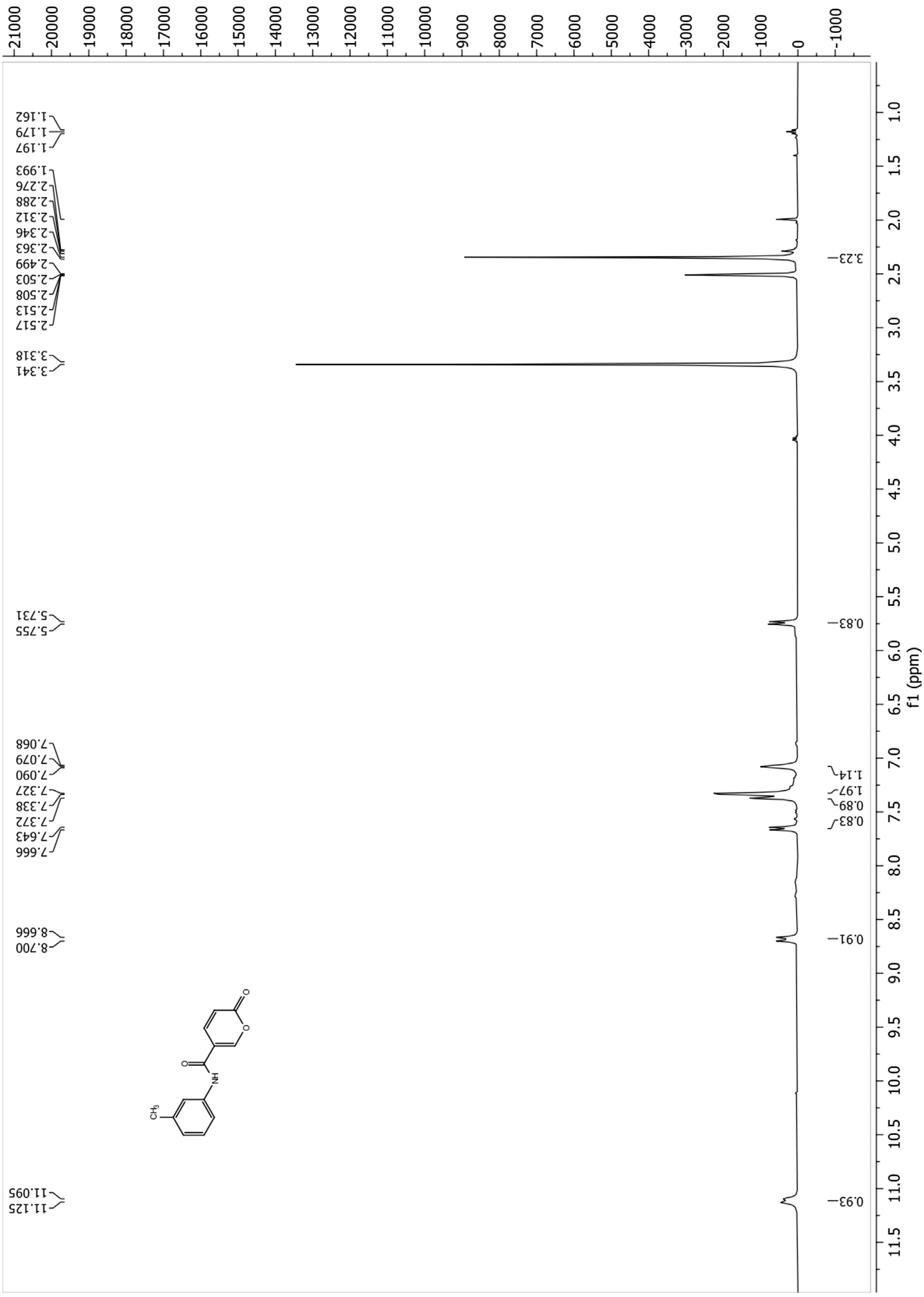

^{13}C NMR spectrum of compound **4**

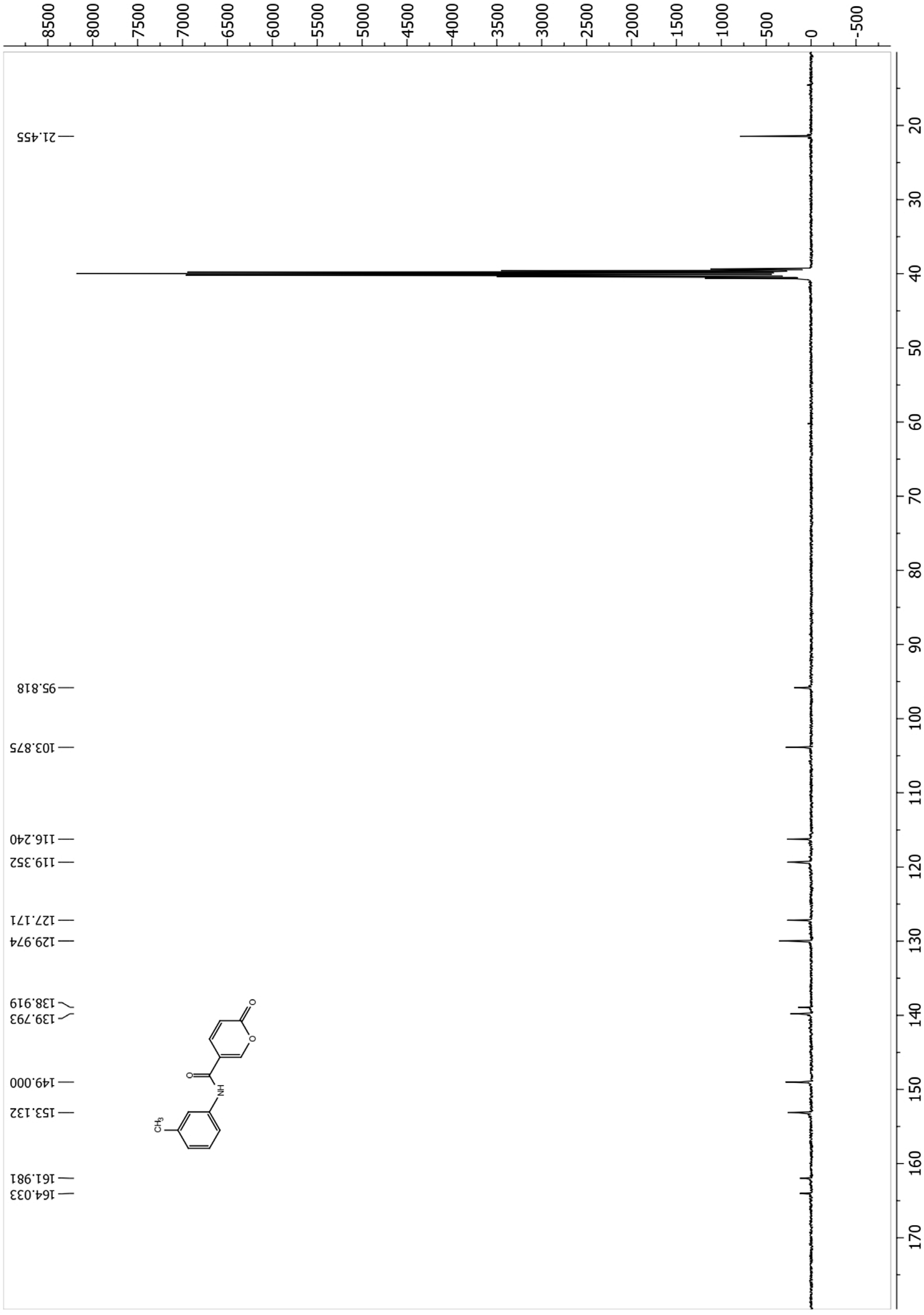

^{1}H NMR spectrum of compound **5**

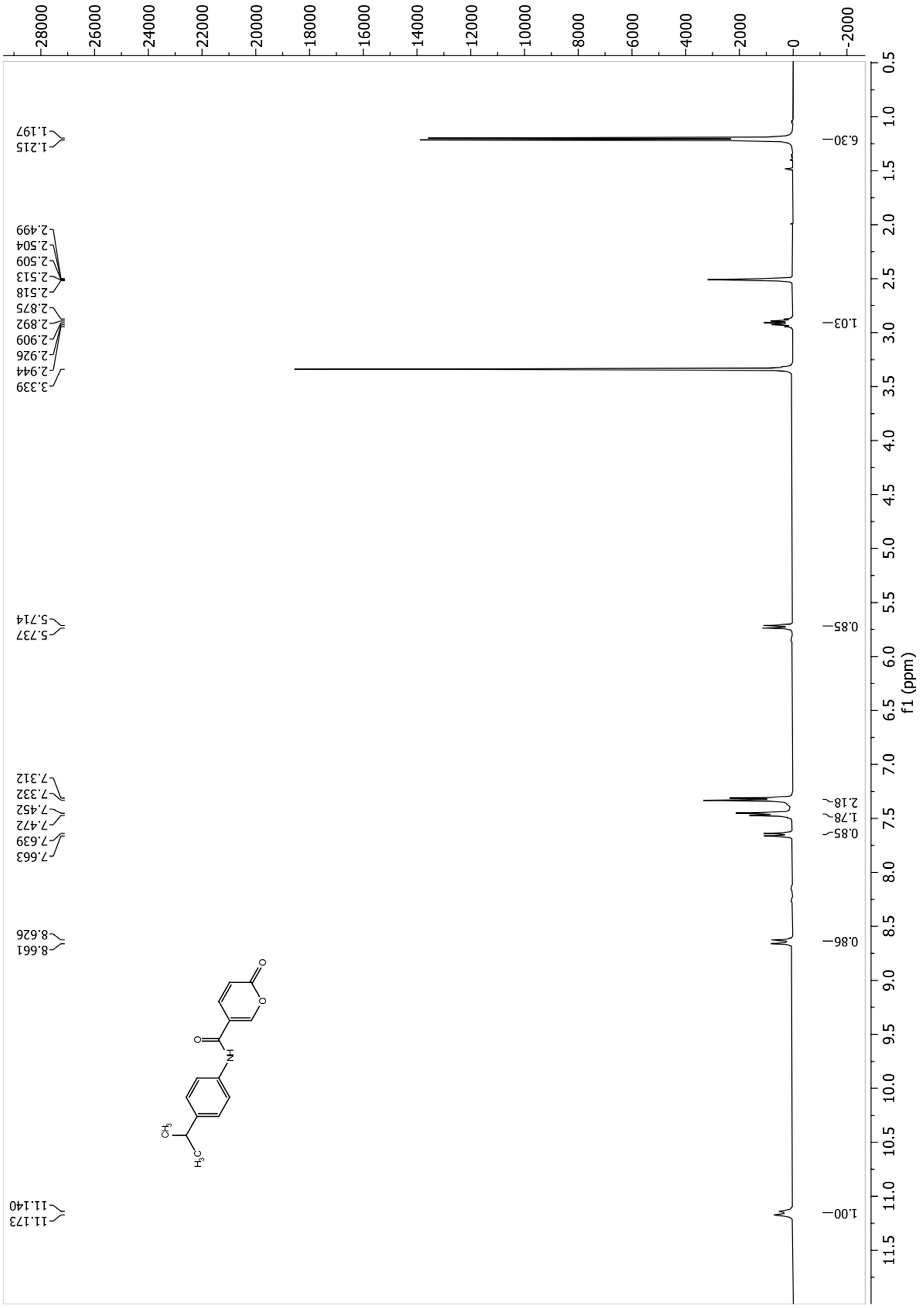

^{1}H-^{1}H NMR-COSY spectrum of compound **5**

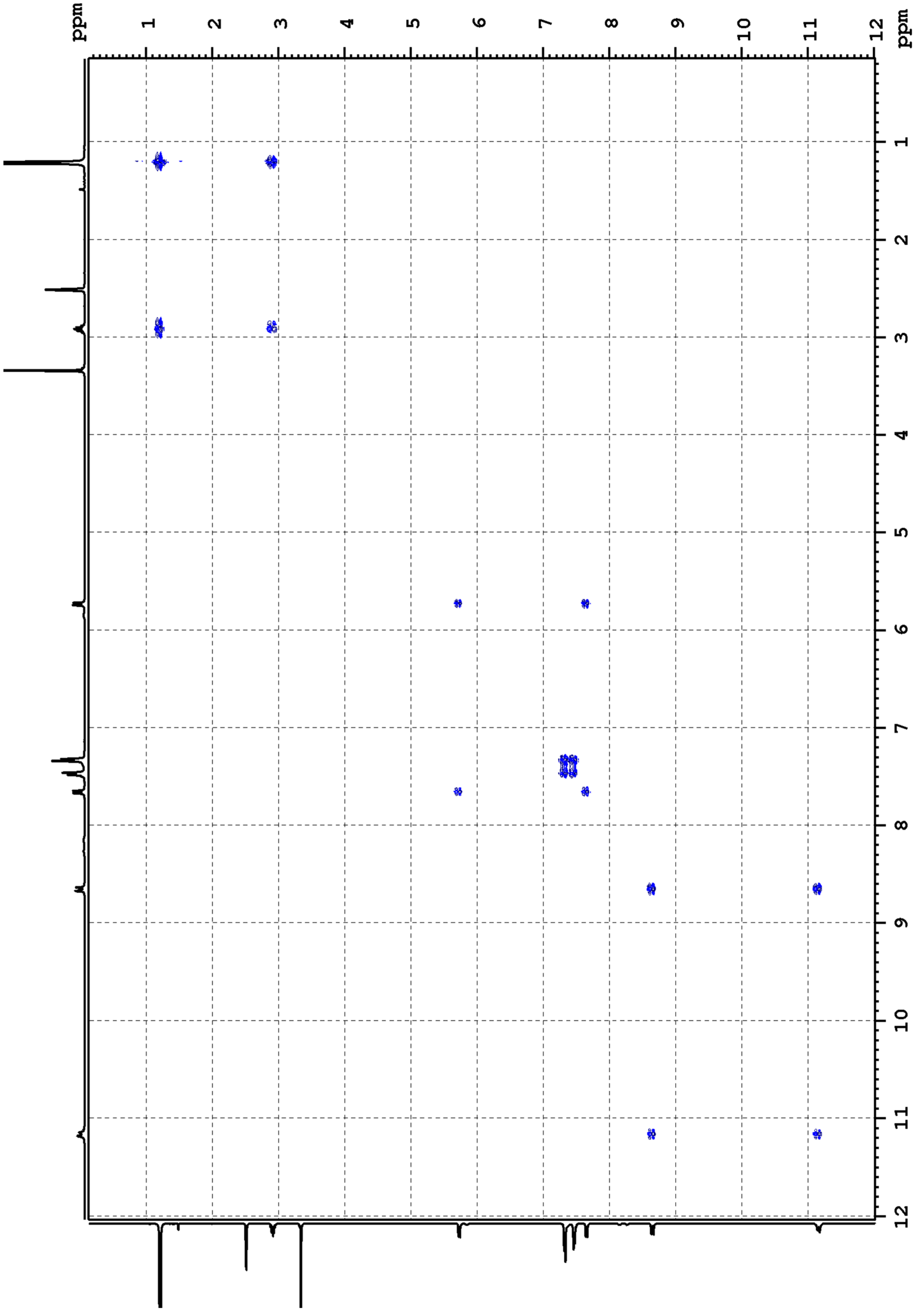

^{1}H-^{1}H NMR-NOESY spectrum of compound **5**

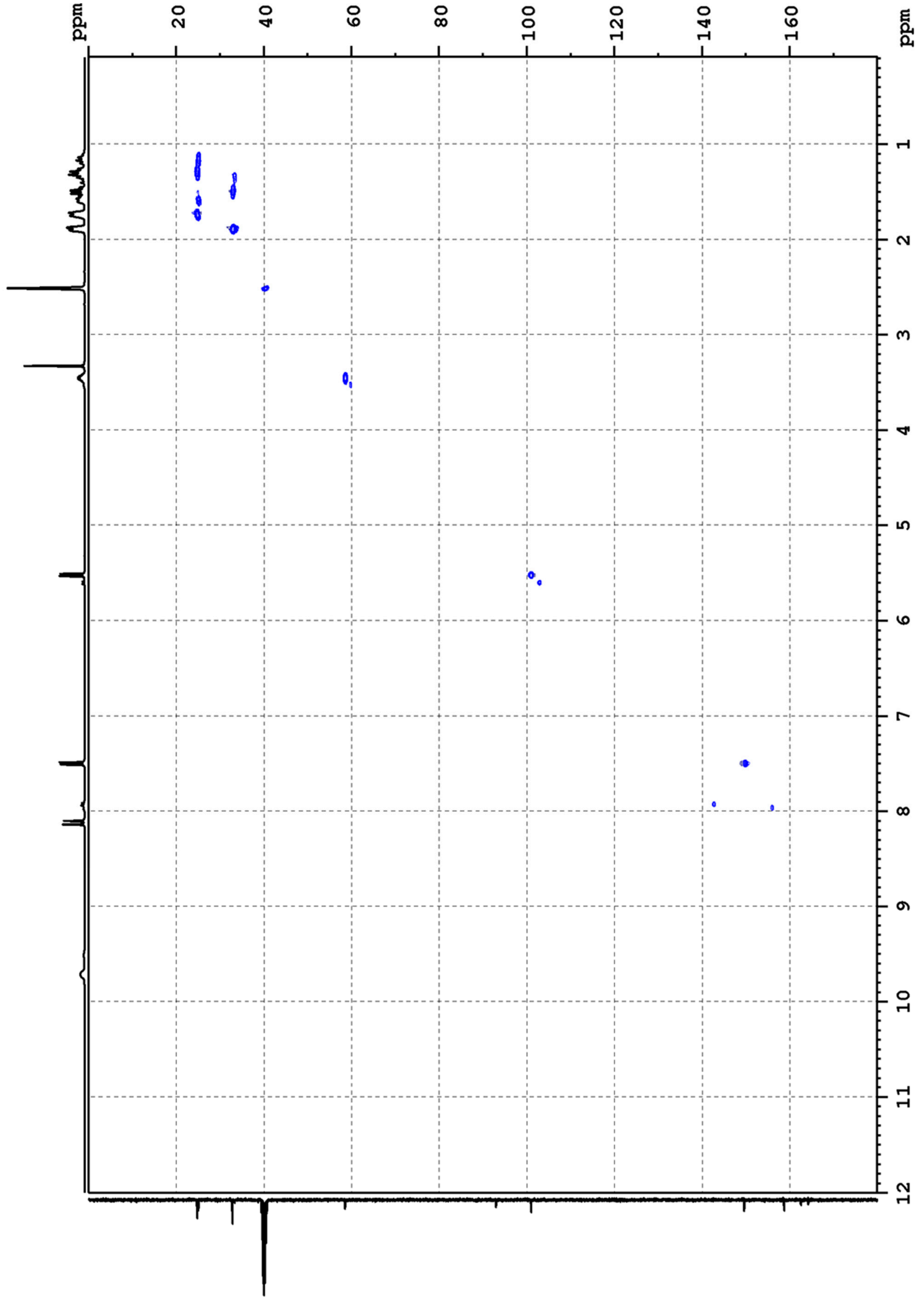

^{13}C NMR spectrum of compound **5**

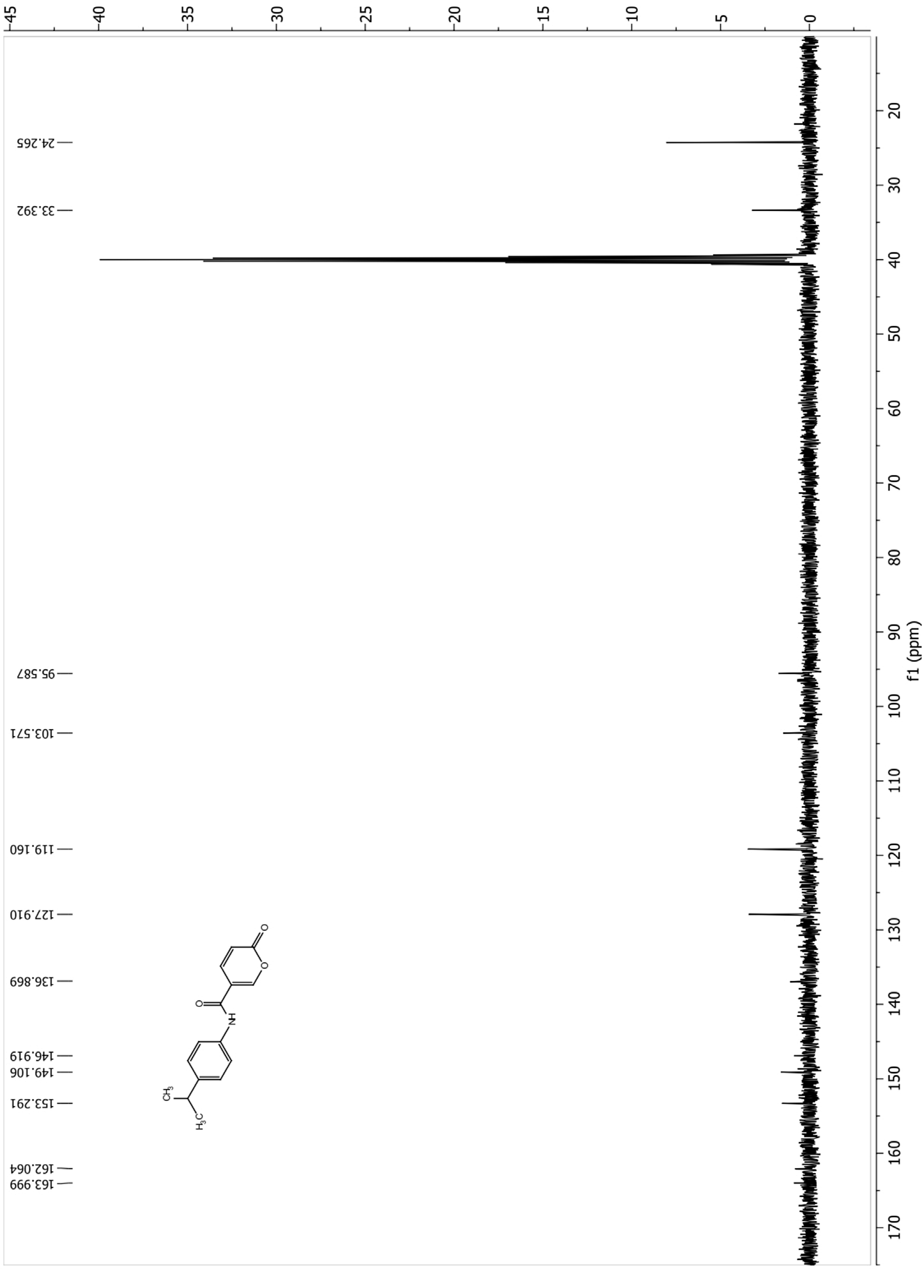

^{1}H–^{13}C HSQC spectrum of compound **5**

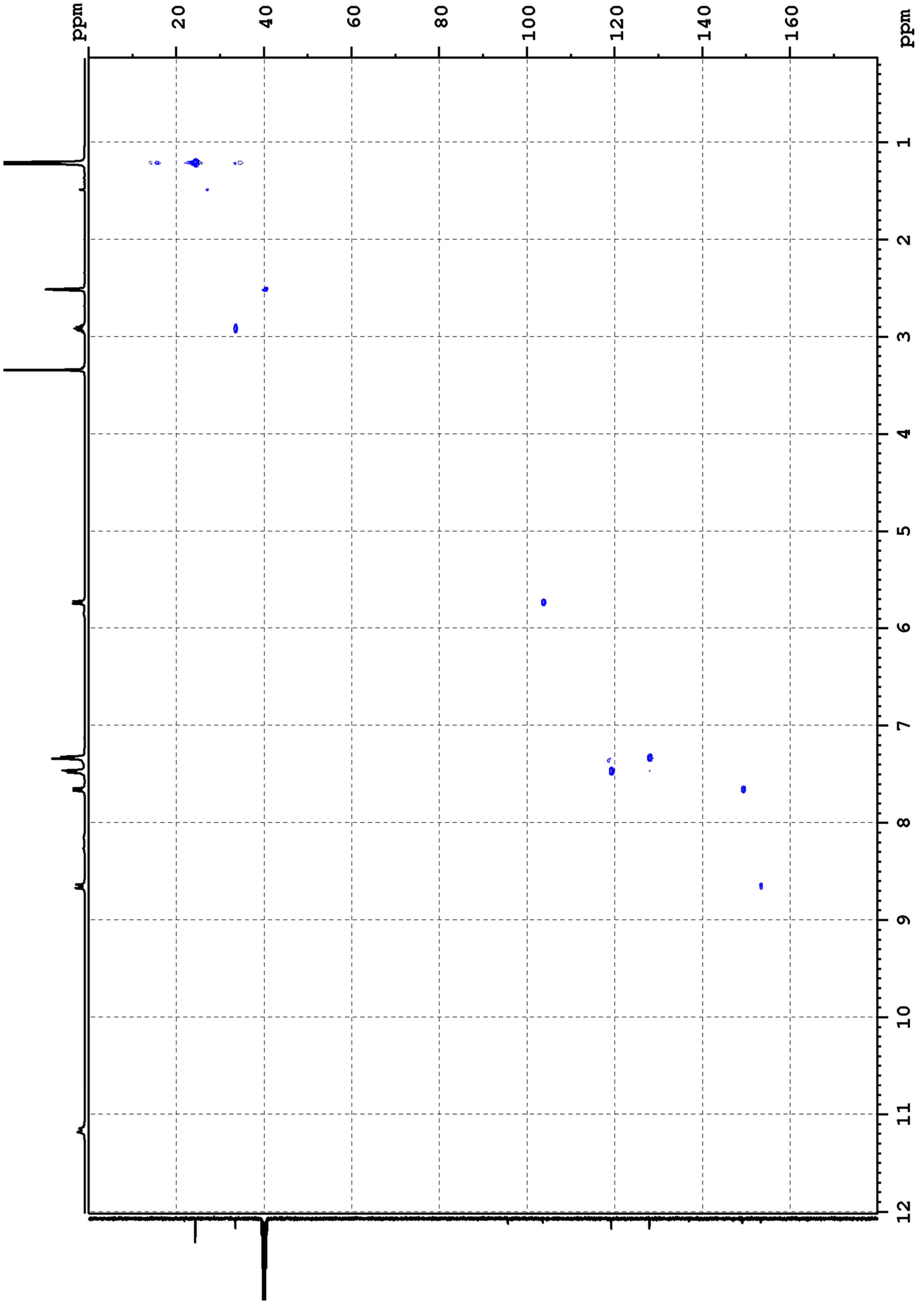

^{1}H NMR spectrum of compound **6**

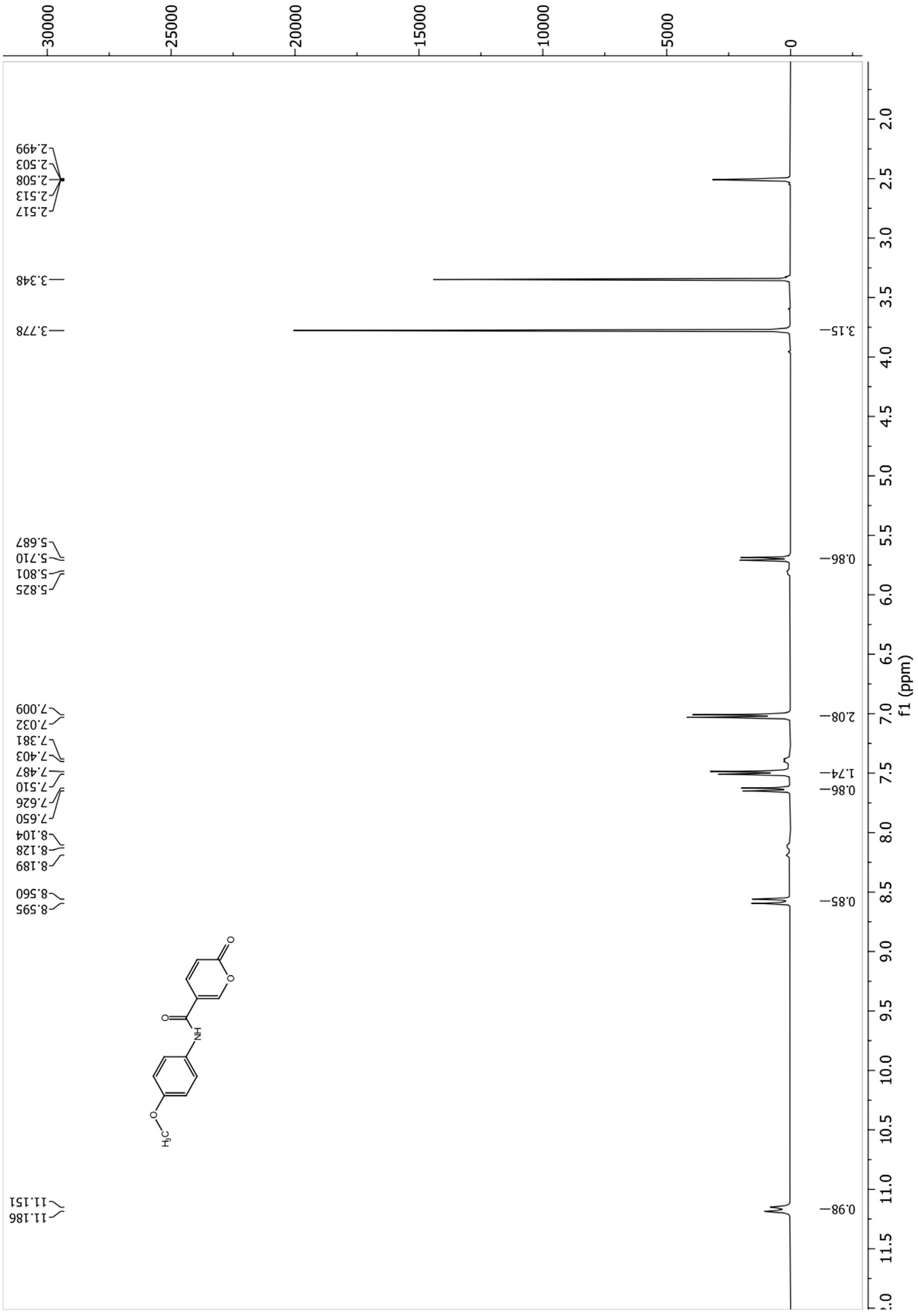

^{13}C NMR spectrum of compound **6**

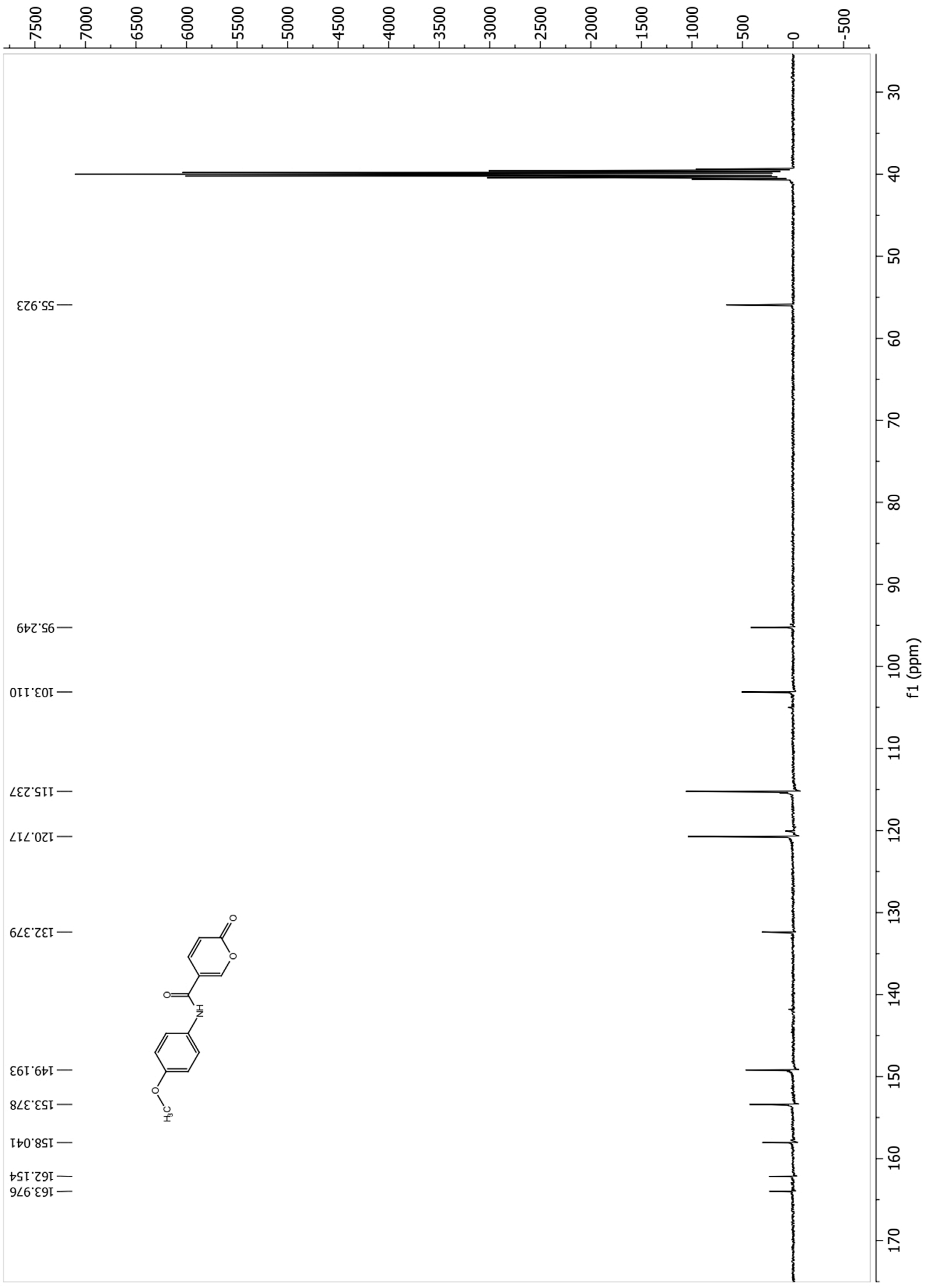

^{1}H NMR spectrum of compound **7**

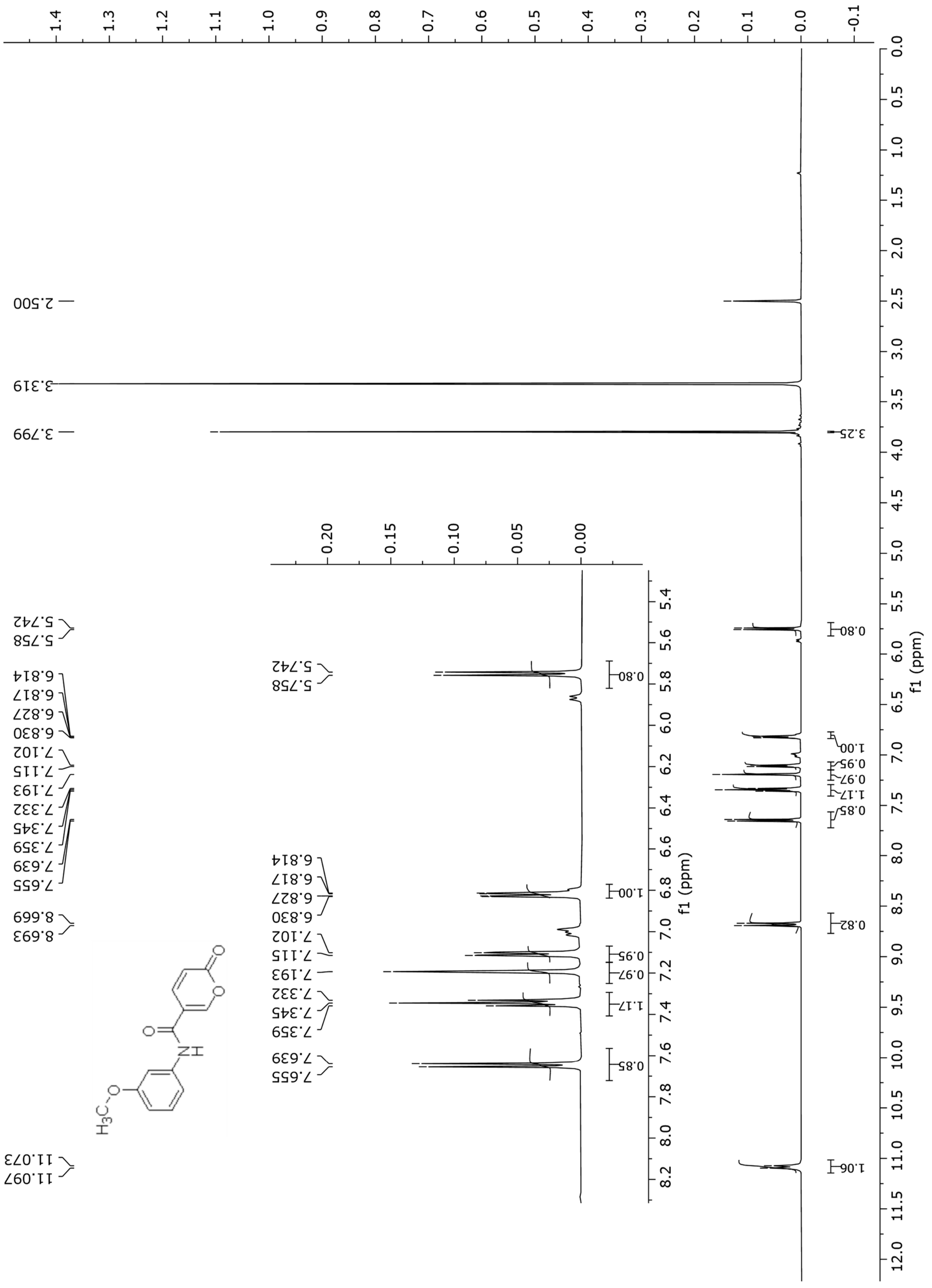

^{13}C NMR spectrum of compound **7**

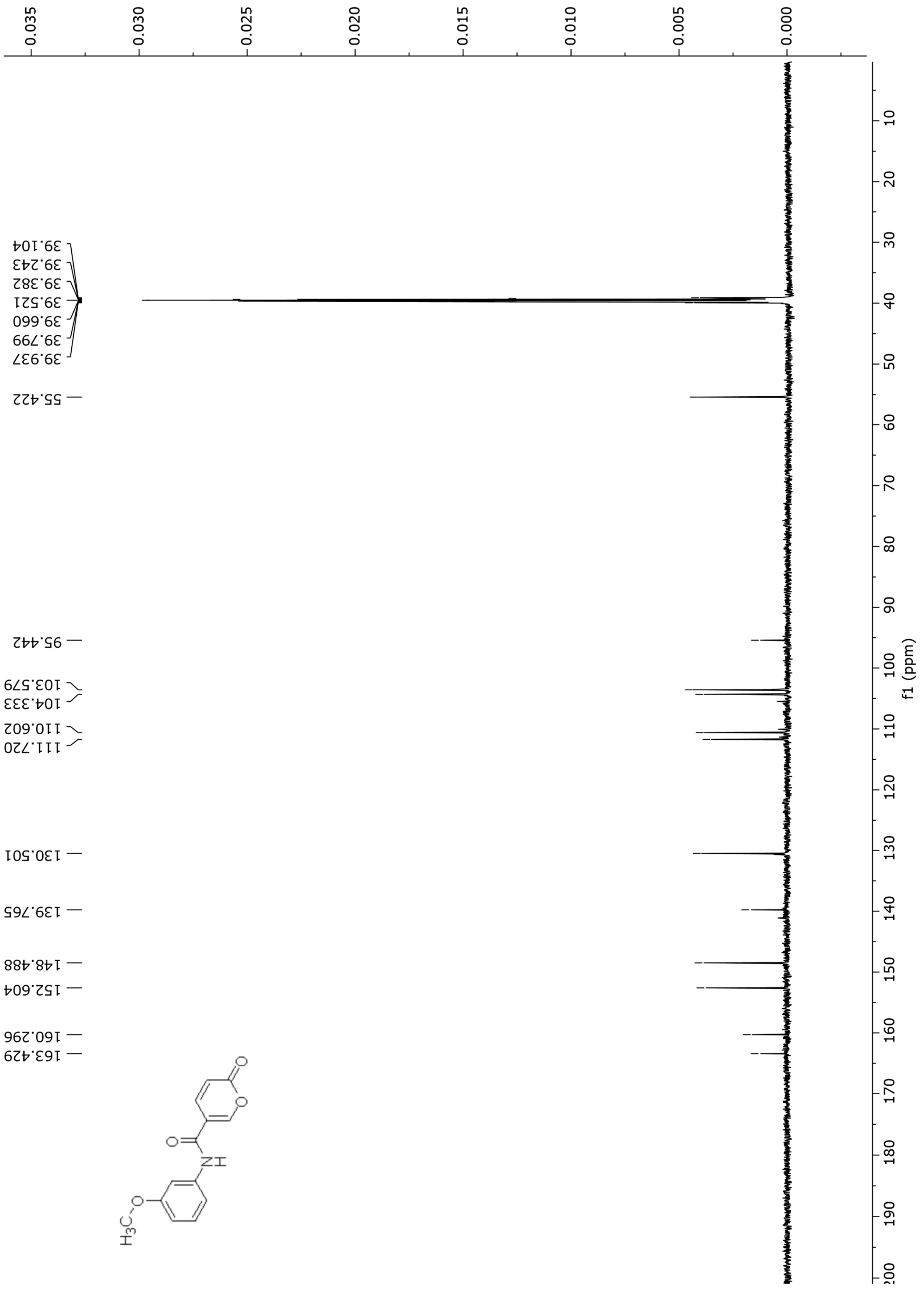

^{1}H NMR spectrum of compound **8**

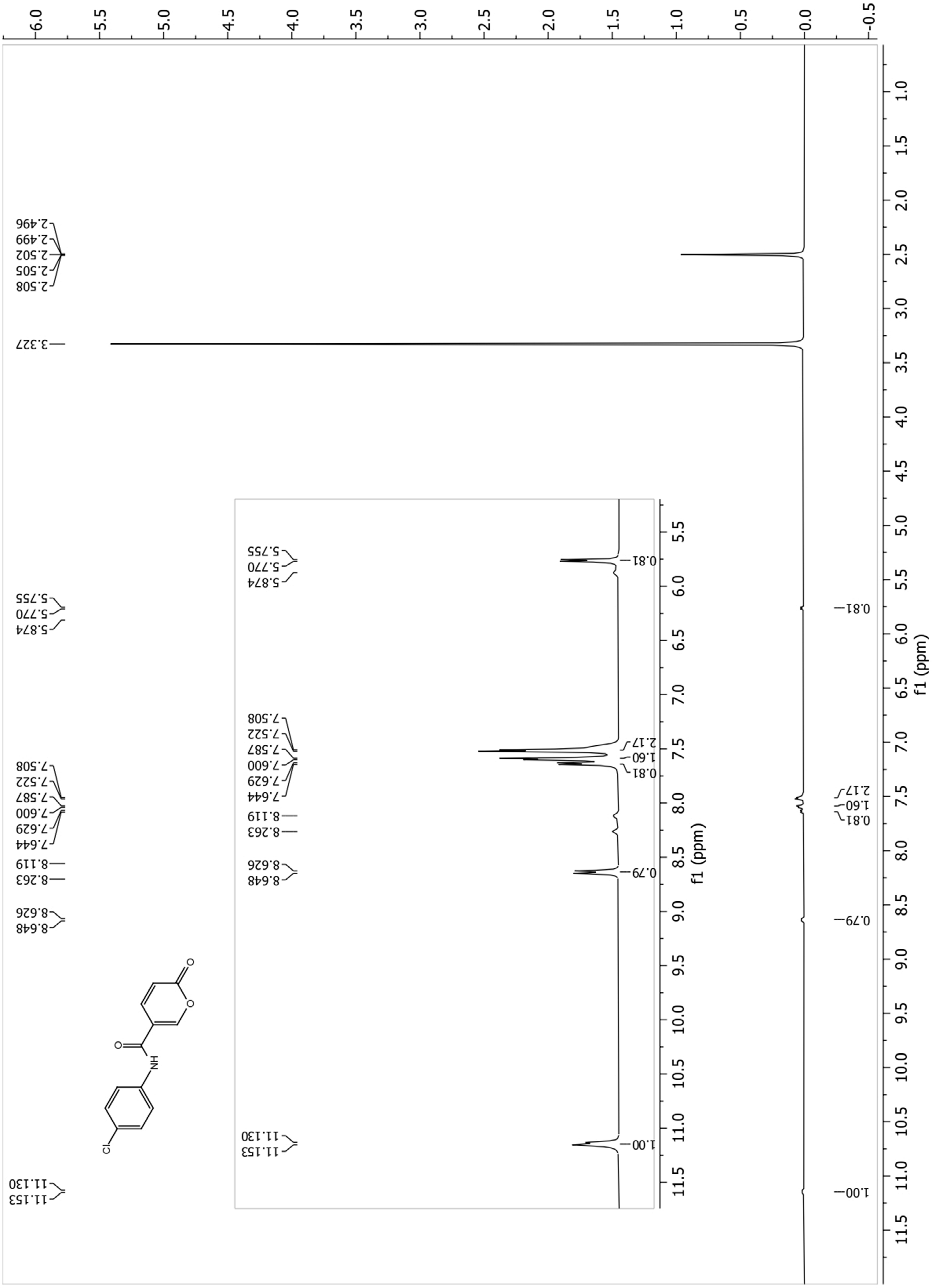

^{13}C NMR spectrum of compound **8**

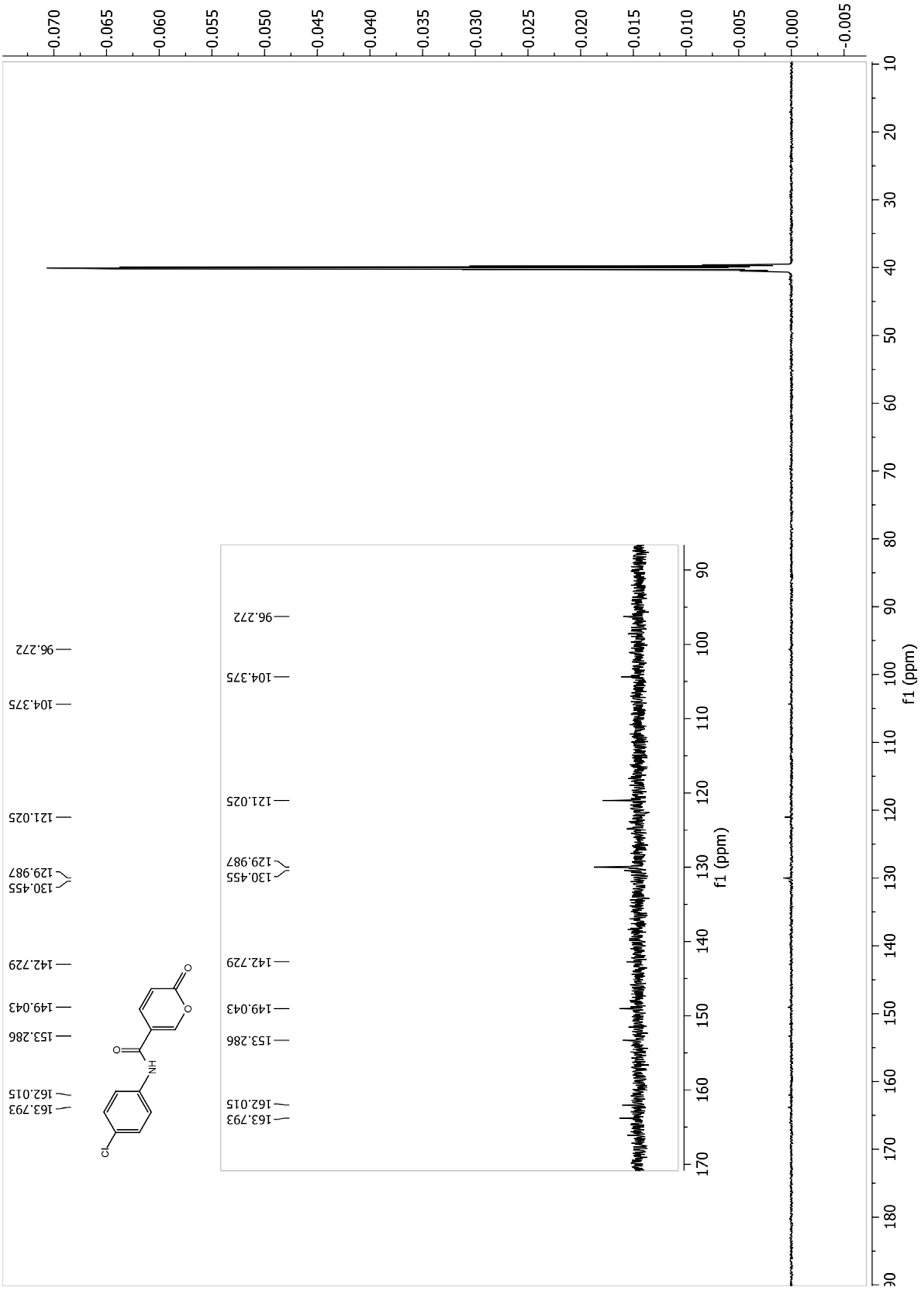

^{1}H NMR spectrum of compound **9**

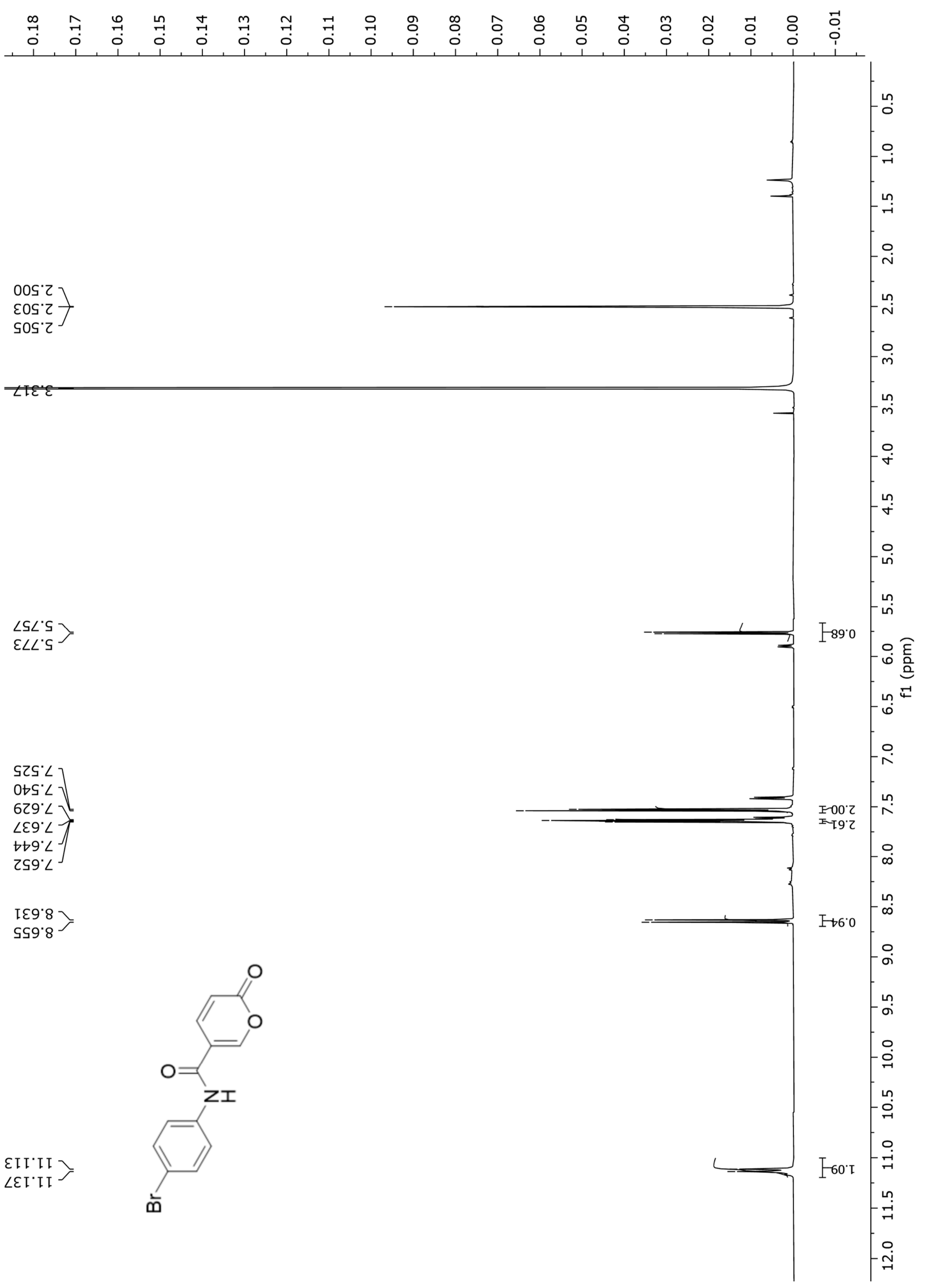

^{13}C NMR spectrum of compound **9**

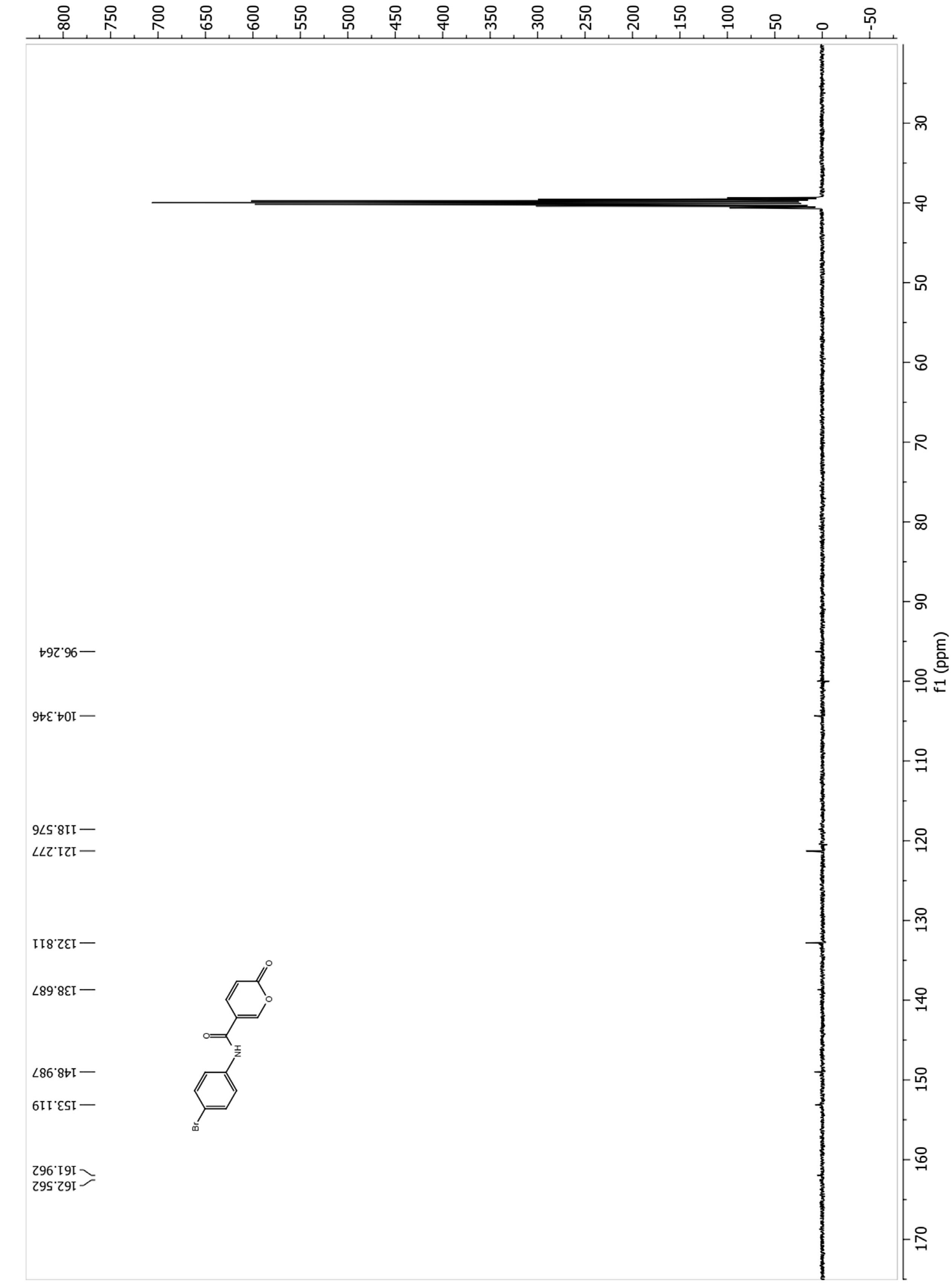

^{1}H NMR spectrum of compound **10**

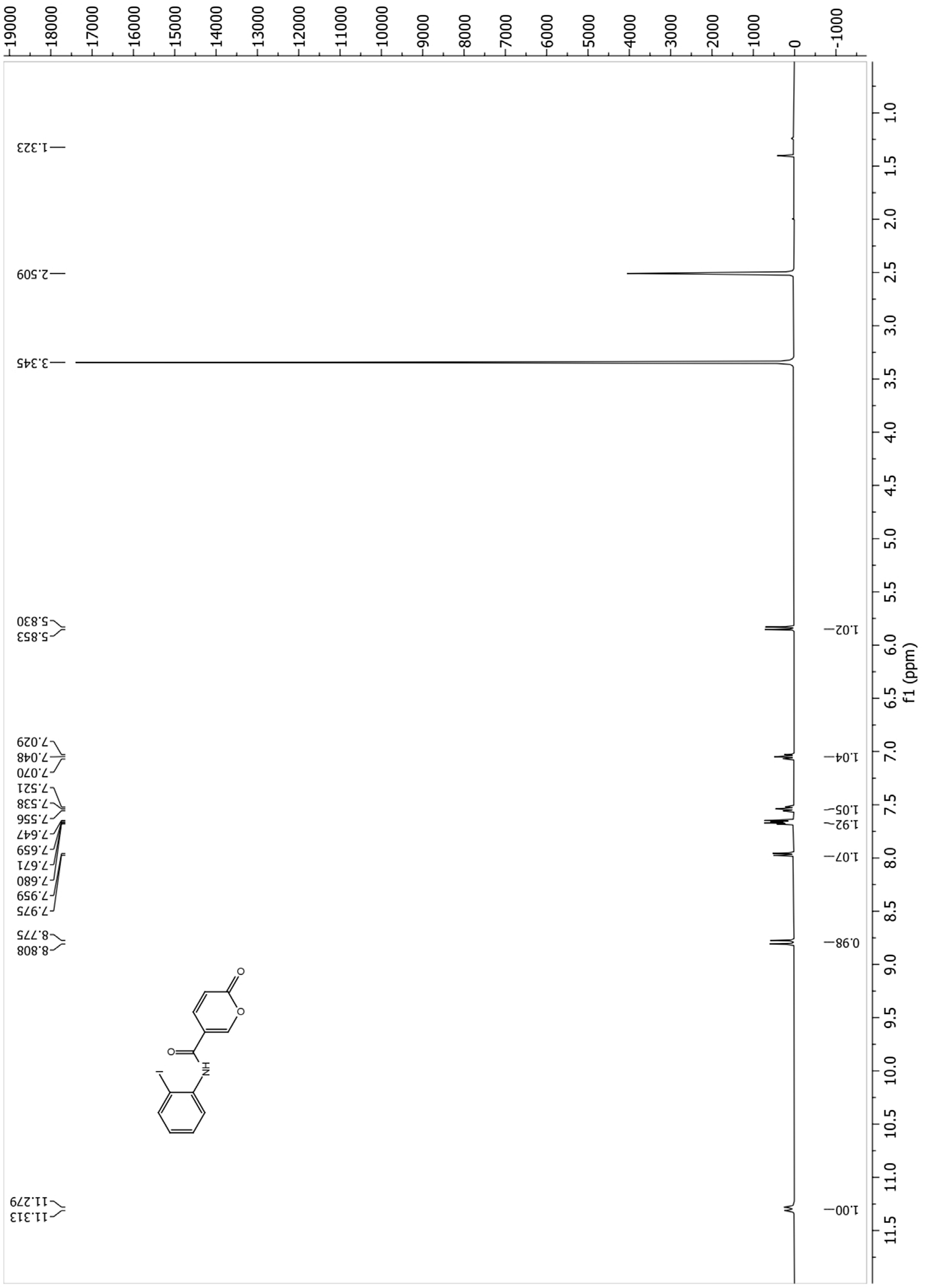

^{13}C NMR spectrum of compound **10**

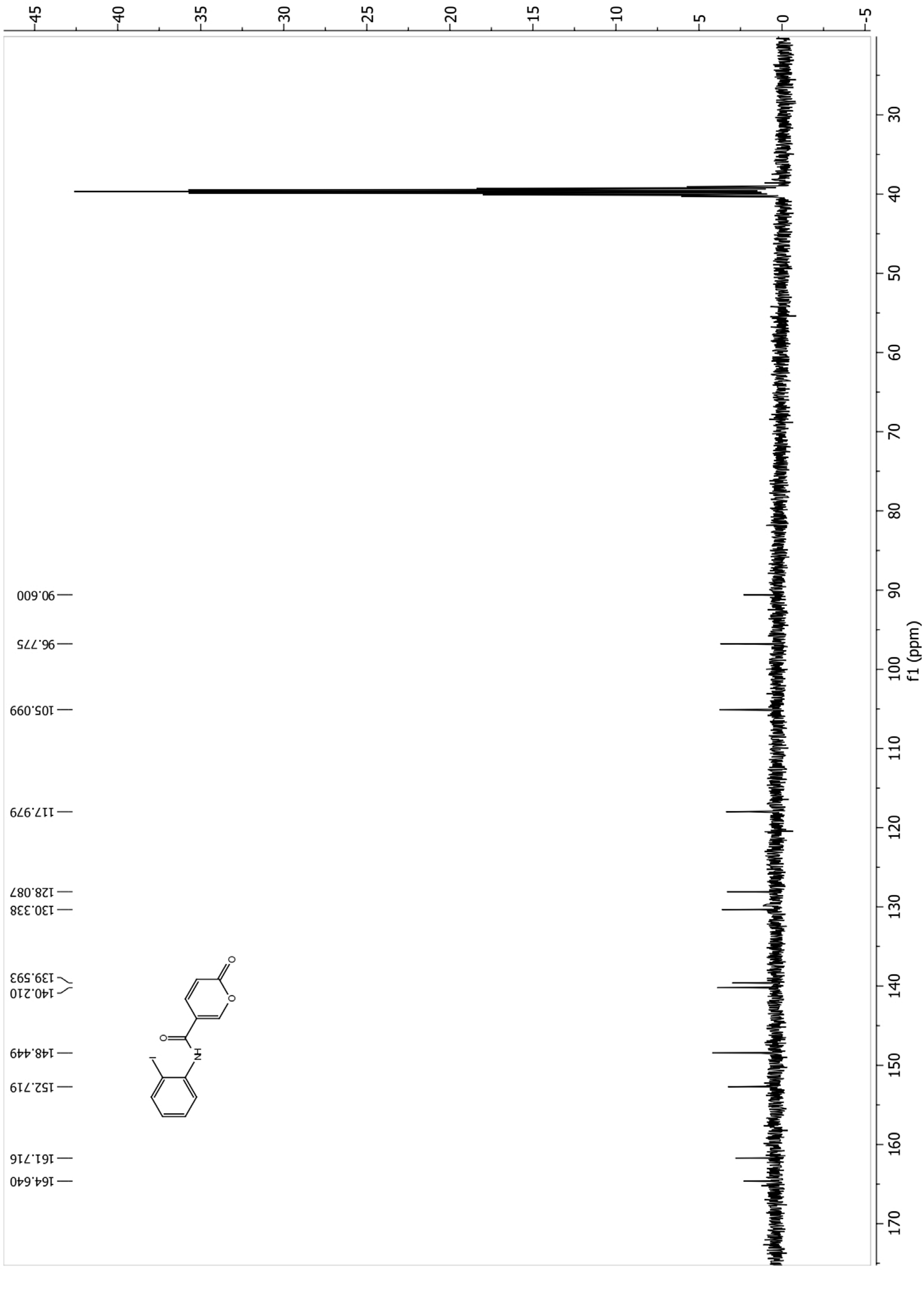

^{1}H NMR spectrum of compound **11**

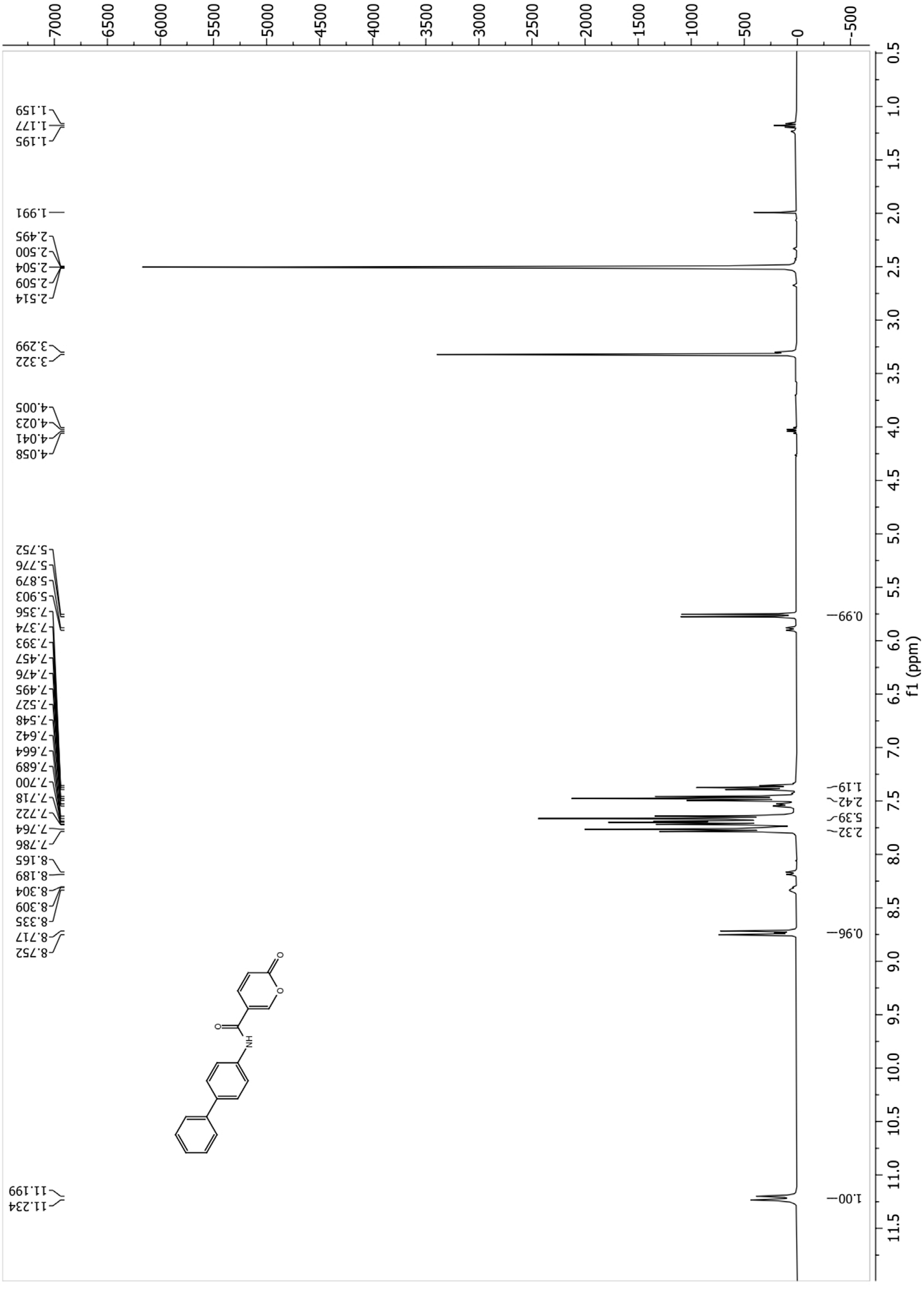

^{1}H-^{1}H NMR-NOESY spectrum of compound **11**

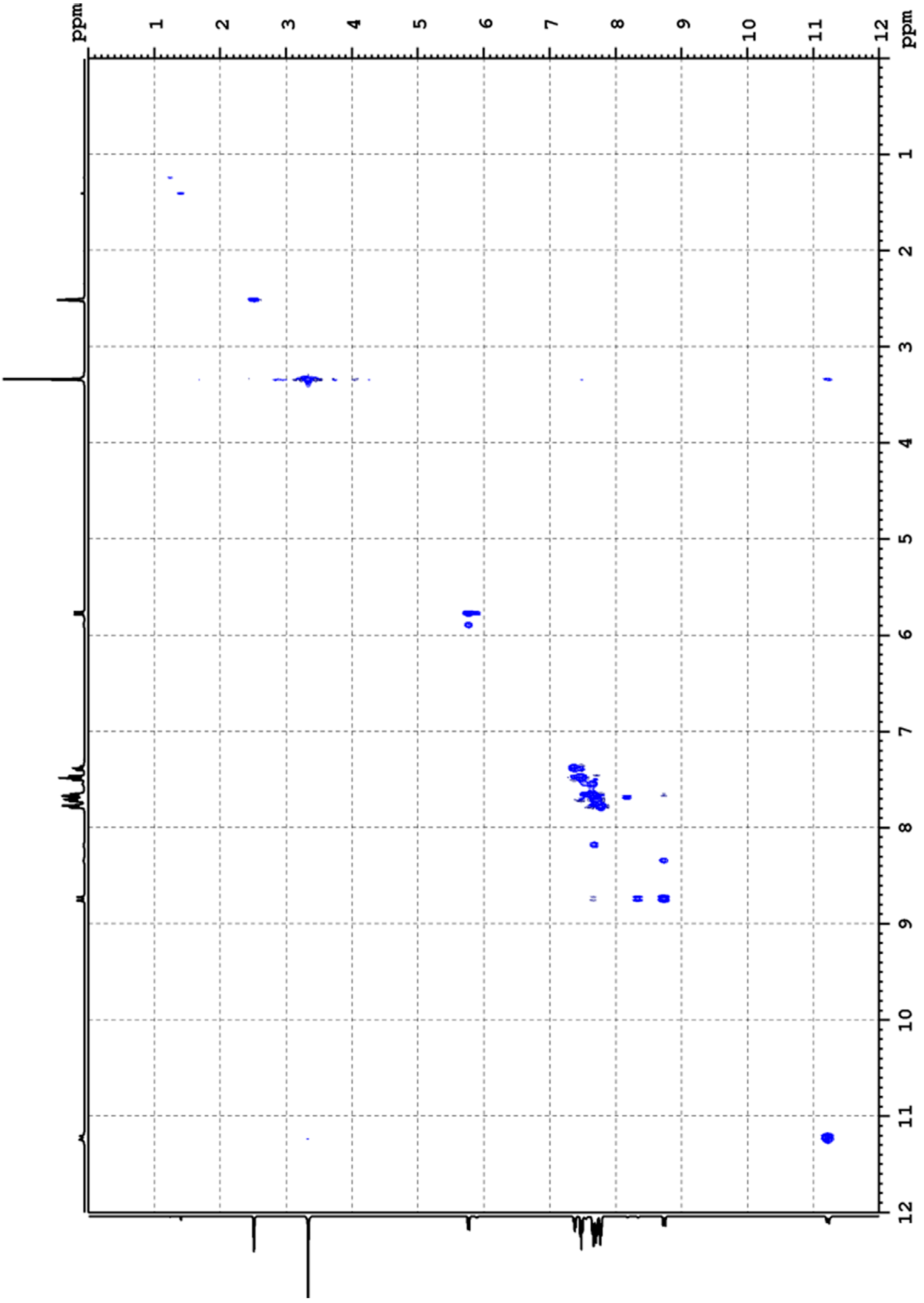

^{13}C NMR spectrum of compound **11**

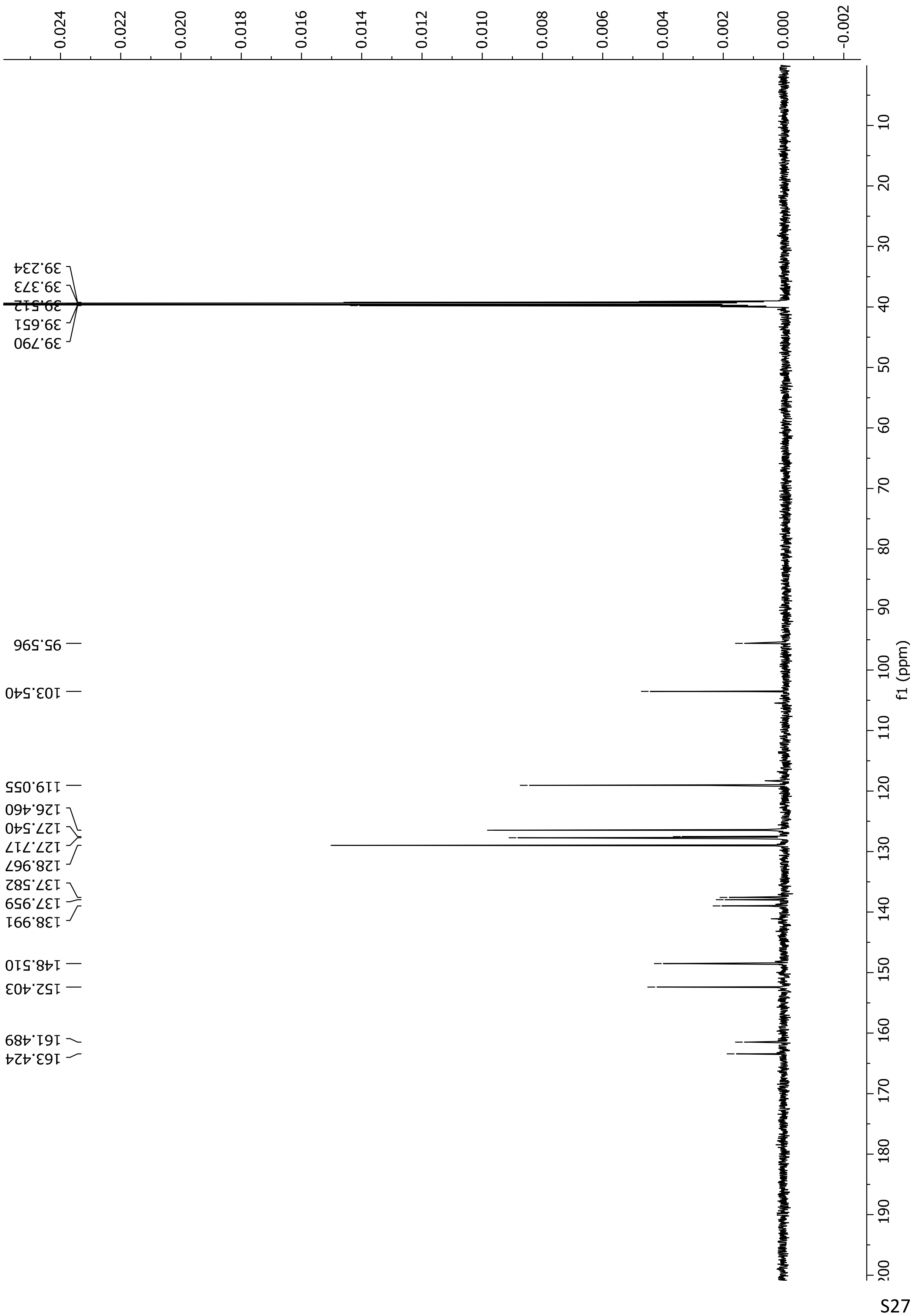

^{1}H–^{13}C HSQC spectrum of compound **11**

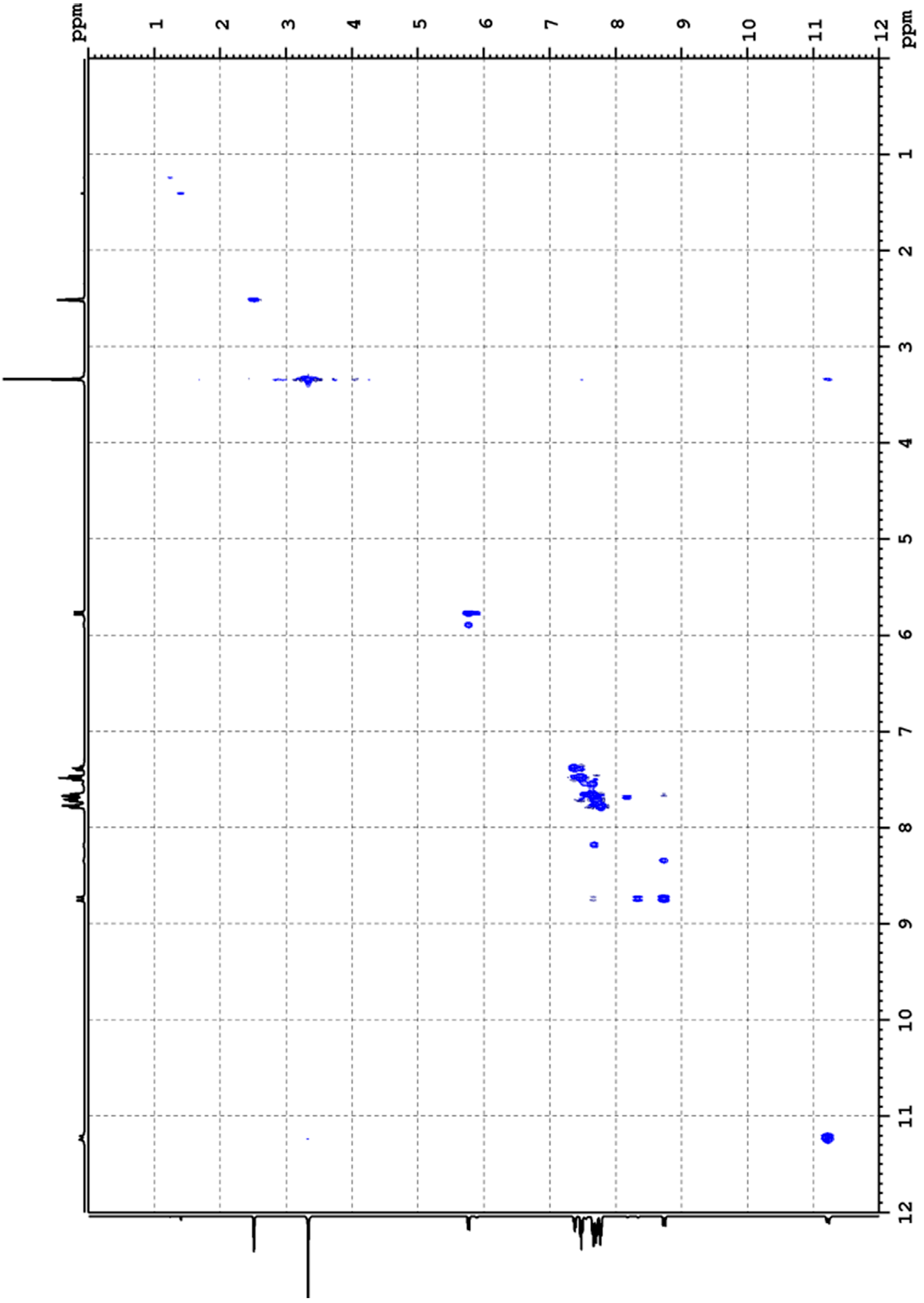

^{1}H NMR spectrum of compound **12**

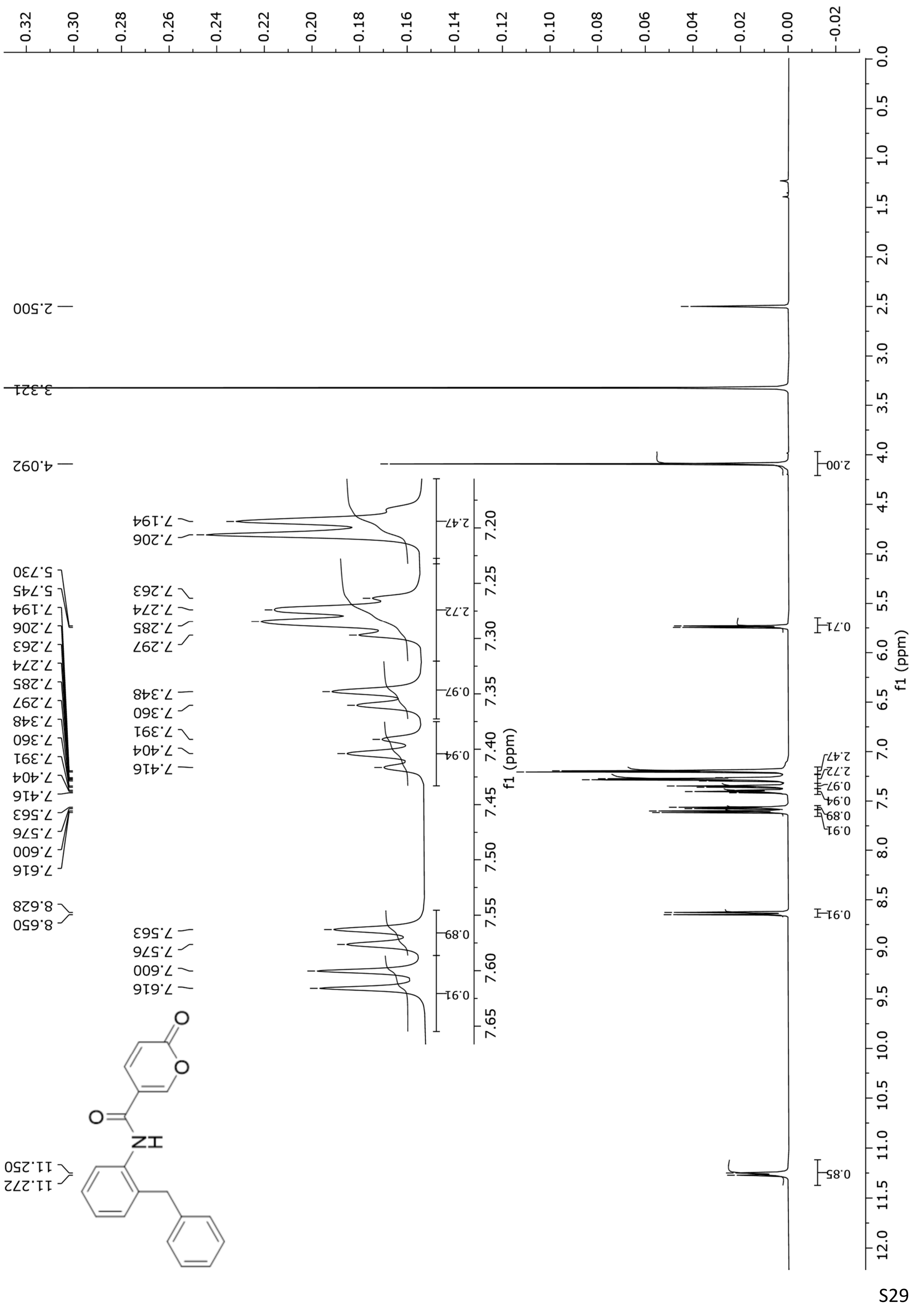

^{13}C NMR spectrum of compound **12**

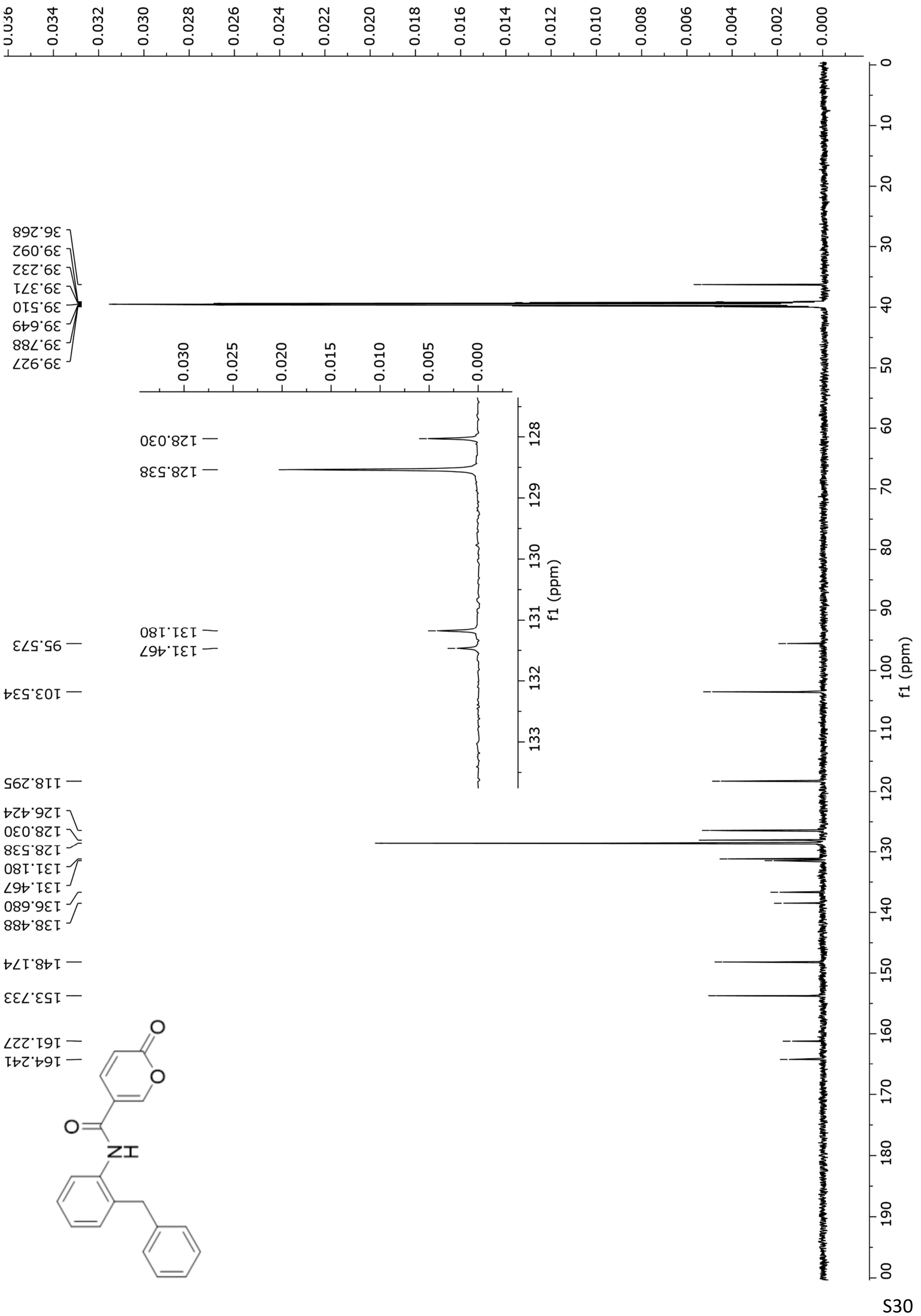

^{1}H NMR spectrum of compound **13**

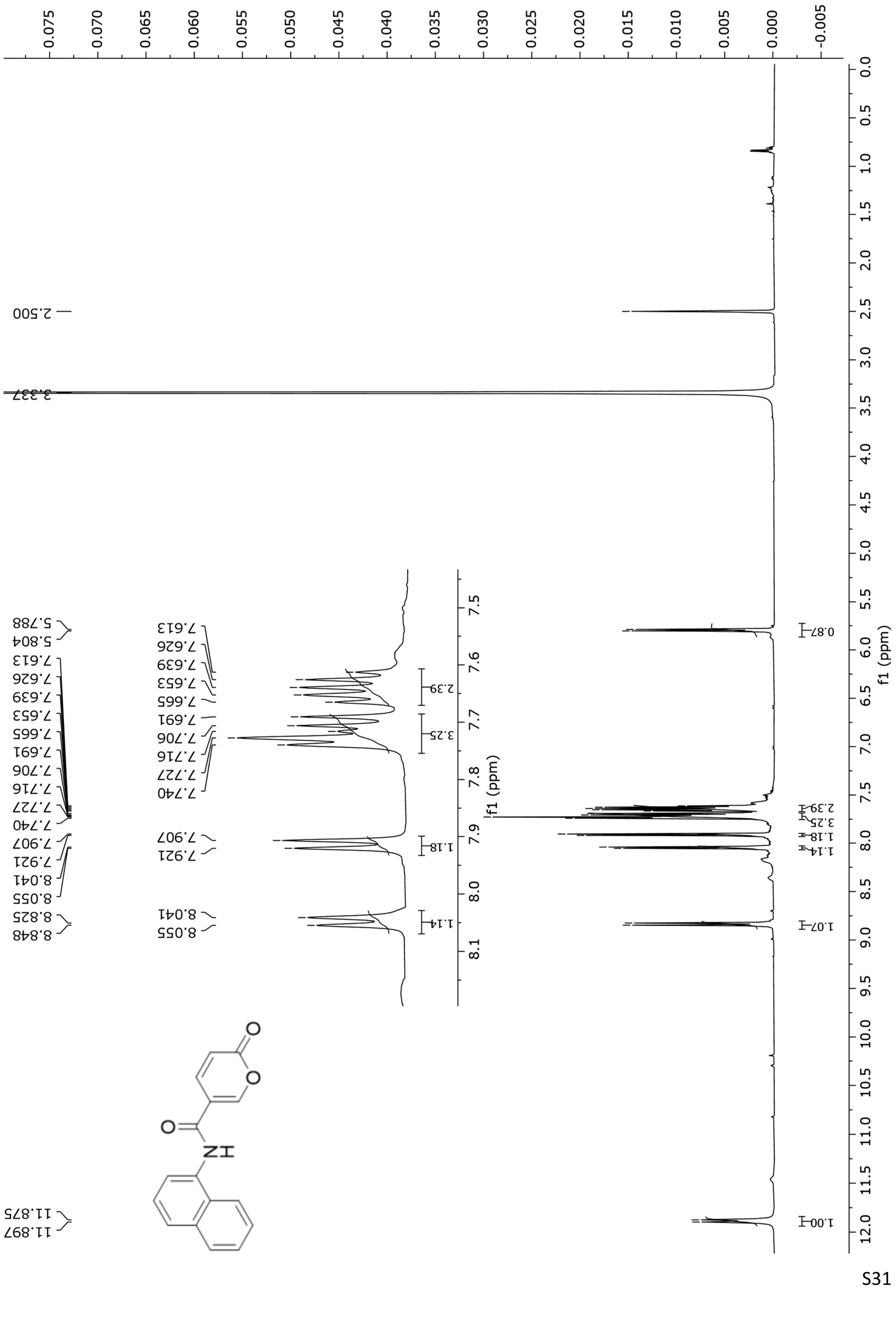

^{13}C NMR spectrum of compound **13**

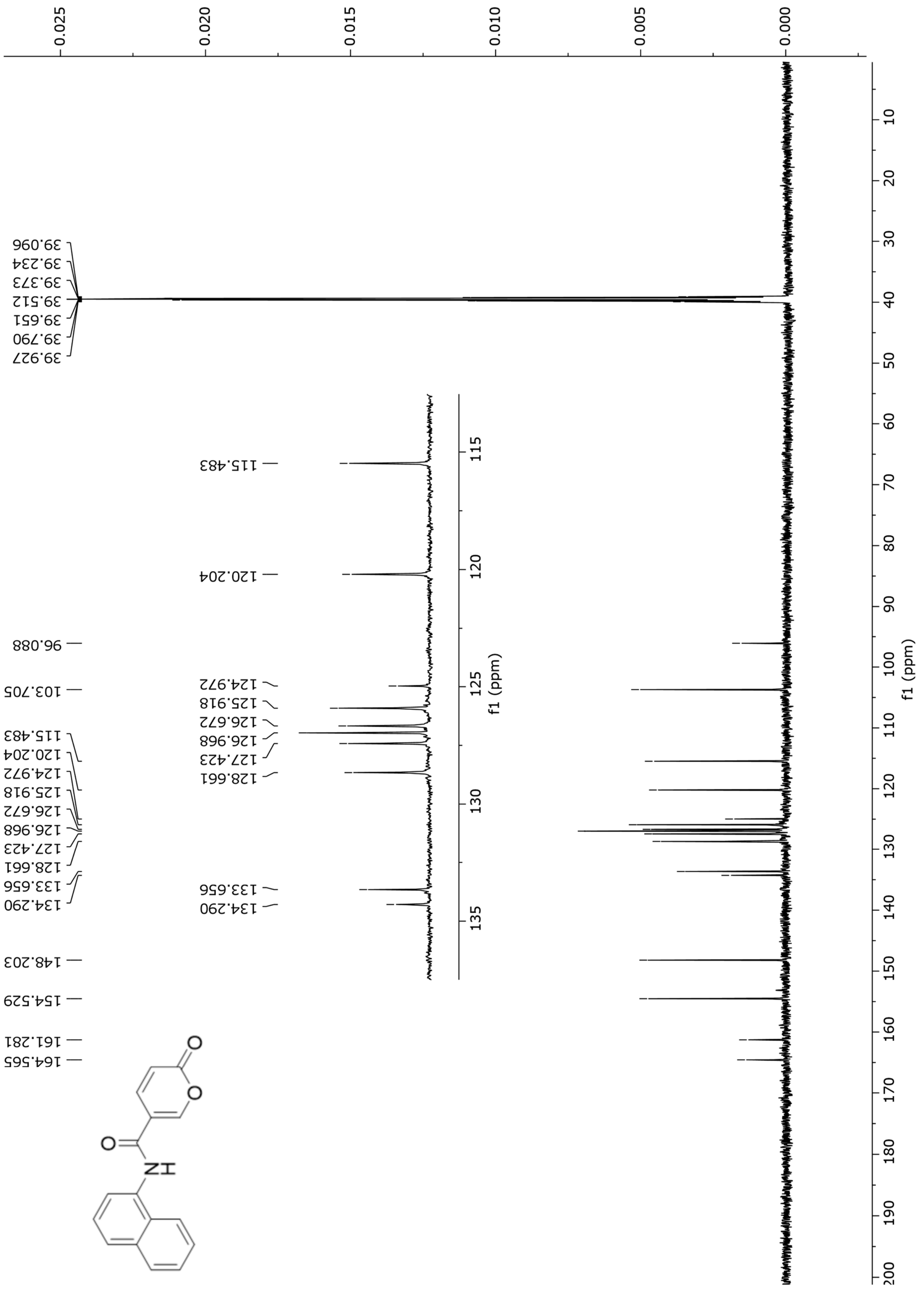

^{1}H NMR spectrum of compound **14**

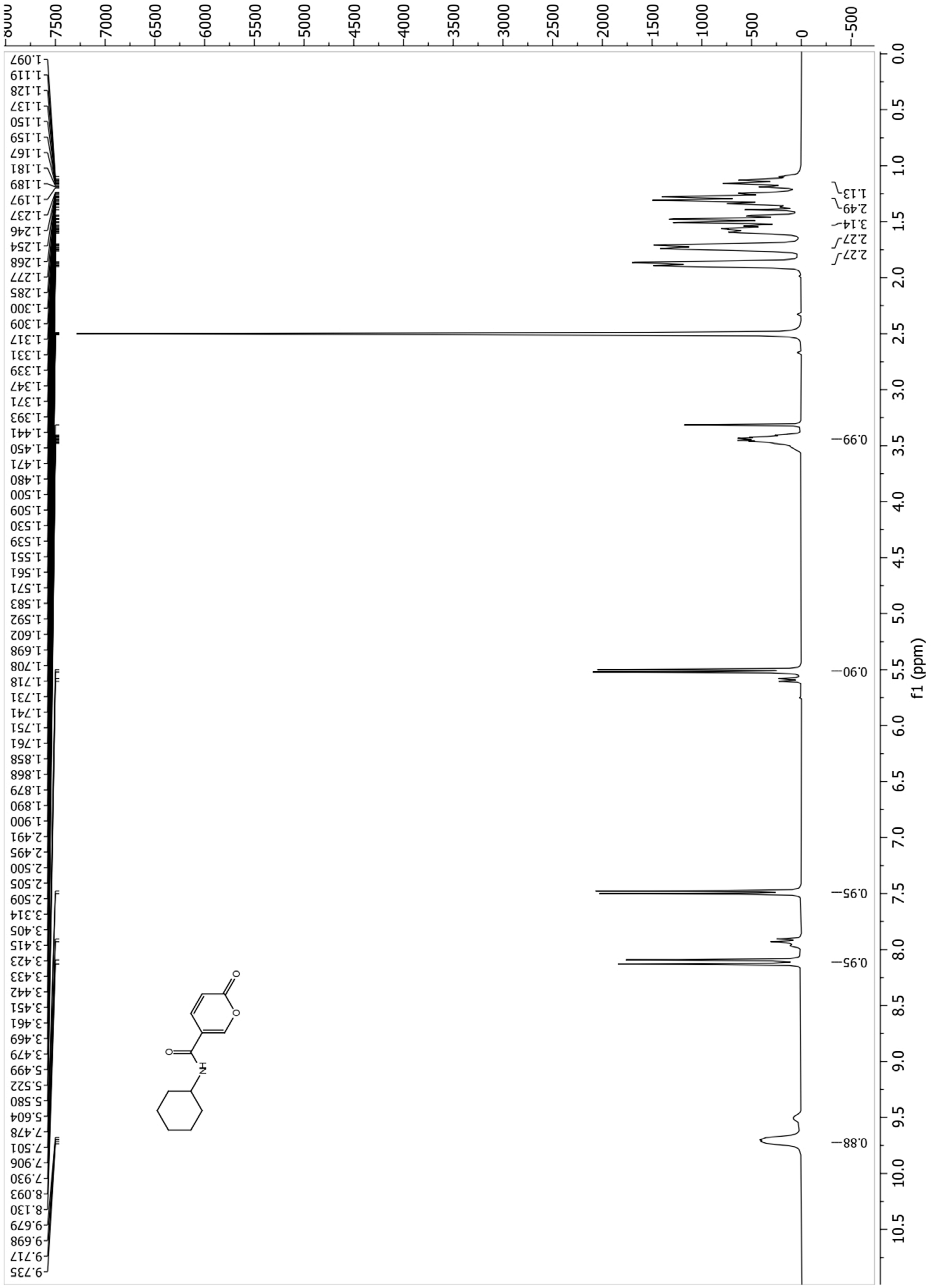

^1H NMR spectrum of compound **14** without (a) and in presence (b) of D_2O

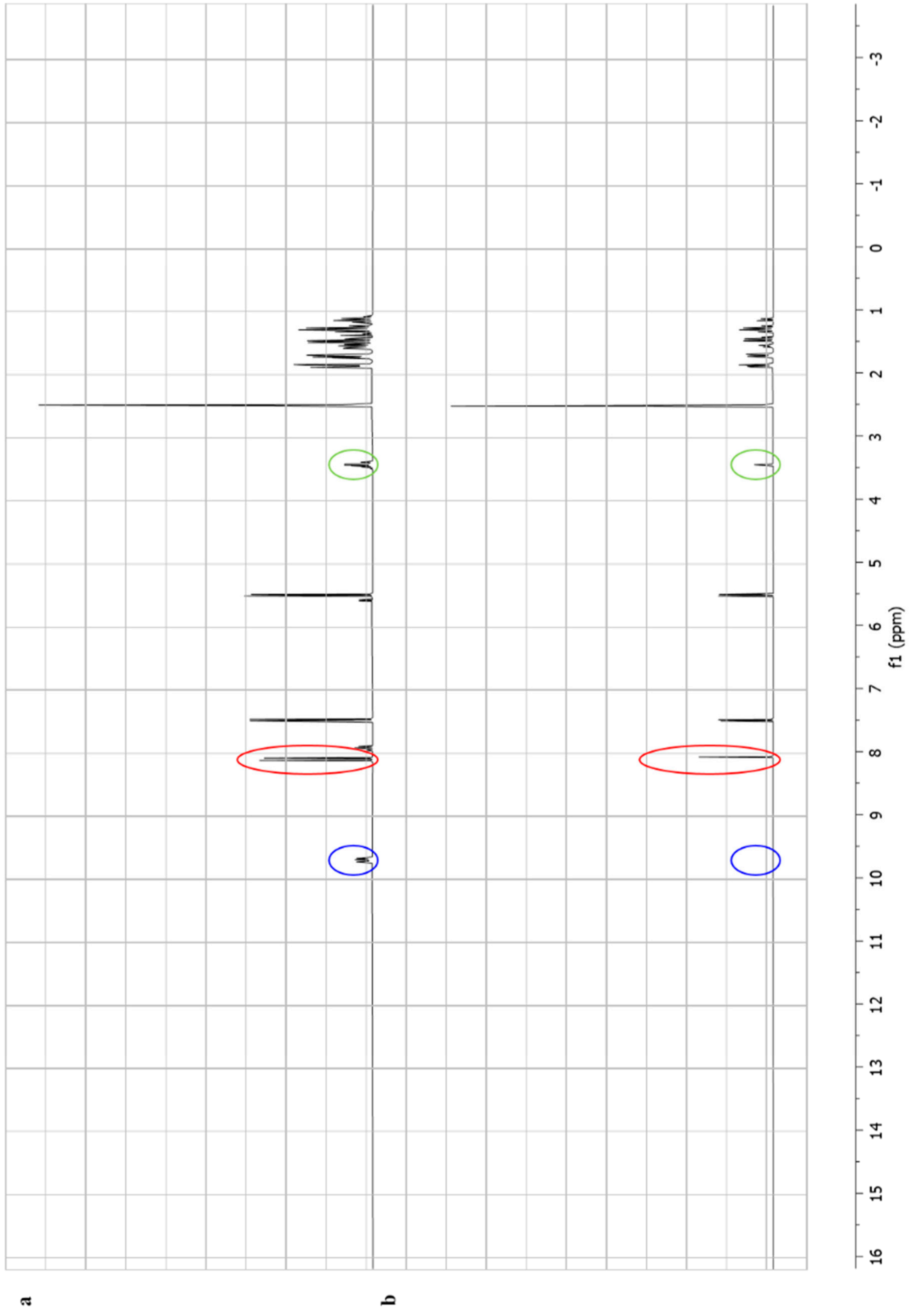

^{1}H-^{1}H NMR-COSY spectrum of compound **14**

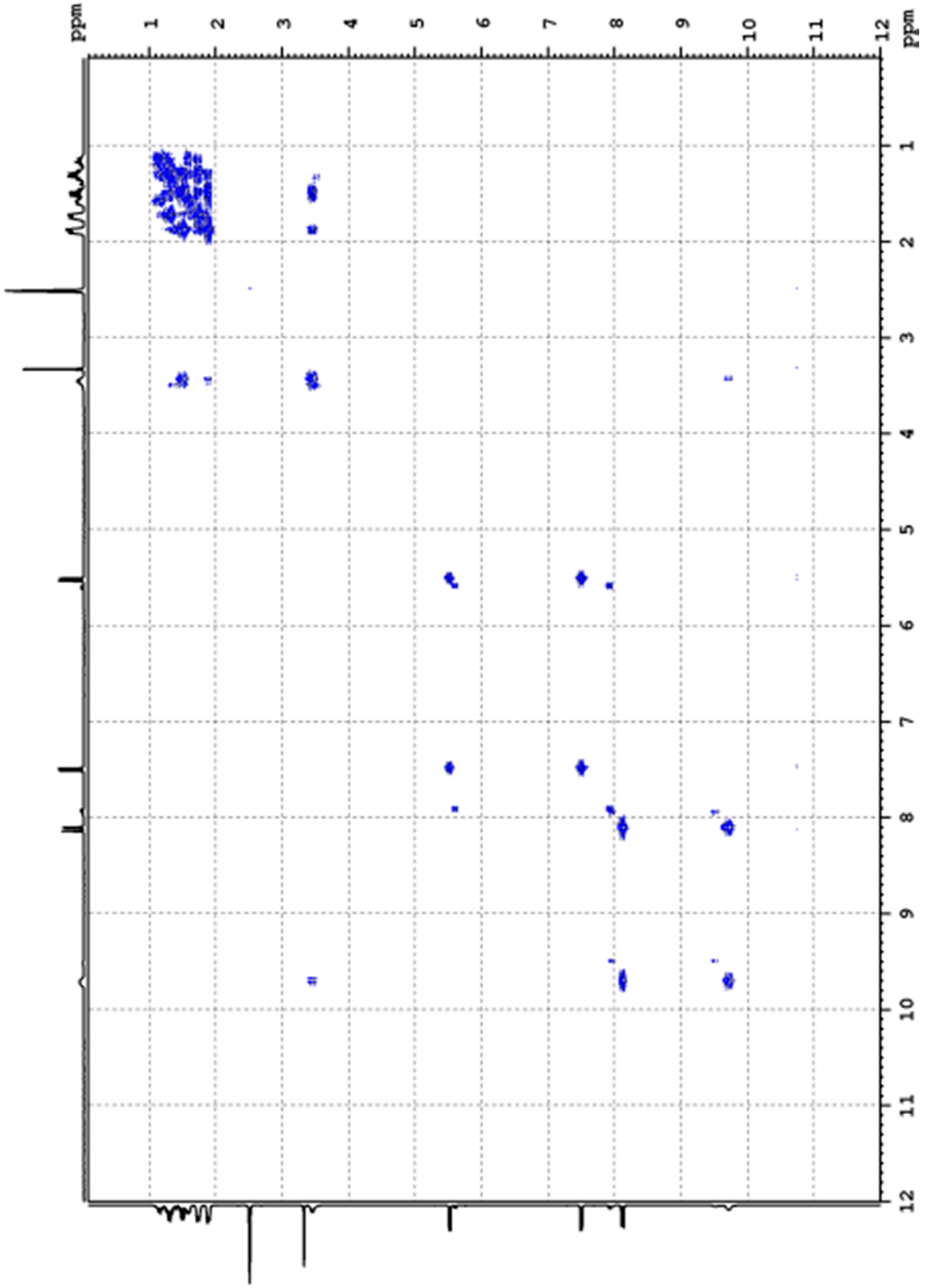

^{1}H-^{1}H NMR-NOESY spectrum of compound **14**

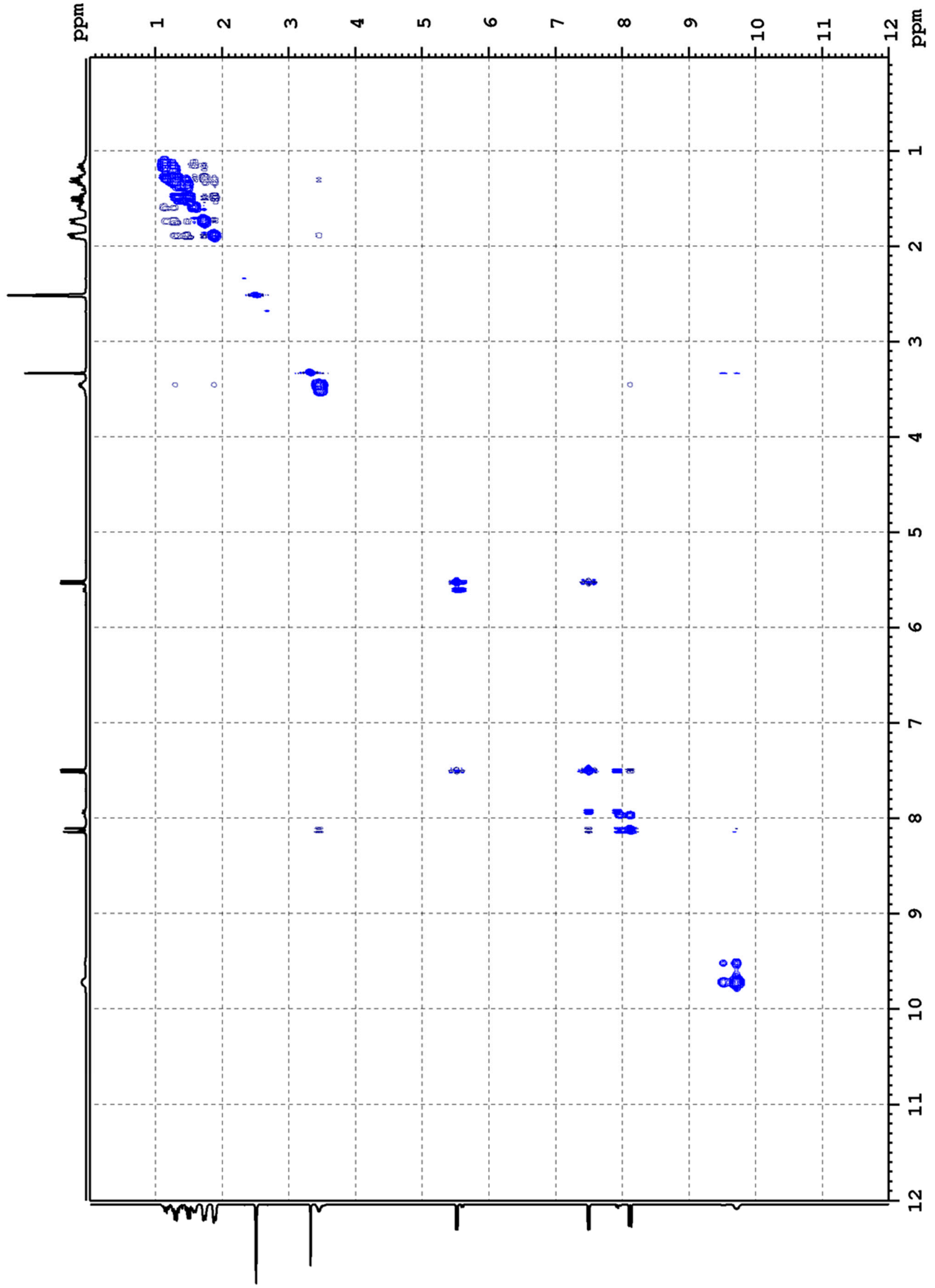

^{13}C NMR spectrum of compound **14**

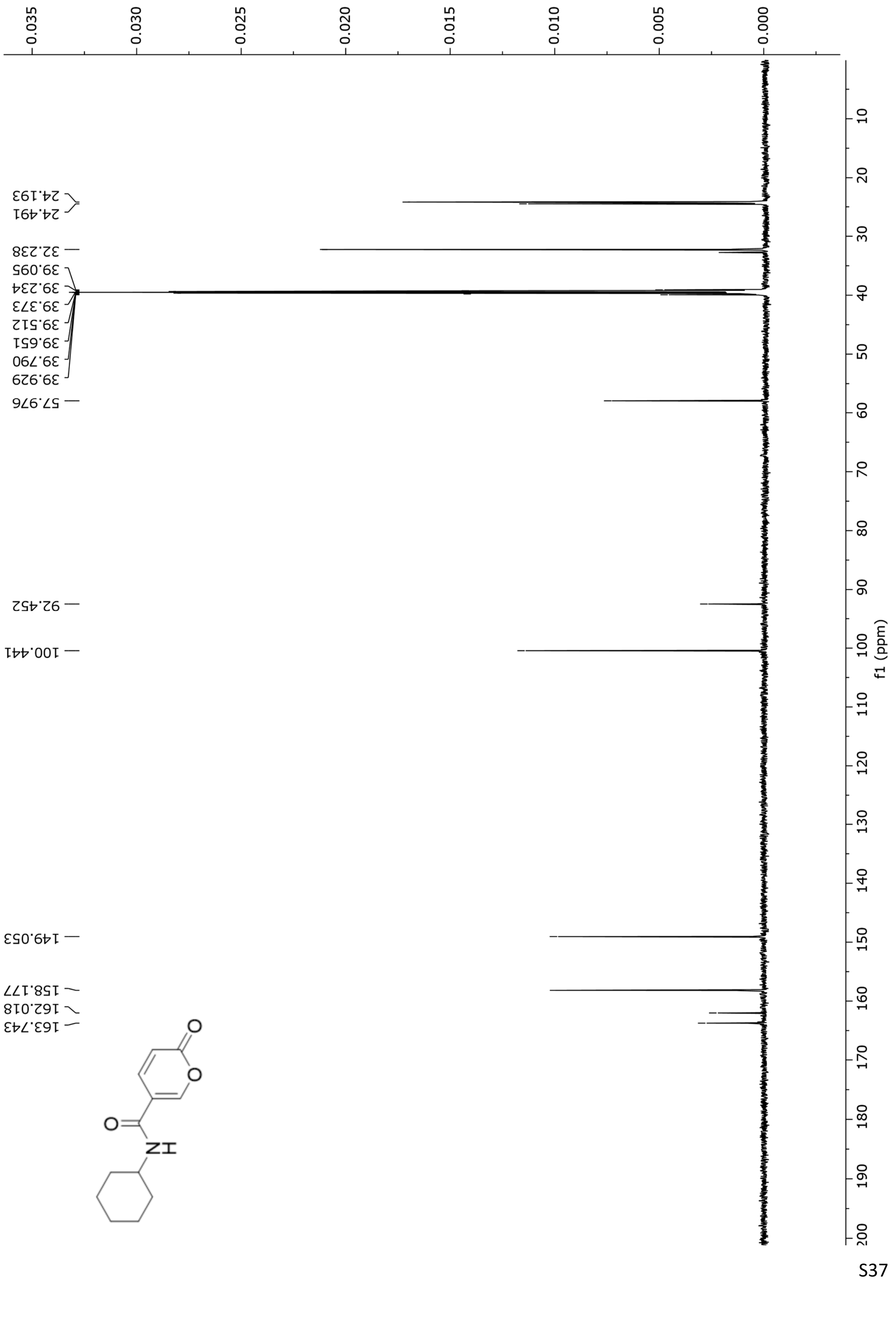

^{1}H NMR spectrum of compound **15**

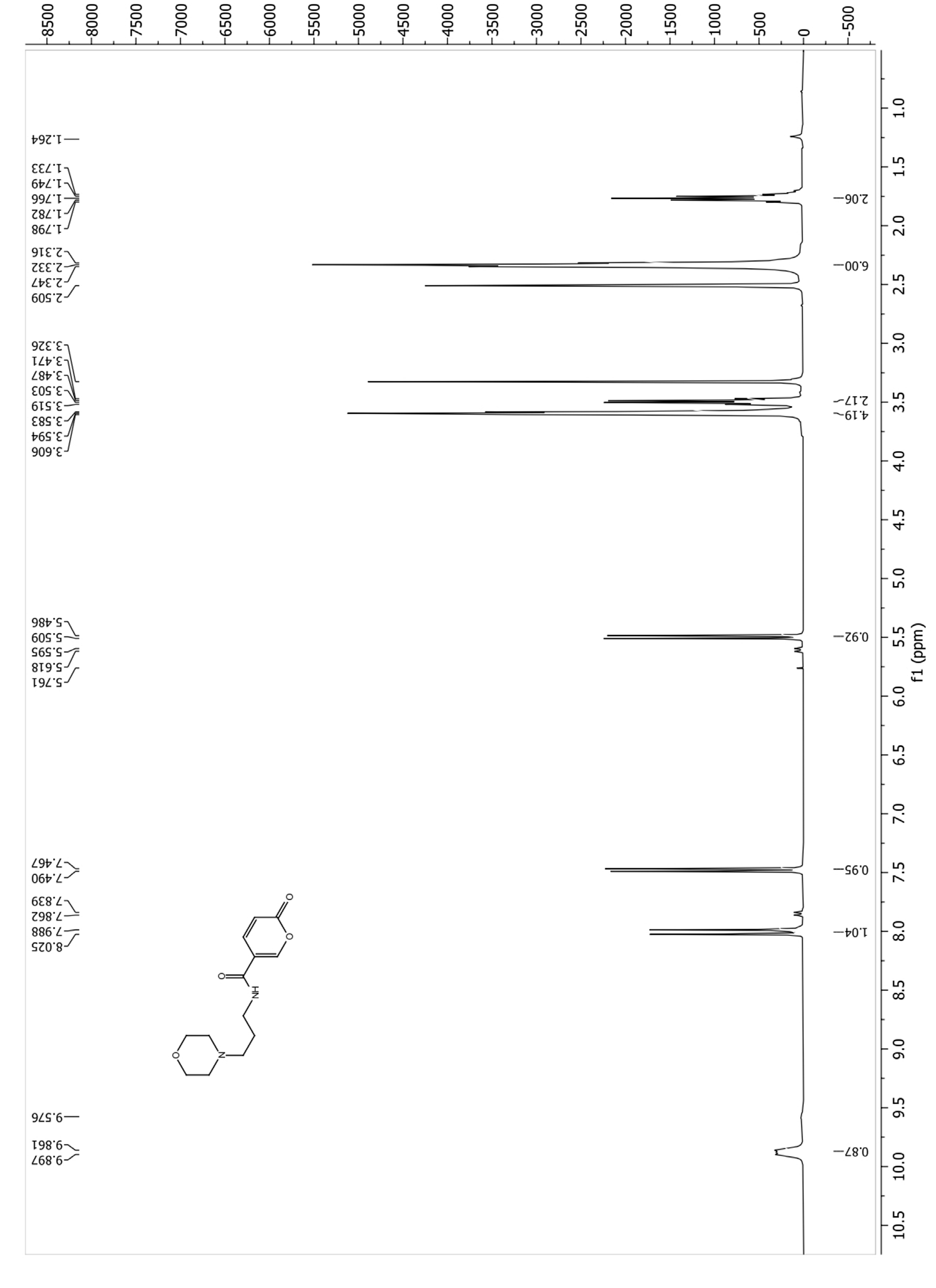

^{13}C NMR spectrum of compound **15**

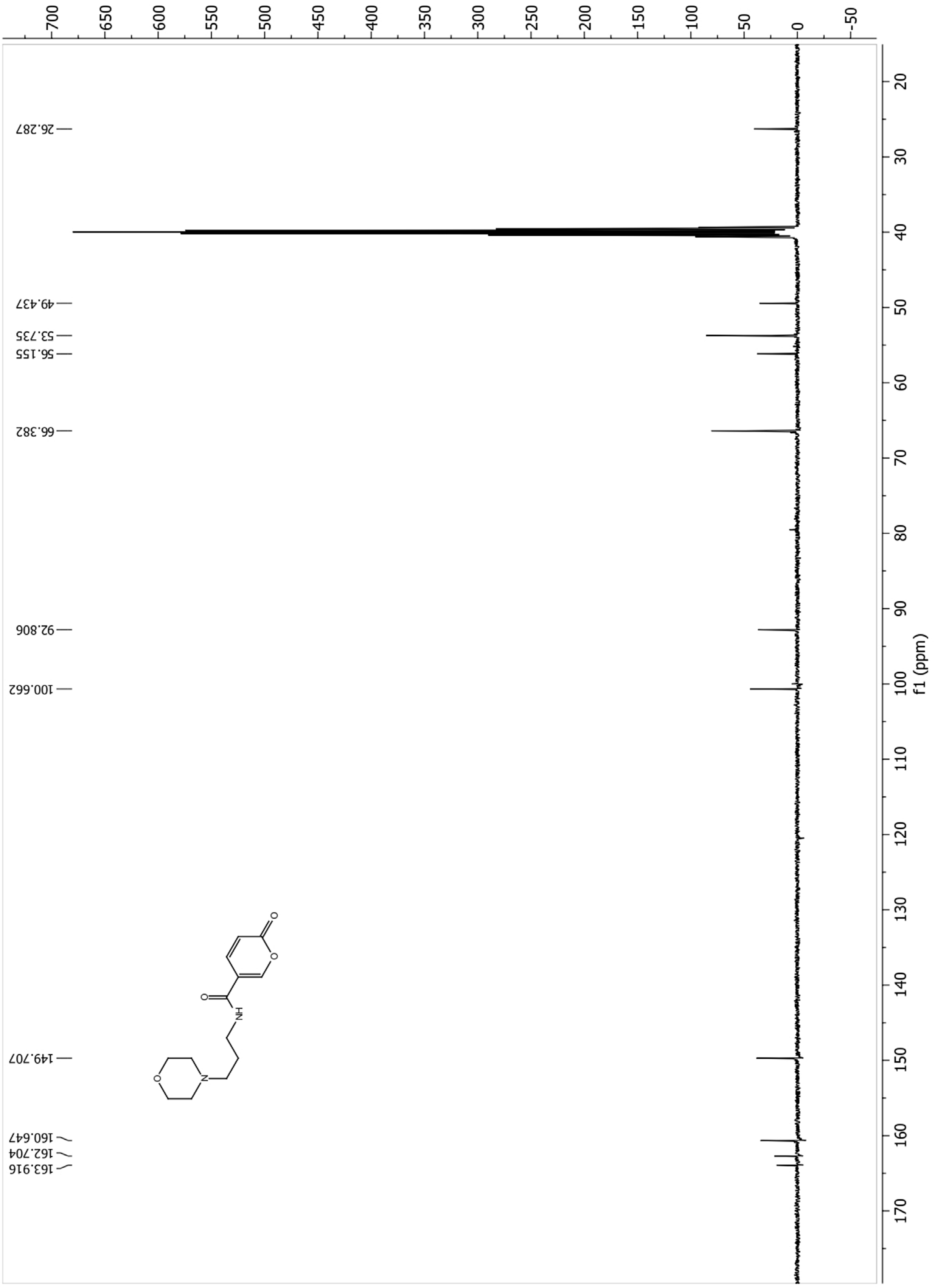

HPLC chromatograms of compounds 2-15

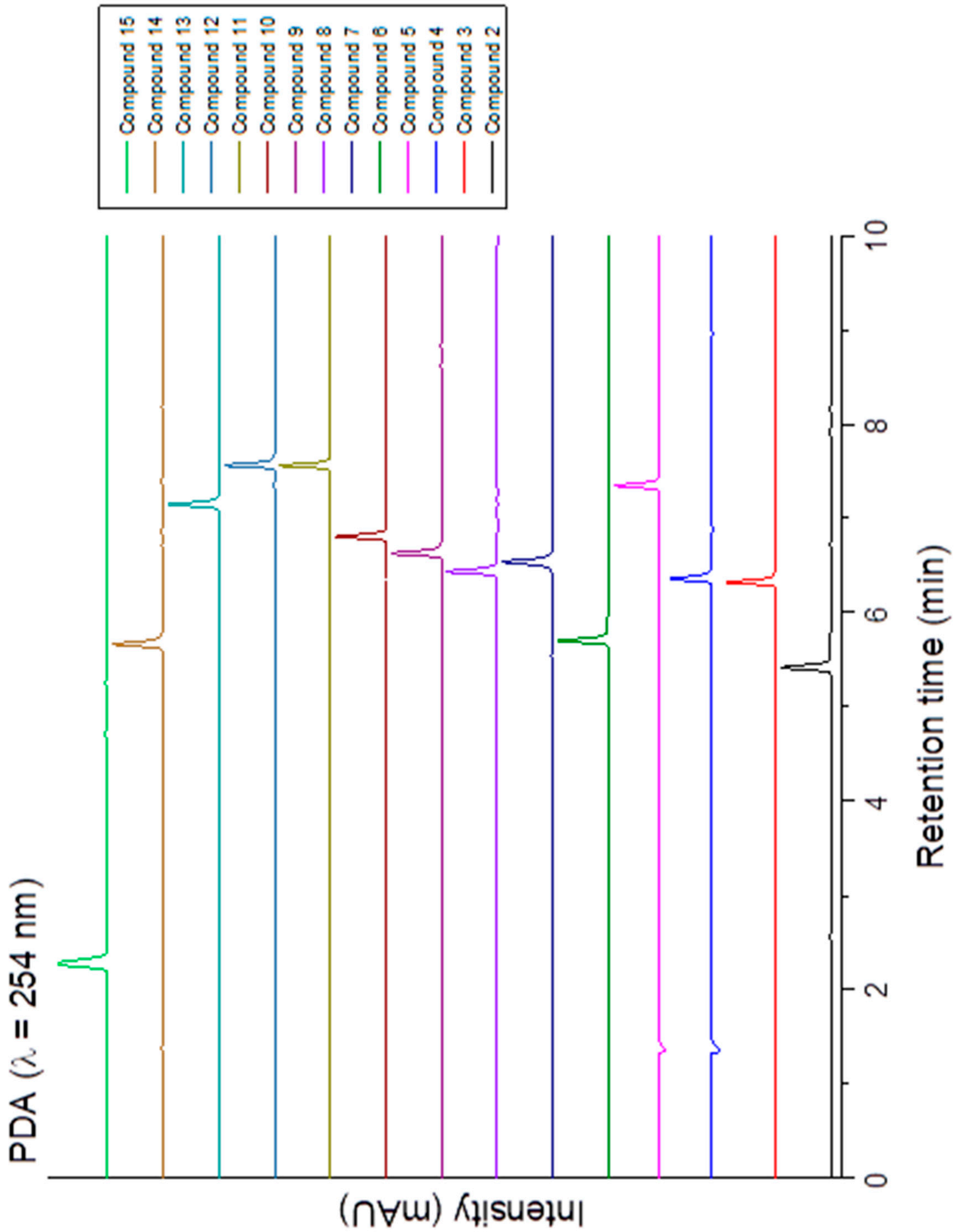

Enzyme inhibition curves of compounds 2-15

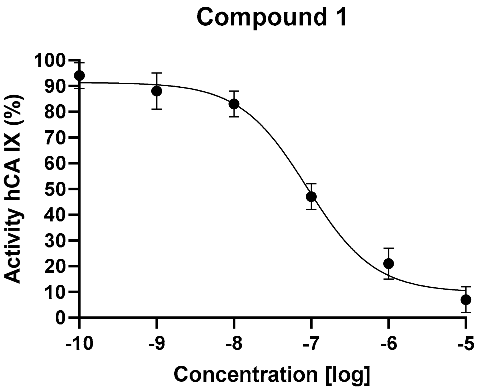

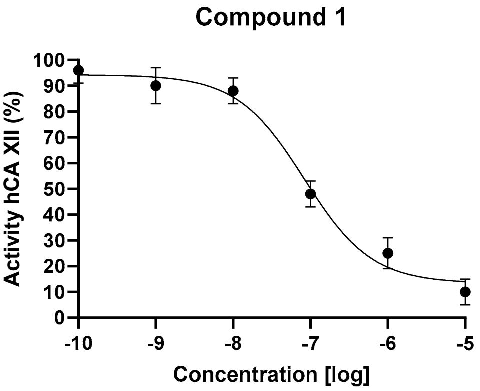

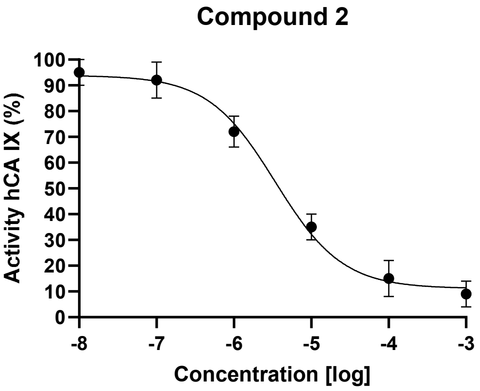

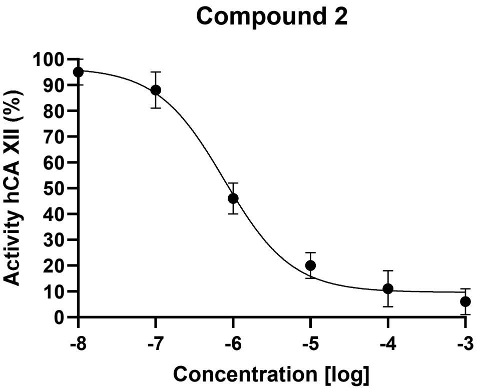

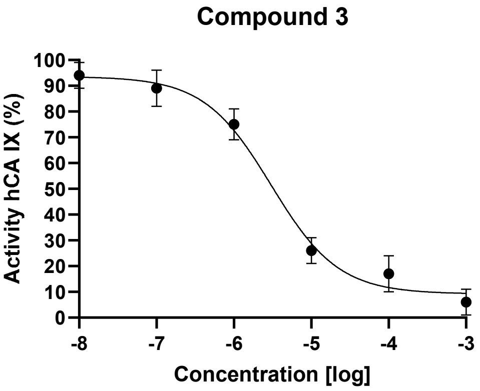

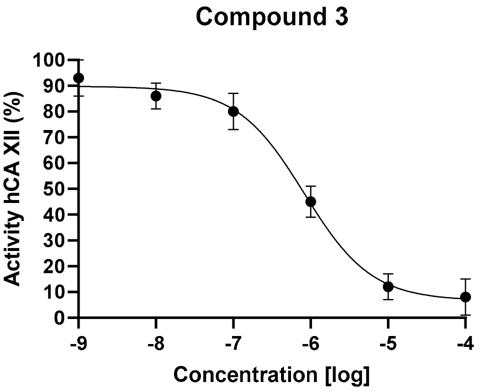

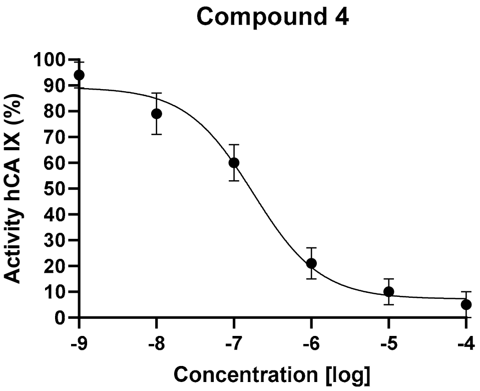

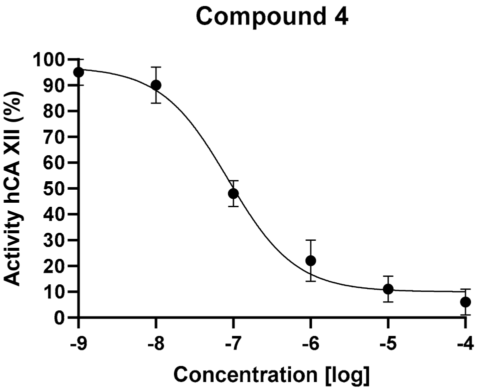

Compound 9

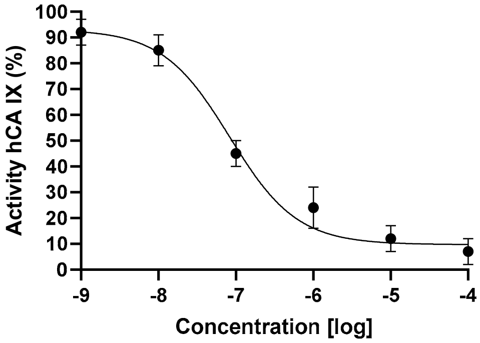

Compound 9

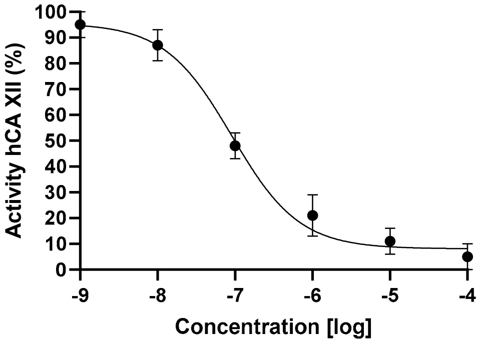

Compound 10

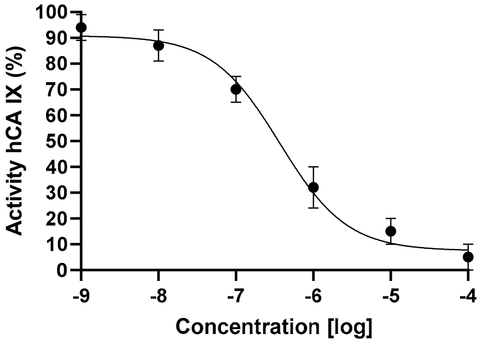

Compound 10

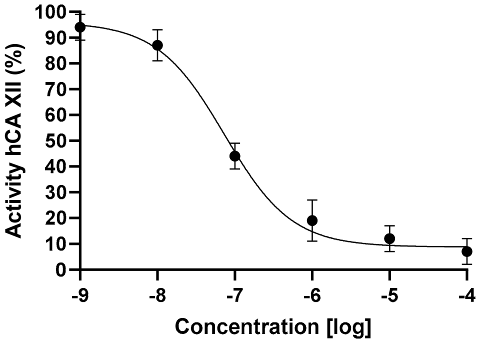

Compound 11

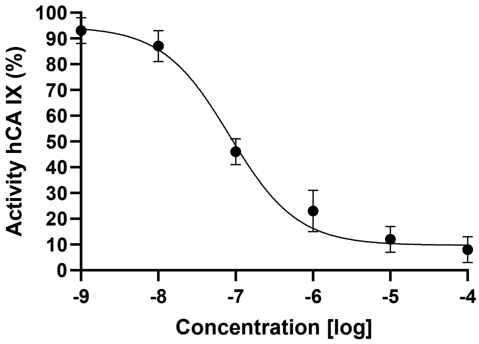

Compound 11

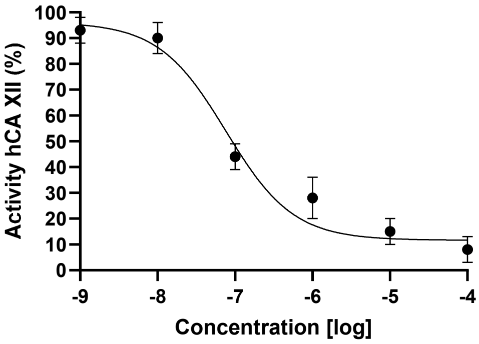

Compound 12

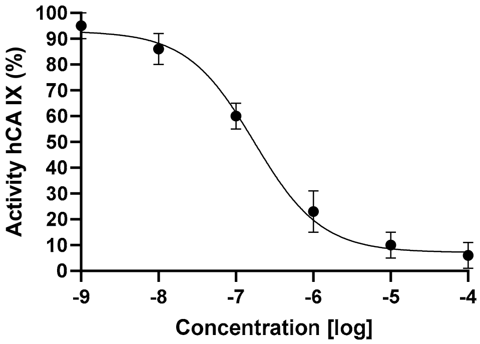

Compound 12

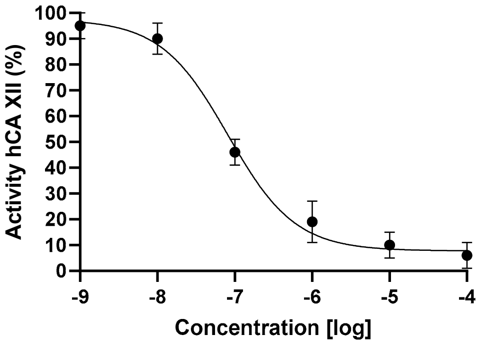

Compound 13

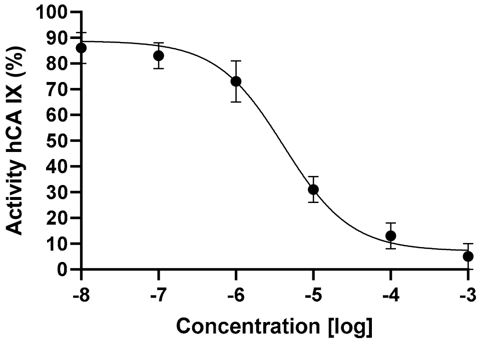

Compound 13

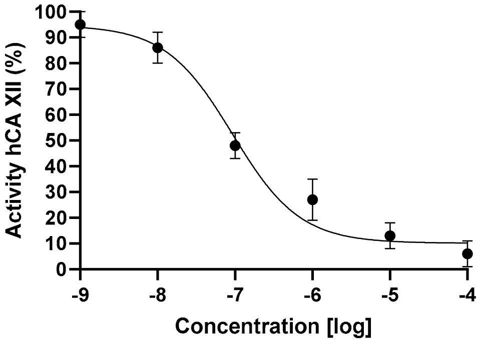

Compound 14

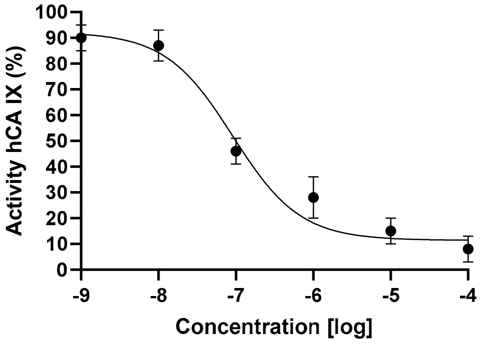

Compound 14

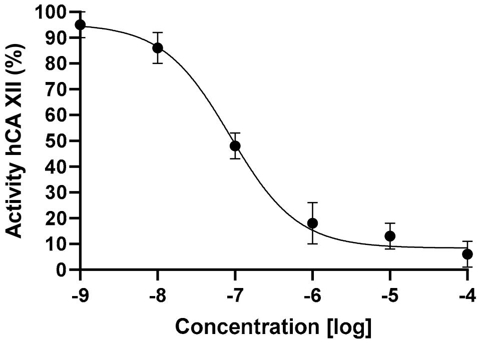

Compound 15

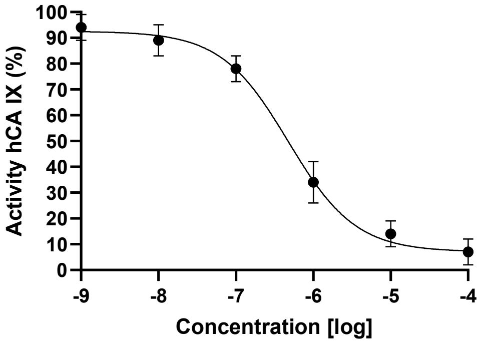

Compound 15

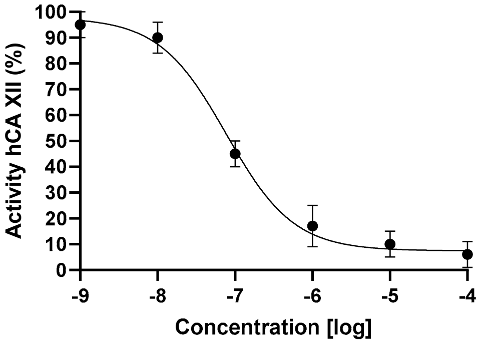

Table S2. Instrument parameters for exact mass determination

**Positive Ion Mode**	
Compound	**Instrument parameters**
**7**	Spray Voltage (|kV|): 4.50, Sheath Gas Flow Rate: 7, Aux Gas Flow Rate: 0, Swe[illegible] Gas Flow Rate: 0. Capillary Voltage (V): 30.00, Capillary Temperature: 270°C, T[illegible] Lens voltage (V): 50.00, Skimmer Voltage (V): 22.00.
**12**	Spray Voltage (|kV|): 4.50, Sheath Gas Flow Rate: 7, Aux Gas Flow Rate: 0, Swe[illegible] Gas Flow Rate: 0. Capillary Voltage (V): 67.50, Capillary Temperature: 270°C, T[illegible] Lens voltage (V): 95.00, Skimmer Voltage (V): 20.00.
**13**	Spray Voltage (|kV|): 4.50, Sheath Gas Flow Rate: 7, Aux Gas Flow Rate: 0, Swe[illegible] Gas Flow Rate: 0. Capillary Voltage (V): 32.50, Capillary Temperature: 270°C, T[illegible] Lens voltage (V): 85.00, Skimmer Voltage (V): 24.00.
**14**	Spray Voltage (|kV|): 2.50, Sheath Gas Flow Rate: 7, Aux Gas Flow Rate: 0, Swe[illegible] Gas Flow Rate: 0. Capillary Voltage (V): 60.00, Capillary Temperature: 270°C, T[illegible] Lens voltage (V): 100.00, Skimmer Voltage (V): 32.00.
**Negative Ion Mode**	
Compound	**Instrument parameters**
**9**	Spray Voltage (|kV|): 2.50, Sheath Gas Flow Rate: 5, Aux Gas Flow Rate: 2, Swee[illegible] Gas Flow Rate: 0. Capillary Voltage (V): -90.00, Capillary Temperature: 200°C, T[illegible] Lens voltage (V): -90.00, Skimmer Voltage (V): -26.00.
**11**	Spray Voltage (|kV|): 2.50, Sheath Gas Flow Rate: 5, Aux Gas Flow Rate: 2, Swee[illegible] Gas Flow Rate: 0. Capillary Voltage (V): -90.00, Capillary Temperature: 200°C, T[illegible] Lens voltage (V): -90.00, Skimmer Voltage (V): -26.00.

Table S2. Purification conditions for compounds **2-15**.

Entry	Structure	Purification method	
		Chromatography	Crystallization
**1**	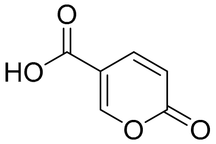	Commercial	/
**2**	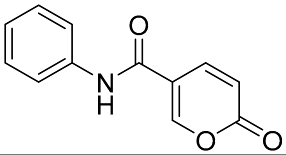	Stationary phase: silica gel Mobile phase: ethyl acetate/cyclohexane (33% *v*/*v*)	
**3**	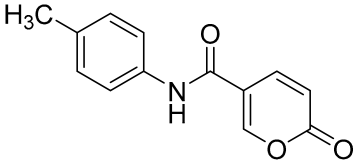	Stationary phase: silica gel Mobile phase: ethyl acetate/cyclohexane (33% *v*/*v*).	Mixture of dichloromethane/cyclohexane (4:1 ratio v/v).
**4**	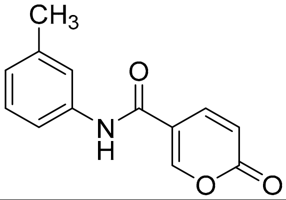	Stationary phase: silica gel Mobile phase: ethyl acetate/cyclohexane (33% *v*/*v*).	Mixture of dichloromethane/cyclohexane (4:1 ratio *v*/*v*).
**5**	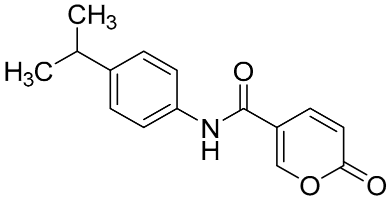	Stationary phase: silica gel Mobile phase: ethyl acetate/cyclohexane (33% *v*/*v*).	Mixture of dichloromethane/cyclohexane (4:1 ratio *v*/*v*).
**6**	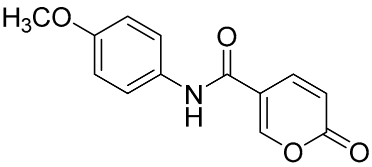	Stationary phase: silica gel Mobile phase: ethyl acetate/cyclohexane (33% *v*/*v*).	Mixture of dichloromethane/cyclohexane (4:1 ratio *v*/*v*).
**7**	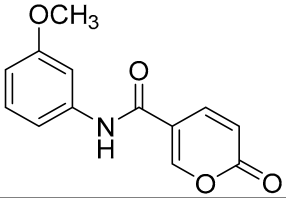	Stationary phase: silica gel Mobile phase: ethyl acetate/cyclohexane (33% *v*/*v*).	Mixture of dichloromethane/cyclohexane (4:1 ratio *v*/*v*).
**8**	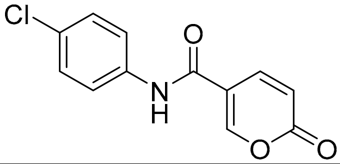	Stationary phase: silica gel Mobile phase: ethyl acetate/cyclohexane (33% *v*/*v*).	Mixture of dichloromethane/cyclohexane (4:1 ratio *v*/*v*).
**9**	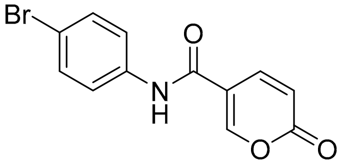	Stationary phase: silica gel Mobile phase: ethyl acetate/cyclohexane (33% *v*/*v*)	Mixture of dichloromethane/cyclohexane (4:1 ratio *v*/*v*). Performed two-times.

**10**	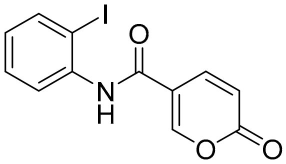	Stationary phase: silica gel Mobile phase: ethyl acetate/cyclohexane (33% *v/v*)	Mixture of dichloromethane/cyclohexane (4:1 ratio *v/v*).
**11**	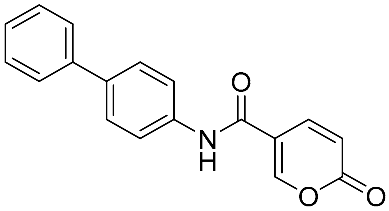	Stationary phase: silica gel Mobile phase: ethyl acetate/cyclohexane (33% *v/v*).	Mixture of dichloromethane/cyclohexane (4:1 ratio *v/v*). Performed two-times.
**12**	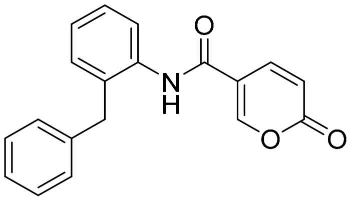	Stationary phase: silica gel Mobile phase: ethyl acetate/cyclohexane (33% *v/v*)	Mixture of dichloromethane/cyclohexane (4:1 ratio *v/v*). Performed two-times.
**13**	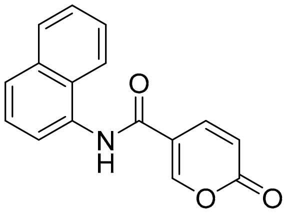	Stationary phase: silica gel Mobile phase: ethyl acetate/cyclohexane (33% *v/v*)	Mixture of dichloromethane/cyclohexane (4:1 ratio *v/v*). Performed two-times.
**14**	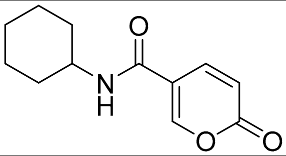	Stationary phase: silica gel Mobile phase: ethyl acetate/cyclohexane (33% *v/v*)	Mixture of dichloromethane/cyclohexane (4:1 ratio *v/v*). Performed two-times.
**15**	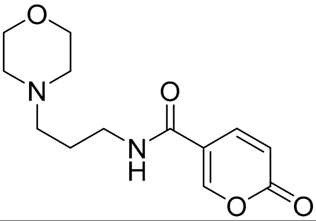	Stationary phase: silica gel Mobile phase: ethyl acetate/cyclohexane (33% *v/v*)	Mixture of dichloromethane/cyclohexane (4:1 ratio *v/v*). Performed two-times.

High resolution mass analysis of compound 7

C:\Xcalibur\...\Secci\Compound7 6/23/2022 5:21:23 PM

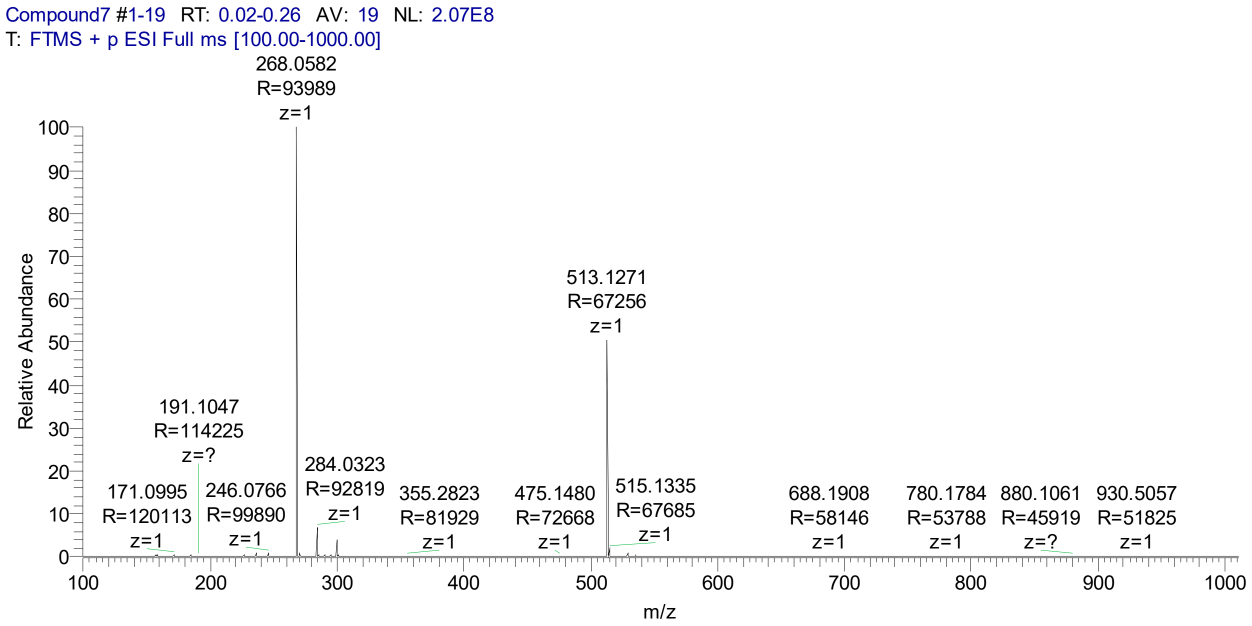

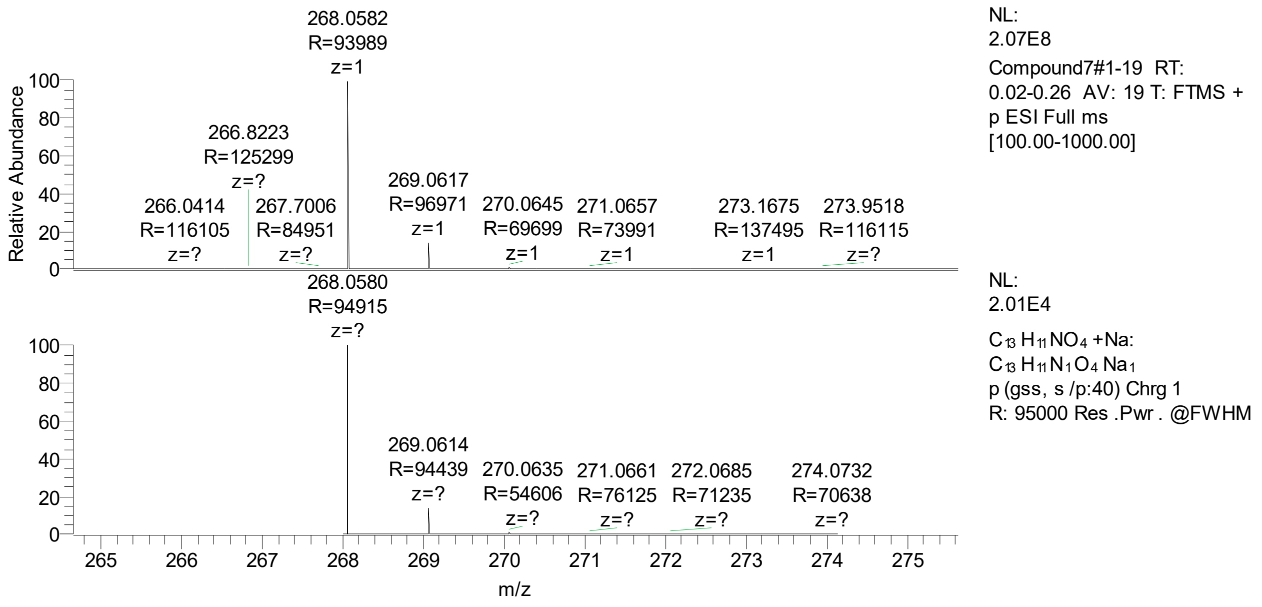

High resolution mass analysis of compound 9

C:\Xcalibur\...\Secci\Compound9neg 6/23/2022 5:37:39 PM

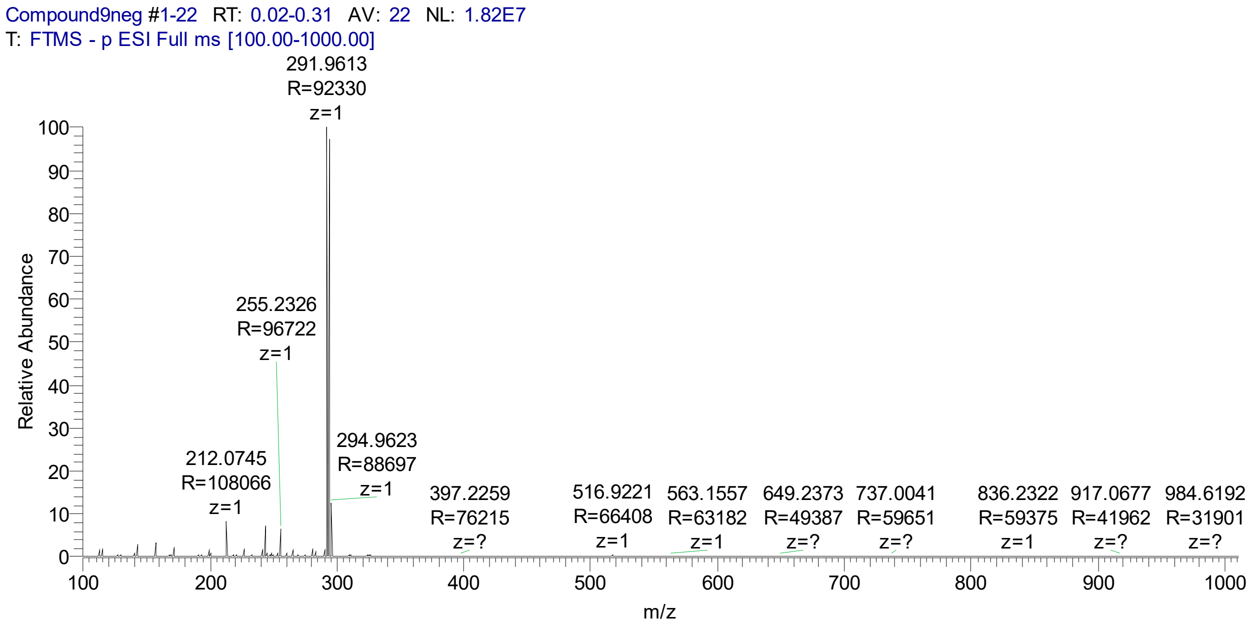

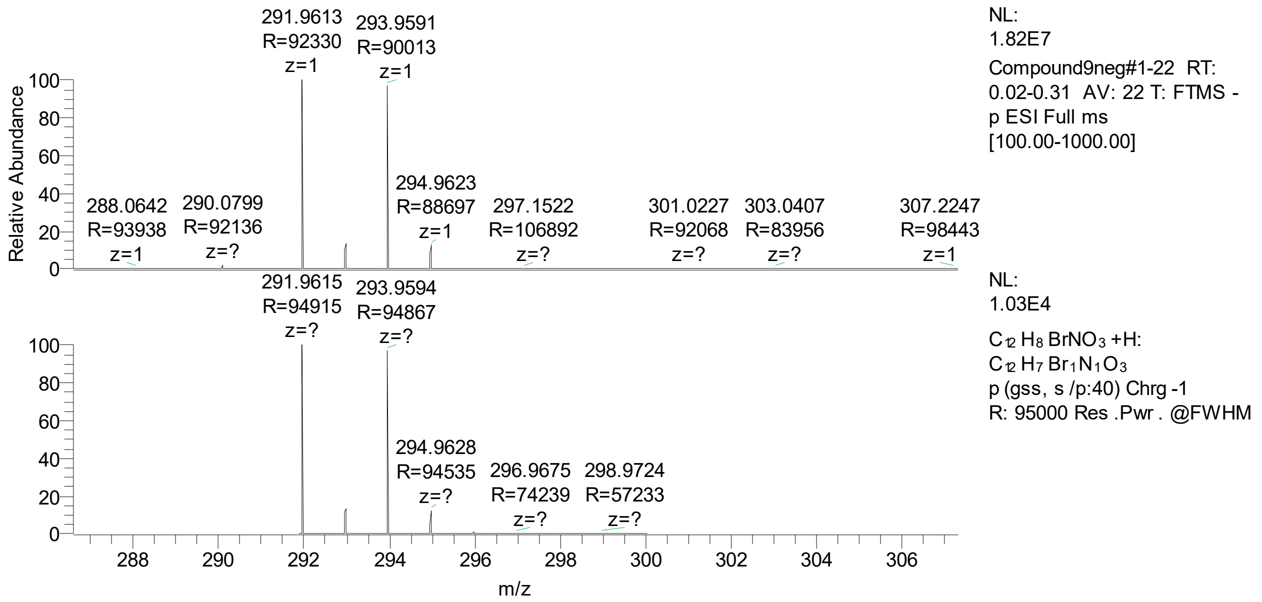

High resolution mass analysis of compound 11

C:\Xcalibur\...\Secci\Compound11neg 6/23/2022 5:47:53 PM

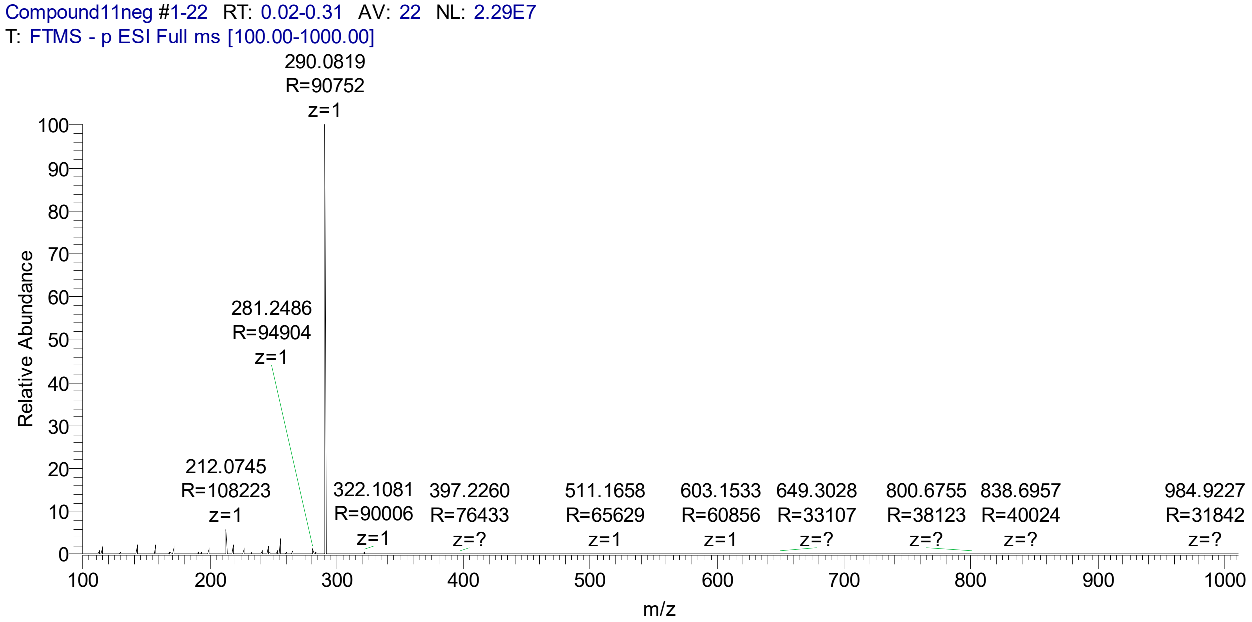

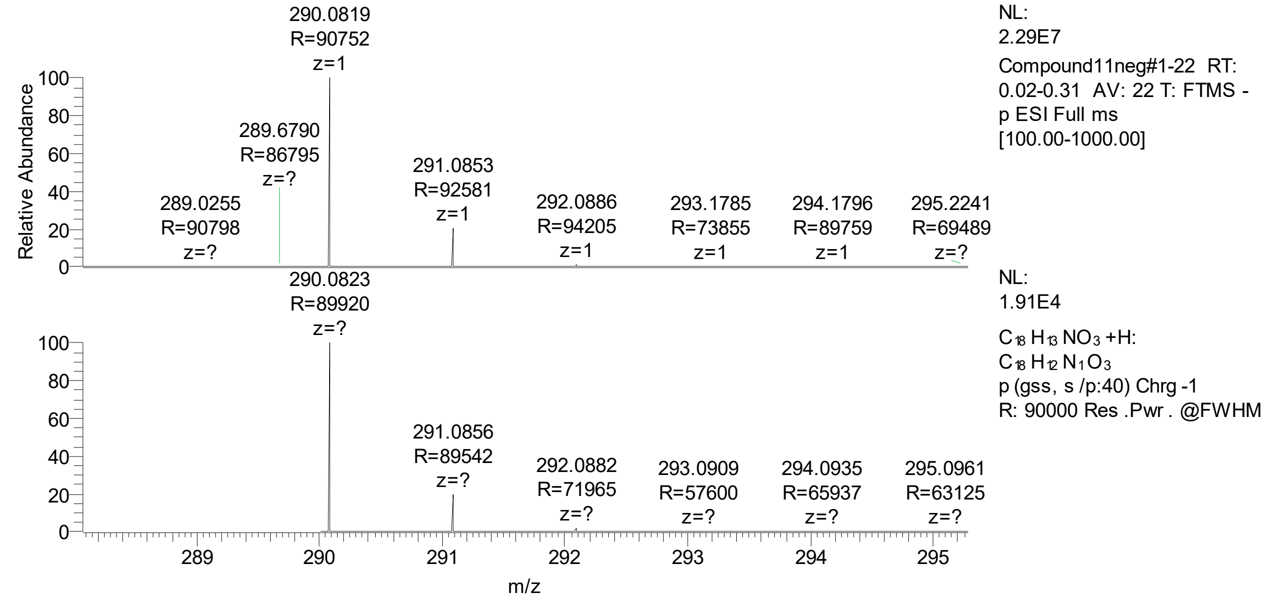

High resolution mass analysis of compound 12

C:\Xcalibur\...\Secci\Compound12 6/23/2022 5:55:28 PM

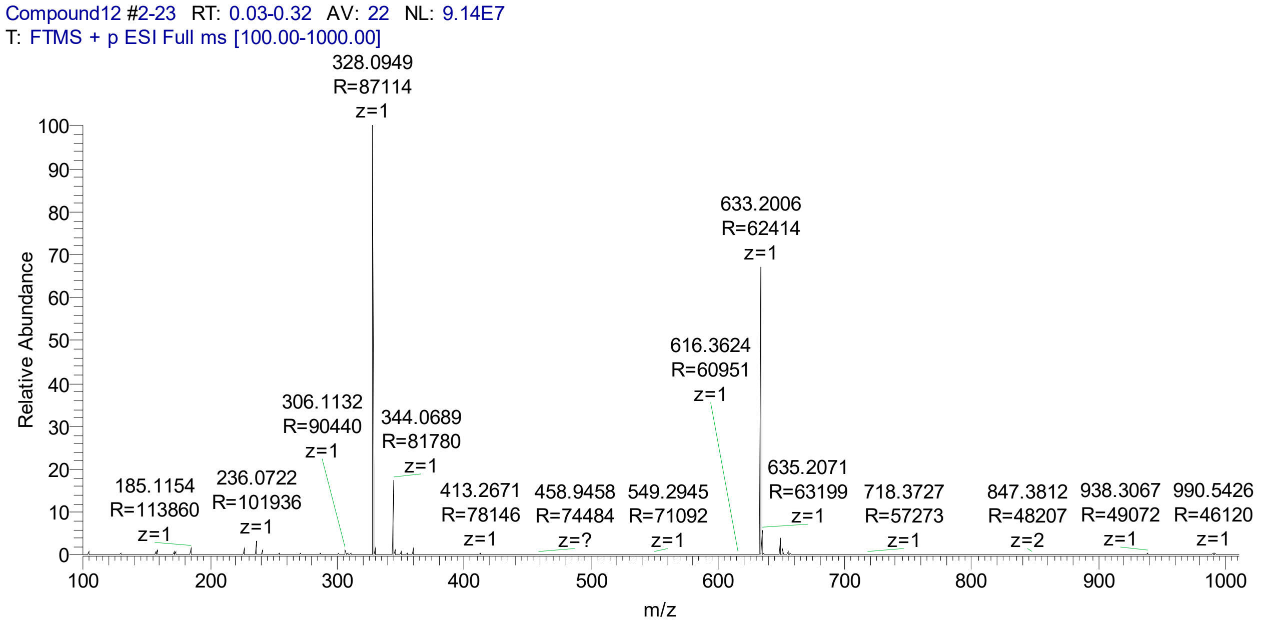

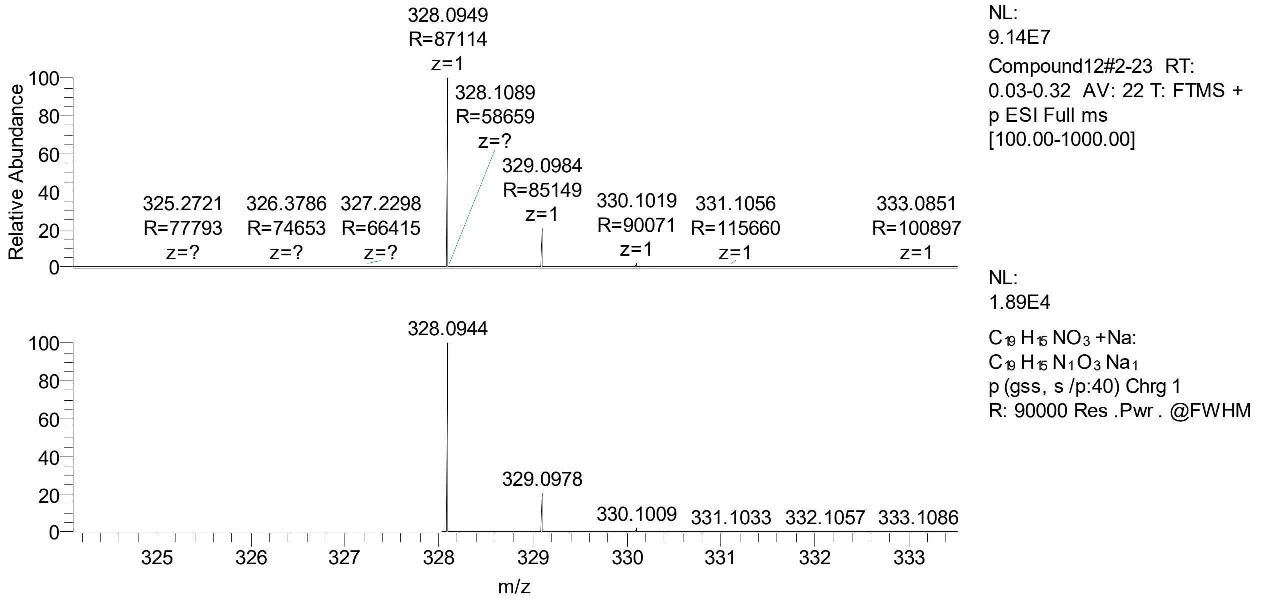

High resolution mass analysis of compound 13

C:\Xcalibur\...\Secci\Compound13 6/23/2022 6:24:33 PM

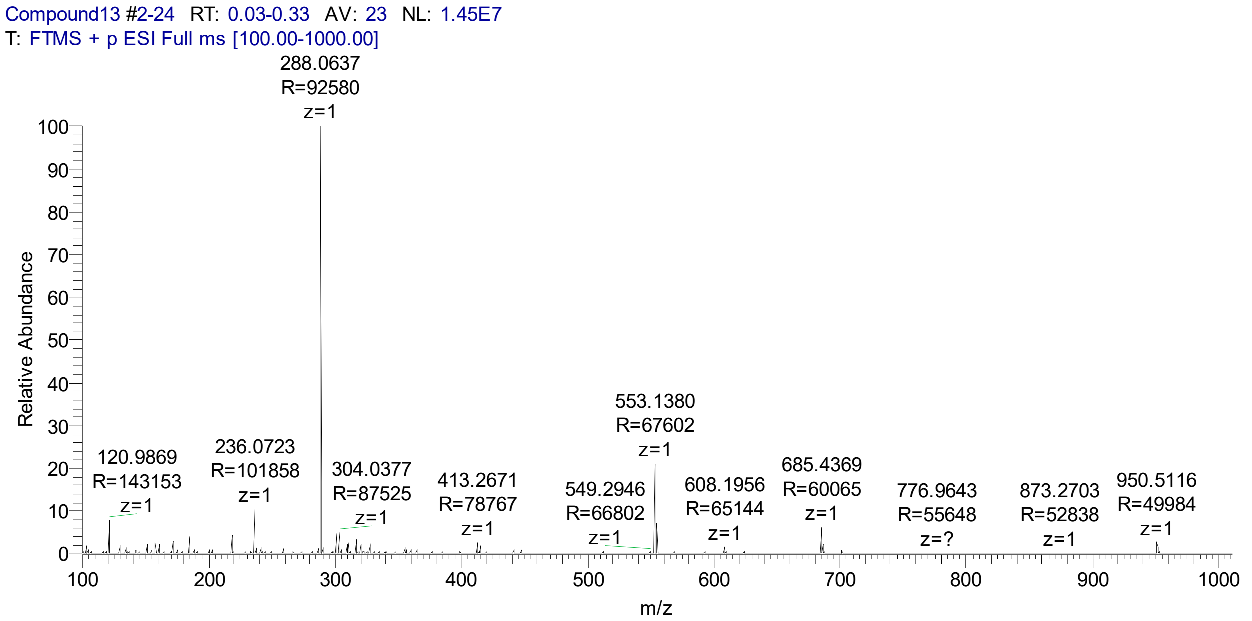

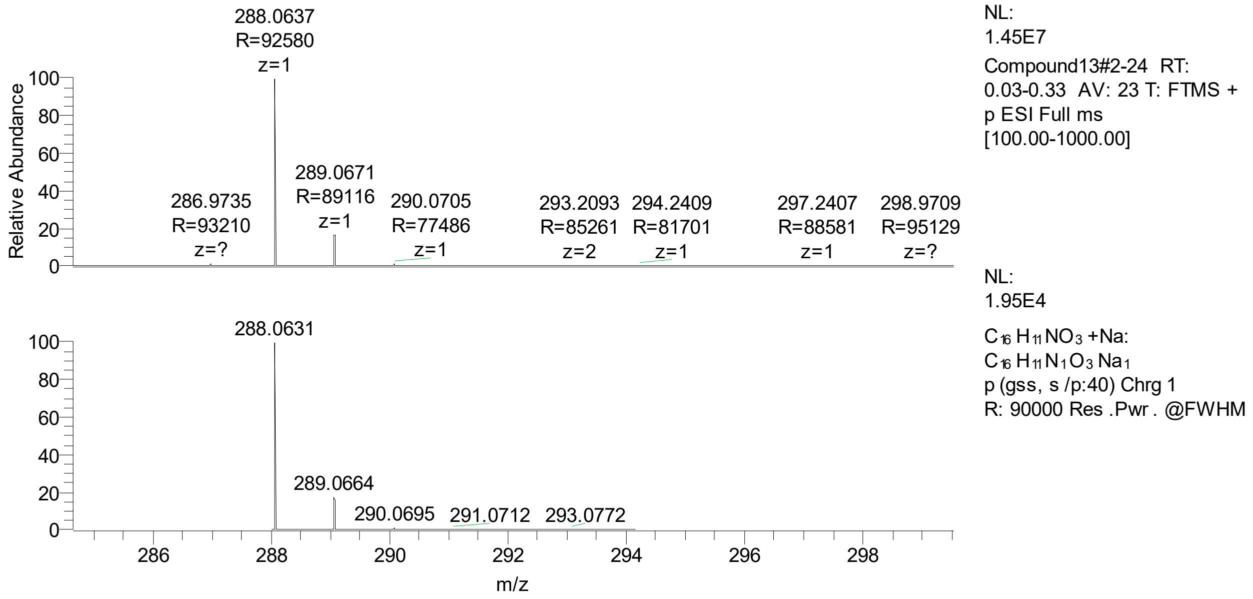

High resolution mass analysis of compound 14

C:\Xcalibur\...\Secci\Compound14 6/23/2022 6:35:15 PM

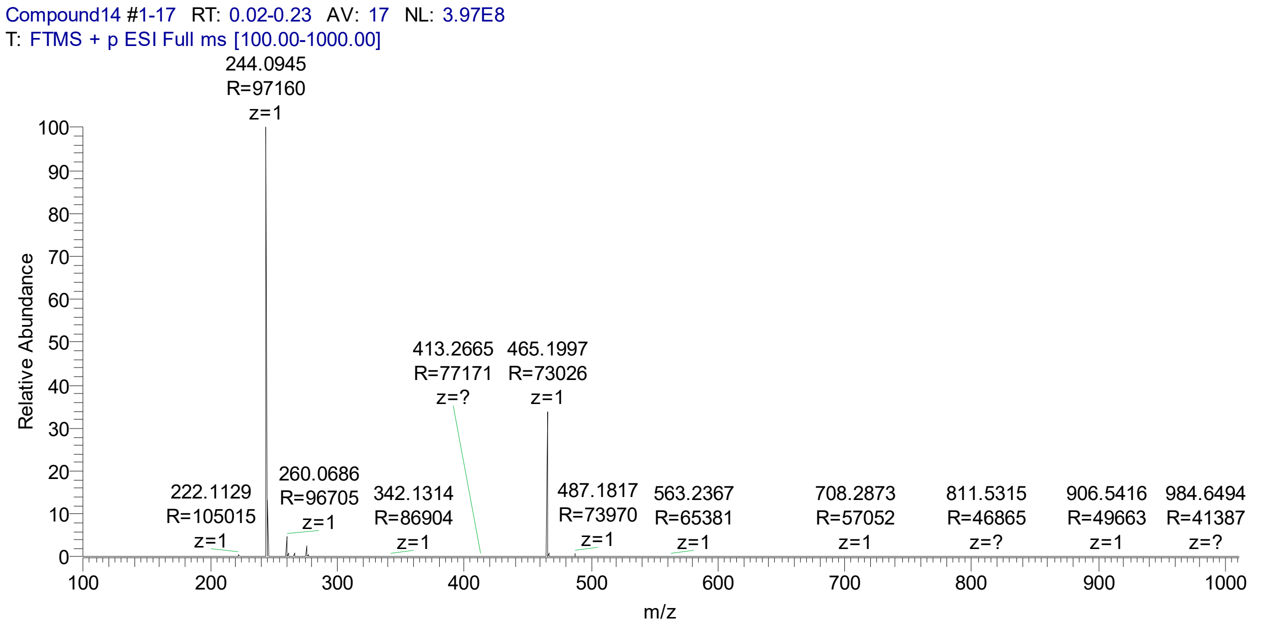

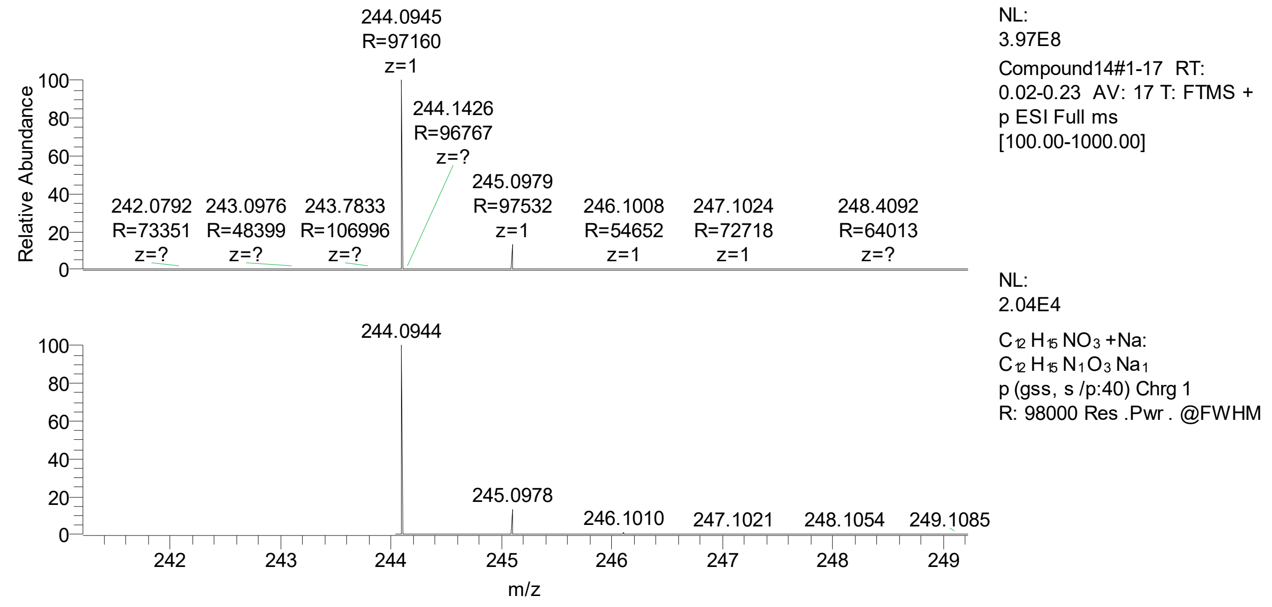

Supplement: Supplementary file 1 [file ijms-23-07950-s001.zip › ijms-1746202-supplementary.pdf]
